# 7th International Symposium on Molecular Allergology (ISMA 2017)

**DOI:** 10.1186/s13601-018-0193-z

**Published:** 2018-05-03

## Thursday 9 November 2017

### Poster Discussion Session I - Topic 1: Epidemiology of IgE sensitization profiles

#### P01 Sensitization profile to CCD-bearing proteins in a mediterranean polysensitized population

##### Olga Luengo^1^, Moises Labrador-Horrillo^1^, Anna Sala-Cunill^1^, Mar Guilarte, Paula Galvan^1^, Victoria Cardona^1^

###### ^1^Vall d’Hebron University Hospital, Barcelona, Spain

**Correspondence:** Olga Luengo - olgaluen@gmail.com

*Clinical & Translational Allergy* (*CTA*) 2018, **8(Suppl 1):**P01

**Background:** Cross-reactive carbohydrate determinants (CCDs) are a well described cause of non-clinically relevant in vitro cross-reactivity. The ISACTM microarray contains several allergen components purified from natural extracts that are recognized by anti-CCD sIgE (specific immunoglobulin E) and named “CCD-bearing proteins”: nPhl p4, nCyn d1, nJug r2, nCup a1, Cry j1, nPla a2.

We aimed to describe the relative frequency of recognition of CCD bearing proteins among polysenitized patients in order to aid in the interpretation of microarray results.

**Methods:** Two-thousand and two ImmunoCAP-ISAC 112 (Thermo Fisher) performed between 2012 and 2017 in both children and adult polysensitized patients. Values of the above mentioned CCD-bearing allergens > 0.1 ISU were considered positive. When 2 CCD components were positive in the absence of the genuine marker of the allergenic source (except for Cup a1), patients were categorized as “CCD-sensitized”.

**Results:** A total of 1120 patients’ sera (55.9%) recognized at least one CCD component, but only 22.2% of the patients were considered as “CCD-sensitized” according to our pre-defined criteria. Among the latter, the most frequently recognized CCD component was nPla a2 (86%), followed by nPhl p4 (84.5%), nCup a1 (82%), nJug r2 (79.3%), being MUXF3 the least recognized (37.2%).

Interestingly, the mean sIgE levels to the CCD components nPla a2, nPhl p4 and nJug r2 were significantly higher among those patients also sensitized to markers of genuine sensitization of the same allergen source (Table 1). Mean values of MUXF3 sIgE were 0.238 ISU, bellow the standard cutoff point of 0.35 ISU. nJug r2 positivity was more frequently detected as the only walnut allergen (39.4%), and thus a marker of CCD sensitization, or associated to walnut LTP rJug r3 (39.9%) but scarcely associated with rJug r1 (7.8%).

**Table 1:** Results:

	n Pla a 2		nPhl p 4		nJugr2	
	nPla a 2	nPla a 2 + rPla a 1	nPhl p 4	nPhl p 4 +Phl p1/Phl p 5/Phl p 6/Phl p 11	nJugr2	nJug r2 + rJugr1
Mean sIgE levels (ISU)	1.94	4.6	0.75	1.49	0.19	1.10
U Mann–Whitney sig.	0.00		0.00		0.00	

**Conclusions:** In these polysensitized patients, 22.2% are “CCD-sensitized”, being nPla a2 and nPhl p4 the most frequently detected and MUXF3 the least. This may be important when interpreting unexpected positivities in the allergen microarray. The mean serum CCD sIgE levels are generally low and in the case of MUXF3, commonly used as CCD marker, it would be misinterpreted as negative if the cutoff point of 0.35 ISU was used.

#### P02 Der P 1, Der P 2, Der P 23: house dust mite major allergens sensitization profile in a Portuguese population

##### Filipa Matos Semedo^1^, Elza Tomaz^2^, Ana Paula Pires^2^, Filipe Inácio^2^

###### ^1^Immunoallergology Department, Hospital de São Bernardo, Centro Hospitalar de Setúbal E.P.E.; Doctoral Programme, Faculdade de Ciências da Saúde, Universidade da Beira Interior, Setúbal; Covilhã, Portugal; ^2^Immunoallergology Department, Hospital de São Bernardo, Centro Hospitalar de Setúbal E.P.E., Setúbal, Portugal

**Correspondence:** Filipa Matos Semedo - pipa.semedo@gmail.com

*Clinical & Translational Allergy* (*CTA*) 2018, **8(Suppl 1):**P02

**Background:** House dust mites (HDM) represent one of the most important inducers of respiratory allergies worldwide, 85% of allergic asthmatic children being sensitized and higher specific IgE levels being related to likelihood of developing asthma and its severity. Recent studies have found high variability of frequencies in IgE recognition of HDM allergens. The aim of this study is to characterize a Portuguese population of HDM allergic patients concerning major allergens sensitization profile and its clinical relevance.

**Methods:** Sera of 98 paediatric and adult HDM-allergic patients, living in different areas, positive IgE to *Dermatophagoides pteronyssinus* total extract, were tested for Der p 1, Der p 2 and Der p 23 reactivity. Chi square Test was used to compare frequencies and T Test to mean IgE values.

**Results:** All patients had allergic rhinitis, 81% also asthma. Prevalence of IgE to each Der p 1, Der p 2 and Der p 23 was > 85%. Overall, 73 patients were sensitized to all three, 16 to two, 5 to just one (Der p 23 in 4 of them) and 4 to none. Patterns of IgE to Der p 2 and Der p 23 were similar encompassing adults and children, but Der p 1 reactivity was more frequent in children (91 vs 70%, *p* = 0.032). Also in paediatric group, mean IgE values were higher to all components, being significant for Der p 1 (56 vs 11 kUA/l, *p* = 0.012) and total Der p (109 vs 48 kUA/l, *p* = 0.018). No sex-related difference was found. Asthmatic patients in general had more frequent IgE response to Der p 1 (91%) and Der p 2 (89%), than nonasthmatics (68%; 68%, respectively: *p* = 0.009; *p* = 0.028). They also had higher mean IgE levels for total Der p (108 vs 56 kUA/l, *p* = 0.045). Asthmatic children who had started symptoms after 3 years old (32%) showed IgE values significantly higher than the others (Der p 1, *p* = 0.001; Der p 2 and Der p 23, *p* = 0.005). Furthermore, IgE levels to total Der p and Der p 2 were higher in children with more severe asthma (*p* = 0.007; *p* = 0.045) (Table 1).

**Table 1:** Results:

	Nonasthmatic subjects			Asthmatic subjects			Total
	Paediatric	Adult	Total	Paediatric	Adult	Total	
Patients, no. (%)	9	10	19	72	7	79	98
Sex (male/female), no.	6/3	4/6	10/9	51/21	4/3	55/24	65/33
Age range (years, minimum–maximum)	3–17	19–43	3–43	4–17	23–65	4–65	3–65
Mean age (years)	10	29	19	12	38	14	15
Mean IgE to total extract Der p (kU/L)	83.1	33.0	56.7	112.2	68.7	108.4	98.4
Mean IgE to Der p 1 (kU/L)	35.1	5.3	19.4	58.8	19.5	55.3	48.4
Mean IgE to Der p 2 (kU/L)	12.8	35.3	24.6	58.6	20.4	55.2	49.3
Mean IgE to Der p 23 (kU/L)	12.5	14.1	13.3	22.2	6.6	20.9	19.4

**Conclusions:** Our study shows a high prevalence of sensitization to Der p 23, also found in german and American studies, but relatively low IgE titers, similarly to other European countries, Canada and Japan. Cases of Der p 23 monosensitization have also been described in other cohorts. Children were more frequently sensitized to Der p 1 and had higher IgE to Der p 1 and total Der p. Asthma was related to more frequent recognition of Der p 1 and 2 and to higher IgE to total Der p. Asthma severity in children seemed associated to higher IgE to total Der p and Der p 2.

#### P03 Sensitization profiles to egg white allergen components in The United States of America

##### Andre Valcour^1^, Joseph Jones^2^, Jonas Lidholm^3^, Magnus Borres^3^

###### ^1^LabCorp, Burlington, NC, USA; ^2^Thermo Fisher Scientific, Phadia US Inc., Portage, MI, USA; ^3^Thermo Fisher Scientific, Uppsala, Sweden

**Correspondence:** Andre Valcour - valcoua@labcorp.com

*Clinical & Translational Allergy* (*CTA*) 2018, **8(Suppl 1):**P03

**Background:** Serological Egg White (EW) component testing has been applied in the diagnosis of human allergic disease. We have investigated the relationship between patient age and rates of sensitization to EW allergen components among EW sensitized individuals in the USA as well as correlation between component levels in EW sensitized subjects.

**Methods:** Sera from 7255 individuals with EW extract-specific IgE (sIgE ≥ 0.35 kUA/L) were analyzed by LabCorp for IgE to components Gal d 1/Ovomucoid (OM) and Gal d 2/Ovalbumin (OA).

**Results:** 93.1% of EW specific IgE positive (≥ 0.35 KUa/L) samples were positive for at least one of the components tested. 46.9% of these samples were positive for both. 6.7% were mono-sensitized to OM and 30.3% to OA. 39.0% of infants (0–3 years) while 23.4% if adolescents (12–15 years) were OA mono-sensitized. The correlation (r squared) between OM and OA was poor (< 0.32). Correlation between OM and OA with EW IgE were 0.52 and 0.75, respectively. The correlation between the sum of the components (OM + OA) versus EW IgE was 0.81.

**Conclusions:** Sensitization to individual EW components is highly dependent on patient age. A large percentage of EW sensitized individuals are mono-sensitized to OA. This is especially true for infants. There was poor correlation between individual components suggesting that the measurement of each might provide unique clinical insight. The correlation between the sum of components and the EW extract levels suggests that the components tested represent the antigens in the extract effectively.

#### P04 German cockroach component analysis reveals new major allergens in a US population

##### Jill Glesner^1^, Véronique Schulten^2^, April Frazier^2^, Lisa D Vailes^1^, Sabina Wünschmann^1^, Martin D Chapman^1^, Alessandro Sette^2^, Anna Pomés^1^

###### ^1^Indoor Biotechnologies, Inc, Charlottesville, VA, USA; ^2^La Jolla Institute for Allergy and Immunology, La Jolla, CA, USA

**Correspondence:** Anna Pomés - apomes@inbio.com

*Clinical & Translational Allergy* (*CTA*) 2018, **8(Suppl 1):**P04

**Background:** The currently known cockroach allergen components do not account for the total IgE reactivity to cockroach extracts. The goal of this study was to assess the IgE reactivity to a wide panel of proteins from *Blattella germanica* that are known or potential allergens to cockroach sensitized patients from the United States (US).

**Methods:** German cockroach allergens Bla g 1, Bla g 2, Bla g 4, Per a 7, Bla g 9 and Bla g 11 were expressed in *Pichia pastoris*. Bla g 1, Bla g 2 and Per a 7 were purified by specific-antibody affinity chromatography. Bla g 4 was purified by phenol Sepharose chromatography. Bla g 5 was expressed in Escherichia coli and purified by glutathione S-transferase affinity chromatography. Bla g 9 and Bla g 11 were purified by metal affinity chromatography. IgE antibody levels to these 7 purified allergens were measured by streptavidin ImmunoCAPs loaded with biotinylated purified allergens.

**Results:** The prevalences of IgE antibody reactivity to cockroach allergens in a population of US cockroach allergic patients (n = 16) were: 31% (Bla g 1), 56% (Bla g 2), 31% (Bla g 4), 50% (Bla g 5), 31% (Per a 7), 50% (Bla g 9) and 63% (Bla g 11). Bla g 9 and Bla g 11 were identified as major allergens, in addition to the currently known major allergens Bla g 2 and Bla g 5. In a sub-population of highly cockroach allergic patients (CAP class 3–5) (n = 12) the IgE prevalences for the major allergens were 67% (Bla g 2), 50% (Bla g 5), 58% (Bla g 9) and 75% (Bla g 11).

**Conclusions:** The identification of new major allergens in a cockroach allergic population needs to be taken into consideration for B cell component analysis and data interpretation in immunotherapy trials.

#### P05 Exploring the allergic sensitization prevalence across different countries worldwide by means of a new multiplex diagnostic tool

##### Claudia Alessandri^1^, Claudia Adriana Nicolae^2^, Silvana Miškovic^3^, Boleslaw Samolinski^4^, Krzysztof Buczylko^5^, Adrian Wu^6^, Raheleh Shokouhi Shoormatsi^7^, Rosetta Ferrara^1^, Danila Zennaro^1^, Maria Livia Bernardi^1^, Chiara Rafaiani^1^, Michela Ciancamerla^1^, Maurizio Tamburrini^8^, Ivana Giangrieco^8^, Lisa Tuppo^8^, Elena Camelia Berghea^2^, Zahra Pourpak^7^, Emilia Majsiak^9^, Maria Antonietta Ciardiello^8^, Adriano Mari^1^

###### ^1^Centri Associati di Allergologia Molecolare - CAAM, Rome, Italy; ^2^Clinica Medicum, Bucharest, Romania; ^3^Poliklinika Analiza, Dugopolje, Croatia; ^4^Warszawskie Centrum Alergologii ALERGO-MED, Warszawa, Poland; ^5^NZOZ Centrum Alergologii, Lódz, Poland; ^6^Ksena Healthcare, Hong Kong, Hong Kong SAR; ^7^Immunology, Asthma, and Allergy Research Institute, Tehran University of Medical Sciences, Teheran, Iran; ^8^Istituto di Bioscienze e Biorisorse, Consiglio Nazionale delle Ricerche, Naples, Italy; ^9^EMMA MTD, Lublin, Poland

**Correspondence:** Adriano Mari - adriano.mari@caam-allergy.com

*Clinical & Translational Allergy* (*CTA*) 2018, **8(Suppl 1):**P05

**Background:** IgE allergic diseases are progressively affecting more and more people globally. The allergenic sensitization may differ from one geographic area to another. Comparing the IgE sensitization prevalence requires a test campaign based on a broad panel of identical allergenic preparations. Objective: To investigate the different profiles of sensitization in six different Countries, namely Croatia (HR), Hong Kong (HK), Iran (IR), Italy (IT), Poland (PL), and Romania (RO).

**Methods:** The patients’ sensitizations have been investigated by means of the FABER test, bearing 244 allergens, both molecules (122) and extracts (122), including inhalant and food allergens, being therefore the most comprehensive tool for such a purpose. Serum samples from 1186 patients were tested in a single lab for the routine diagnostic workup. Enrolled subjects in the six groups were as follows: IT 385, RO 285, HR 210, PL 139, HK 104, IR 63.

**Results:** The sensitization for all dust mite allergens appears to be homogeneous in the six groups with the highest values in HK for Der p 2 (66%) and the lowest in RO for Der p 9 (2%). Der p 23, showed a significant difference in prevalence between HK (46%) and RO (5%). Fagales, Parietaria, Olive, Grass and Cypress pollen marker allergens were as follows (%): Bet v 1 PL 33, IT 13, HR 9, HK 7, HR 18, RO 5, IR 3), Par j 2 (IT 15, HK 11, PL 6, HR 5, RO 2, IR 0), Ole e 1 (IT 21, IR 17, HR 10, PL 8, RO and HK 3), Cup a 1 (IR 48, IT 34, PL 32, HR 27, RO 22, HK 19), Phl p 1 (IR 46, IT 37, PL 34, HK 22, RO 20, HR 15), Phl p 5 (IT 24, PL 21, HK 12 RO 9, HR 7 IR 6), and Amb a 1 (RO 18, PL 13, HR 9, HK 7, IR 2, IT 2). The allergenic molecules bearing CCD have a higher prevalence in PL patients, whereas LTP sensitization was recorded higher in IT (19) and PL (11) Other allergen like Can f 1 and Fel d 1 were most recognized by PL patients (20 and 42, respectively) and less by IR patients (2 and 13, respectively).

**Conclusions:** Although the low number of observations could lead to unwanted biases in our survey, IgE detection by means of the FABER test allows detecting sensitization prevalence to inhalant and food allergens using data from the routine diagnosis. The analysis discloses differences and similarities between countries worldwide, each characterized by different climates and eating habits. The analysis of larger cohorts of patients will be possible by the using data from a higher number of routine diagnostic samples.

#### P06 Sensitization profiles to milk allergen components in The United States of America

##### Andre Valcour^1^, Joseph Jones^2^, Jonas Lidholm^3^, Magnus Borres^3^

###### ^1^LabCorp, Burlington, NC, USA; ^2^Thermo Fisher Scientific, Phadia US Inc., Portage, MI, USA; ^3^Thermo Fisher Scientific, Uppsala, Sweden

**Correspondence:** Andre Valcour - valcoua@labcorp.com

*Clinical & Translational Allergy* (*CTA*) 2018, **8(Suppl 1):**P06

**Background:** Serological milk component testing has been applied in the diagnosis of human allergic disease. We have investigated the relationship between patient age and rates of sensitization to milk allergen components among subjects with suspected milk allergy across the USA as well as correlation between component levels in milk sensitized subjects.

**Methods:** Sera from 8402 individuals with milk (f2) extract-specific IgE (sIgE) ≥ 0.35 kUA/L were analyzed by LabCorp for IgE to milk components Bos d 4/alpha-lactalbumin (LA), Bos d 5/beta-lactoglobulin (LG) and Bos d 8/casein (CA).

**Results:** 83.4% of milk specific IgE positive (≥ 0.35 KUa/L) samples were positive for at least one of the components tested. 27.5% of these samples were positive for all three components. 9.0% were mono-sensitized to LB, 16.4% to LG and 7.8% to CA. The positivity rates for milk (f2) IgE and for the individual milk components decreased with age. The correlation (r squared) between individual components was poor (< 0.37) for all component pairs. Correlation between LA or LG and milk IgE were both < 0.51. The correlation between CA and milk IgE was 0.75 and between the sum of the three components (LB + LG + CA) versus milk IgE was 0.85.

**Conclusions:** Sensitization to individual milk components is highly dependent on patient age. There was poor correlation between individual components suggesting that the measurement of each might provide independent clinical information. The correlation between the sum of components and the milk extract levels suggests that the components tested represent the antigens in the extract relatively well.

#### P07 Molecular diagnosis of the most common aeroallergens in Iranian allergic patients

##### Raheleh Shokouhi Shoormatsi^1^, Chiara Rafaiani^2^, Mostafa Moin^1^, Mohammad Reza Fazlollahi^1^, Anoshirvan Kazemnejad^3^, Maryam Mahloojirad^1^, Javid Morad Abbasi^4^, Zahra Pourpak^1^, Adriano Mari^2^

###### ^1^Immunology, Asthma, and Allergy Research Institute, Tehran University of Medical Sciences, Tehran, Iran; ^2^CAAM - Centri Associati di Allergologia Molecolare, Rome, Italy; ^3^Department of Biostatistics, Faculty of Medical Sciences, Tarbiat Modares University, Tehran, Iran; ^4^Asthma and Allergy Center, Tehran Medical Sciences Branch of Academic Center for Education, Culture and Research (ACECR), Tehran, Iran

**Correspondence:** Adriano Mari - adriano.mari@caam-allergy.com

*Clinical & Translational Allergy* (*CTA*) 2018, **8(Suppl 1):**P07

**Background:** The development and advances in molecular diagnosis can help to epidemiologic and diagnostic studies concerning the determination of IgE sensitization profile against different allergenic components in each region. The objective of the present study was the molecular diagnosis of common aeroallergens using FABER test in Iranian allergic patients.

**Methods:** In this cross sectional study, 60 patients with allergic diseases including asthma, allergic rhinitis, and atopic dermatitis entered the study. Evaluating the molecular sensitization profile of serum samples was performed by using a multiplex method called FABER which is an in vitro test for assessment of specific IgE against allergenic extracts and molecules. Specific IgE to allergen components from 13 outdoor and 14 indoor sources were measured.

**Results:** Among patients, 61.7% were male. The mean (SD) age of patients was 30.73 (6.87) years. In primary tests with extract based methods, 62.7% showed multi-sensitization to inhalant allergens. The most common sensitizations to allergen extracts were Lol p (63.3%), Phl p (60%) and Pla a (51.7%), respectively but the sensitization to allergenic components revealed Lol p 1 (58.3%), Phl p 1.0102 (50%), Cup a 1 (48.3%) and Sal k 1 (36.7%) as the most frequent ones. Among indoor allergenic molecules, Der p 2 (35%), Der f 2 (33.3%) and Fel d 1 (13.3%) showed the highest prevalence. Co-sensitization to Lol p 1 and Phl p 1 was reported in thirty patients and 21 subjects showed sensitization to both Lol p 1 and Cup a 1 molecules. Interestingly, the males showed higher sensitization prevalence to outdoor allergens (94.6%) compared to females (65.2%) (*p* = 0.003). In contrast to outdoor allergens, the more sensitization to indoor allergens was observed in females (73.9%) rather than males (64.9%) although not statistically significant.

**Conclusions:** According to our results, a higher sensitization prevalence for outdoor allergenic extracts and components (83.3%) compared to indoor allergenic extract and components (68.3%) has been recorded in allergic patients in Iran.

**Acknowledgments:** We would like to acknowledge from European Academy of Allergy and Clinical Immunology (EAACI Research Fellowship Award 2014) to support this study.

#### P08 Prevalence of grass pollen sensitization among Armenian patients

##### Sevan Iritsyan^1^, Spartak Gambarov^2^

###### ^1^Center of Medical Genetics and Primary Health Care, Yerevan, Armenia; ^2^Yerevan State Medical University, Yerevan, Armenia

**Correspondence:** Sevan Iritsyan - iritsyan@gmail.com

*Clinical & Translational Allergy* (*CTA*) 2018, **8(Suppl 1):**P08

**Background:** In Armenia, grass pollen is the most common allergen responsible for IgE-mediated allergies. The aim of the study is to evaluate the rate of sensitization to grass pollen molecules in Armenia among the patients with seasonal allergic rhinitis.

**Methods:** 251 patients were diagnosed with seasonal allergic rhinitis based on clinical manifestation during 2015–2017 (ages 5–78, mean—26, and f/m = 1.145). The clinical symptoms were assessed according to ARIA guidelines. All patients’ diagnosis was tested by serum specific IgE testing with ImmunoCAP.

**Results:** 245 patients were tested for grass pollen sensitization. In 137 patients mild-to-severe sensitization (0.35–100 kAU/l) was detected to both extracts and molecules. In 60 patients (24.5%) we found mono-sensitization to g213 (Phl p1, Phl p5) and only in 5 (2%) patients were sensitized to both grass specific g213 (Phl p1, Phl p5) and panallergens g214 (Phl p7, Phl p12).

**Conclusions:** So, grass pollen is the most frequent cause of allergic rhinitis.

The revealed pattern of molecular sensitization reduces the rate of misdiagnoses. Also it gives an opportunity to make precise immunotherapy via excluding cases of cross-reactions.

#### P09 A comparison of the sensitization to ambrosia in the western and in the central part of Ukraine and an assessment of the sublingual immunotherapy efficacy in these patients

##### Svitlana Zubchenko^1^, Olena Sharikadze^2^, Olena Moskovenko^3^, Sergey Yuriev^4^, Fernando Pineda^5^

###### ^1^Danylo Halitsky Lviv National Medical University, Lviv, Ukraine; ^2^Shupyk National Medical Academy of Postgraduate Education, Kyiv, Ukraine; ^3^National Medical University named after A.A. Bogomolets, Kyiv, Ukraine; ^4^Ukrainian School of Molecular Allergology and Immunology, Kyiv, Ukraine; ^5^Diater Laboratorios, Madrid, Spain

**Correspondence:** Svitlana Zubchenko - svitlana_zu@meta.ua

*Clinical & Translational Allergy* (*CTA*) 2018, **8(Suppl 1):**P09

**Background:** The prevalence of *Ambrosia artemisiifolia* on the territory of Ukraine had started in 60–70 Th years last century from Crimea. Nowadays *Ambrosia* is registered in 23–24 regions of Ukraine including the Central Part, the collecting area are 1,328,377,863 ha. In Western Ukraine it appeared more than 20 years ago and its area increasing quickly.

**Aim** To compare the features of sensitization to Ambrosia in patients from Lviv (Western Ukraine) and Kyiv regions (Central Ukraine) and to analyze the efficacy of AIT with Extract of Ambrosia (Diater Laboratories, Spain).

**Methods:** 586 patients aged 5–58 were examined in Kyiv 327 (55.8%) and 259 (44.2%) in Lviv region. SPT was performed by Extract “Ambrosia”. The patients were examined to undergo molecular diagnostics using ImunoCAP (Phadia) to identify major (n Amb a1) allergen. The SLIT was carried out with a mixture of Ambrosia.

**Results:** The prevalence of Ambrosia sensitization diagnosed in 25 (9.6%) persons in the Lviv region. 3 (12.0%) children moved from the Crimea and 22 (88.0%) adults was born and have been living in Western Ukraine. Sensitization to Ambrosia major allergen (n Amb a1) was detected in 23 (92.0%) persons. Withal, sensitization to Ambrosia (SPT) in patients in the Kyiv region was determined 2.5 times higher: positive SPT were detected in 80 (24.5%) patients (28 (35.0%) children and 52 (65.0%) adults). The true sensitization has been confirmed in 88.0% of people. SLIT were prescribed in the patients from both regions. The efficacy of SLIT was assessed by a visual analogue scale (VAS-up). An assessment of SLIT showed that there was a significant decrease in the severity of symptoms in the study groups as after 2 years of treatment (94.1 and 94.5%, respectively).

**Conclusions:** A high level of sensitization to major allergen (n Amb a1) Ambrosia in children and adults in Ukraine leads to a significant increase in allergic pathology. High efficacy of the SLIT provides the possibility of relative control of the prevalence of severe form of allergic diseases.

### Poster Discussion Session I - Topic 2: The basis of allergenicity

#### P10 Identification of IgE-binding epitopes on the surface of the non-specific lipid transfer protein Art V 3

##### Sabrina Wildner^1^, Iris Grießner^2^, Martina Dimuzio^2^, Eva Vejvar^2^, Sara Huber^2^, Michael Hauser^2^, Adriano Mari^3^, Mario Schubert^2^, Brandstetter Hans^2^, Gabriele Gadermaier^2^

###### ^1^University of Salzburg, Christian Doppler Laboratory for Biosimilar Characterization, Salzburg, Austria; ^2^University of Salzburg, Department of Molecular Biology, Division of Allergy and Immunology, Salzburg, Austria; ^3^Associated Centers for Molecular Allergology, Rome, Italy

**Correspondence:** Sabrina Wildner - sabrina.wildner@sbg.ac.at

*Clinical & Translational Allergy* (*CTA*) 2018, **8(Suppl 1):**P10

**Background:** Pollen of *Artemisia vulgaris* (mugwort) are an important elicitor of allergic reactions in late summer and autumn. Art v 3 is an allergen of mugwort pollen which belongs to the non-specific lipid transfer protein (LTP) family. The aim of the study is to solve the structure of Art v 3 and to identify the structural epitopes of Art v 3 using murine monoclonal antibodies.

**Methods:** Recombinant non-labeled and double-labeled (13C/15N) Art v 3.0201 were expressed in *E. coli* and purified using cation exchange chromatography. The three-dimensional structure of Art v 3 was solved by X-ray crystallography and resonance assignment was obtained by NMR spectroscopy. In addition, three Art v 3-specific murine monoclonal IgG antibodies (mAbs) were produced in hybridoma cells and purified using affinity chromatography. Binding affinities between Art v 3 and the mAbs were determined using the surface acoustic wave (SAW) technology. Cross-reactivity between the murine mAbs and the IgE from sera of mugwort allergic patients (n = 21) was investigated in an inhibition ELISA. Structural epitopes of Art v 3 were determined by NMR spectroscopy using the double-labeled Art v 3 and the murine mAbs.

**Results:** Recombinant Art v 3 was produced as a non-tagged protein. X-ray crystallography and NMR revealed a homodimeric assembly of Art v 3 containing four alpha-helices stabilized by four disulfide bonds per molecule. Binding affinities between Art v 3 and mAbs were in the nanomolar range. The binding to IgE from patients’ serum was inhibited with a mean of 69–82% by the murine monoclonal antibodies indicating an overlap of the binding sites. Hydrogen/deuterium exchange detected by NMR spectroscopy with a resolution on the individual residues allowed the identification of epitope regions on the surface of Art v 3.

**Conclusions:** Within this study we solved the 3-D structure of Art v 3 and identified potential IgE binding regions on the surface of Art v 3. These results will provide further insights into allergen cross-reactivity within the lipid transfer protein family.

**Acknowledgements:** The financial support by the Austrian Federal Ministry of Science, Research and Economy, the National Foundation of Research, Technology, and Development, and by a Start-up Grant of the Province of Salzburg is gratefully acknowledged.

#### P11 Homologous tropomyosins from shrimp and chicken: purification and allergenicity assessment

##### Julia Klueber^1^, Françoise Codreanu-Morel^2^, Thomas Holzhauser^3^, Stefanie Randow^3^, Joana Costa^4^, Thorsten Graf^1^, Tanja Scheuermann^1^, Markus Ollert^1^, Karin Hoffmann-Sommergruber^5^, Martine Morisset^2^, Annette Kuehn^1^

###### ^1^Luxembourg Institute of Health, Esch-Sur-Alzette, Luxembourg; ^2^National Unit of Immunology and Allergology, Centre Hospitalier de Luxembourg, Luxembourg, Luxembourg; ^3^Division of Allergology, Paul-Ehrlich-Institut, Langen, Germany; ^4^Faculdade de Farmácia da Universidade do Porto, Porto, Portugal; ^5^Department of Pathophysiology and Allergy Research, Medical University of Vienna, Vienna, Austria

**Correspondence:** Julia Klueber - julia.klueber@lih.lu

*Clinical & Translational Allergy* (*CTA*) 2018, **8(Suppl 1):**P11

**Background:** Seafood is one of the most common elicitors for food-allergic reactions while, among crustacean species, ingestion of prawn (*Penaeus monodon*) is considered as pre-dominant cause of adverse reactions. Tropomyosin, a muscle protein, is the major allergen in invertebrates such as crustaceans. Vertebrate tropomyosins are non-allergenic proteins, an observation which is not well understood.

The aim of this study was first to isolate both allergenic (native, recombinant) and non-allergenic tropomyosins and following, to compare those proteins at the biomolecular levels and as to their allergenicity.

**Methods:** Homologue tropomyosins from Black Tiger Prawn (*P. monodon*), chicken breast and leg muscle (*Gallus gallus*) were purified by column chromatography. Recombinant tropomyosins were expressed in *E. coli*, followed by protein purification. Purified proteins were compared by Edman degradation, mass spectrometry (MS), antibody-binding studies (immunoblot, ELISA) and circular dichroism analysis. Allergenicity was assessed by IgE-ELISA, basophil activation test (BAT) and skin testing using shrimp allergic patients.

**Results:** Tropomyosins were purified to homogeneity by column chromatography in a milligram scale. MS and Edman analysis confirmed the identity of all proteins as muscle tropomyosins. Circular dichroism analysis revealed characteristic alpha-helical structures as well as high protein stability towards thermal treatment. Specific IgE sera titer were up to 9-times higher to shrimp than to chicken tropomyosin. BAT was positive with shrimp allergens at 100-times lower allergen concentrations than with chicken homologs. Biomolecular assays on allergen characterization as well as IgE- and BAT-assays gave similar results for both native and recombinant proteins. In addition, skin reactivity of shrimp-allergic patients was positive with both shrimp and chicken tropomyosins but at up to 100-times lower concentrations with the shrimp allergen.

**Conclusions:** Tropomyosins from shrimp and chicken exhibit similar biomolecular characteristics while they vary by their allergenic potency. Both tropomyosins might be used as standard proteins, representing high and low allergenic molecules, in future experimental set-ups for the risk assessment of novel food sources.

#### P12 Aggregation of gliadins by thermal treatment decreases their allergenicity in vitro

##### Sandra Denery-Papini^1^, Roberta Lupi^1^, Florence Pineau^1^, Gilbert Deshayes^1^, Olivier Tranquet^1^, Stefania Masci^2^, Colette Larré^1^

###### ^1^INRA, Nantes, France; ^2^University of Tuscia, Viterbo, Italy

**Correspondence:** Sandra Denery-Papini - sandra.denery@inra.fr

*Clinical & Translational Allergy* (*CTA*) 2018, **8(Suppl 1):**P12

**Background:** Food processing, as well as digestibility and intestinal transport, are key factors to consider since they may affect the allergenic potential of food allergens. Typically, wheat based foods are always consumed after cooking which include some heating step. As regard to health aspects, wheat may trigger food allergy in some individuals. Numerous wheat allergens have been identified, and in particular the gliadins, that are among the main proteins responsible for food allergy to wheat.

Complex foods such as bread or pasta are not easy to handle in ‘in vitro’ assays for allergenicity evaluation. We used total gliadins and the alpha-gliadin sub-fraction as simplified models to investigate the effect of heating on their capacity to maintain an allergenic potential. Successive steps of the “antigen transformation” were taken into account, from heating treatment to gastric digestion before considering the passage of the intestinal barrier.

**Methods:** The heated and heated/digested total gliadins and alpha-gliadins were characterized for their size by laser light scattering. The chromatographic profiles of the soluble fractions were obtained by RP-HPLC chromatography. The IgE-binding capacity of the treated proteins was compared to that of the native forms with sera from wheat allergic patients. Furthermore their capacity to cross the intestinal barrier and to induce the mast cell degranulation was investigated by combining two in vitro cellular models, Caco-2 and RBL-SX38.

**Results:** The heat treatment of total gliadins or of alpha-gliadins induced in both cases the production of large aggregates that were no more recognized by patients IgE. However, after limited pepsin hydrolysis, they recovered partial IgE-binding by unmasking epitopes in Dot Blot, but were not able to trigger RBL cells. After crossing the Caco2 cells, the treated proteins partially recovered their biological activity.

**Conclusions:** The large aggregates of gliadins that can occur during bread-making displayed a decreased allergenicity in vitro compared to native gliadins. This may be related to the capacity of some patients to achieve hypo-responsiveness to wheat during oral immunotherapy protocols performed with bread or other heated wheat-based products.

#### P13 Scavenger receptor class a mediates uptake of Ara H 1, a major peanut allergen, by human M2 macrophages

##### Maren Krause^1^, Peter Crauwels^2^, Martin Globisch^3^, Thomas Henle^3^, Ger Van Zandbergen^2^, Stefan Vieths^1^, Stephan Scheurer^1^, Masako Toda^1^

###### ^1^VPr1 Research Group “Molecular Allergology“, Paul-Ehrlich-Institut, Langen, Germany; ^2^Division of Immunology, Paul-Ehrlich-Institut, Langen, Germany; ^3^Institute of Food Chemistry, Technical University Dresden, Dresden, Germany

**Correspondence:** Maren Krause - masako.toda@pei.de

*Clinical & Translational Allergy* (*CTA*) 2018, **8(Suppl 1):**P13

**Background:** Ara h 1 potentially contributes to peanut-induced anaphylactic reactions as a major peanut allergen. Dendritic cell-specific ICAM-grabbing nonintegrin (DC-SIGN) is described as receptor for Ara h 1 to activate human dendritic cells (Shreffler et al., J. Immulol. 2006), whereas Ara h 1-mediated activation of macrophages is less investigated. Since evidence has accumulated that not only dendritic cells but also macrophages play a crucial role in development and maintenance of food allergy, we aimed to investigate interaction of Ara h 1 with human primary macrophages.

**Methods:** M1 and M2 macrophages were generated by culturing peripheral blood derived monocytes from healthy donors in the presence of rGM-CSF and rM-CSF for 6-8 days, respectively. Ara h 1 was isolated from unroasted peanut. Levels of Ara h 1 uptake and receptor expression by macrophages were assessed by flow cytometry. The levels of secreted cytokines by Ara h 1-stimulated cells were assessed by ELISA. Interaction of Ara h 1 with receptors expressed on the cell surface of macrophages was investigated using inhibitors of putative cell surface receptors and small interfering RNA.

**Results:** Upon stimulation with Ara h 1, M1 macrophages produced higher levels of pro-inflammatory cytokines IL-6 and TNF-α than M2 macrophages. In contrast, M2 macrophages internalized Ara h 1 to a greater extent than M1 macrophages. M1 macrophage expressed DC-SIGN and SR-A only at marginal levels, whereas M2 macrophages expressed both receptors at considerable levels. Small interfering RNA knockdown of DC-SIGN in M1 and M2 macrophages did not suppress the uptake of Ara h 1 by the cells. However, inhibitors for scavenger receptor class A (SR-A), e.g. polyinosinic acid and acetylated low density lipoprotein, suppressed M2 macrophage-mediated, but not M1 macrophage-mediated uptake of Ara h 1.

**Conclusions:** In this study, we demonstrated that DC-SIGN is likely not to be a major receptor involved in the interaction of Ara h 1 by human primary macrophages. SR-A is demonstrated to partly mediate the interaction of Ara h 1 with M2 macrophages, which play an active role in the pathogenesis of allergy. Further studies are necessary to gain a deeper understanding of the interaction between Ara h 1 and M2 macrophages and to unravel the mechanism underlying the intrinsic allergenicity.

#### P14 Genetic variation influences the impact of PGE2 on allergic responses in murine mast cells

##### Shruti Rastogi, Maria Nassiri, Magda Babina, Margitta Worm

###### Allergy-Center-Charité, Department of Dermatology and Allergy, Charité - Universitätsmedizin, Berlin, Germany

**Correspondence:** Shruti Rastogi - shruti.rastogi@charite.de

*Clinical & Translational Allergy* (*CTA*) 2018, **8(Suppl 1):**P14

**Background:** Prostaglandin (PG) E2 plays an important role in relation to mast cells (MCs) in different diseases. It mediates various and sometimes opposing effects on these cells through activation of four distinct receptors (EP1-4). Responses can be influenced by several factors such as variation among species or tissue sites. Differences in the genetic background within a species might likewise contribute to the reactivity and response pattern of MCs towards PGE2.

**Methods:** In this study, we examined genetic variation as a factor influencing the responsiveness towards PGE2 in MCs from two mouse strains typically employed in studies of allergic diseases. We first analyzed serum levels of PGE2 in Balb/c and C57BL/6J mice. Then, the expression of EP1-4 receptors was determined using bone marrow-derived cultured mast cells (BMcMCs). Subsequently, we assessed the impact of various concentrations of PGE2 and specific EP-agonists alone/in combination on IgE-mediated MC activation by detection of histamine release (HR).

**Results:** Serum levels of PGE2 were significantly higher in Balb/c compared to C57BL/6 J mice. PGE2 receptors were likewise expressed to a greater extent in BMcMCs from Balb/c mice with the highest expression of EP3. PGE2 increased IgE-mediated HR in BMcMCs from Balb/c mice dose-dependently. In contrast, PGE2 led to an inhibition of HR in C57BL/6-derived MCs. EP receptor agonists achieved a comparable influence on HR in both strains. EP2-agonist decreased the IgE-mediated response while the EP3-agonist elevated it in both strains. By contrast, EP4-agonist had no impact on MC activation. However, a combination of EP2 and EP4-agonists, decreased HR in BMcMCs taken from C57BL/6J mice only.

**Conclusions:** In conclusion, BMcMCs from Balb/c and C57BL/6J mice exhibit heterogeneity in their responsiveness towards PGE2. PGE2 seems to increase MC degranulation via EP3 receptor, in Balb/c mice, while in C57BL/6 mice the inhibition of MC degranulation might be caused by simultaneous ligation of EP2 and EP4. Differences in the PGE2 network among genotypes may contribute to their differential susceptibility towards disease, as endogenous PGE2 has been implicated in the fine-tuning of allergic reactions. The current findings provide the basis to explore the modulation of MC signaling by the genetic background of the host.

#### P15 Intensity of deamidation in the epitopes of acid-hydrolyzed wheat proteins is a key parameter for their allergenicity

##### Olivier Tranquet^1^, Chantal Brossard^1^, Florence Pineau^1^, Roberta Lupi^1^, Kayoko Matsunaga^2^, Reiko Teshima^3^, Shinobu Sakai^3^, Jean-Charles Gaudin^1^, Colette Larré^1^, Sandra Denery-Papini^1^

###### ^1^INRA, Nantes, France; ^2^Fujita Health University School of Medicine, Aichi, Japan; ^3^National Institute of Health Sciences, Tokyo, Japan

**Correspondence:** Olivier Tranquet - olivier.tranquet@inra.fr

*Clinical & Translational Allergy* (*CTA*) 2018, **8(Suppl 1):**P15

**Background:** Acid-hydrolyzed wheat proteins (a-HWP) were used as ingredients in food and cosmetics. From the 2000’s, cases of severe food allergy to HWP have been reported in people tolerant to native wheat proteins. Denery et al. demonstrated that deamidation of wheat proteins, the main consequence of acid-hydrolysis, generates neo-epitopes responsible for this particular allergy to wheat [1]. More recently in Japan, a soap containing a-HWP elicited severe skin reactions and food allergy in more than 2000 people [2]. Gliadins and glutenins, the main components of wheat proteins, are characterized by homologous domains constituted of repeated sequences of 6–8 amino acids rich in glutamines. During acid-hydrolysis, the random process of deamidation results in heterogeneous deamidation in each repeated sequences [3]. This work investigated the effect of the deamidation rates of the repeated sequences of a-HWP on their triggering potency.

**Methods:** Three batches of deamidated gliadins were produced by increasing the acid-hydrolysis duration. These 3 samples and 5 industrial HWP samples involved in European or Japanese cases of allergy were characterized for their content in native, weakly deamidated and highly deamidated repeated sequences by competitive ELISA. Their triggering potency was determined using a basophils assay with HWP-allergic patients’ sera.

**Results:** Competitive ELISAs showed that native sequences were progressively converted to deamidated sequences when acid-hydrolysis duration increased. Among the deamidated sequences the content in highly deamidated sequences progressively increased with the treatment duration while the content in weakly deamidated sequence remained constant. Industrial HWPs appeared extremely heterogeneous and displayed various levels of native, weakly and highly deamidated sequences. The ability to activate basophils sensitized with HWP-allergic patients appeared related to the content in highly deamidated sequences.

**Conclusions:** Repeated domains of gliadins and glutenins in a-HWPs are a mix of native, weakly deamidated and highly deamidated sequences which proportions vary among the products released on the market. The content in highly deamidated sequences predominantly contributed to the triggering potency of a-HWP samples.

**References**

1. Denery-Papini, S. et al. Allergy 67, 1023–1032 (2012)
2. Nakamura, M. et al. Contact Dermatitis 74, 346–352 (2016)
3. Tranquet, O. et al. J. Agric. Food Chem. 63, 5403–5409 (2015)

#### P16 Compared degranulation abilities of in vitro digested native and thermally aggregated ovalbumin

##### Sandra Denery-Papini^1^, Mathilde Claude^1^, Gianluca Picariello^2^, Roberta Lupi^1^, Martine Drouet^3^, Colette Larré^1^, Chantal Brossard^1^

###### ^1^INRA, Nantes, France; ^2^Istituto di Scienze dell’Alimentazione, Avellino, Italy; ^3^CHU d’Angers, Unité Allergologie Générale, Angers, France

**Correspondence:** Sandra Denery-Papini - sandra.denery@inra.fr

*Clinical & Translational Allergy* (*CTA*) 2018, **8(Suppl 1):**P16

**Background:** Protein structures are often modified and aggregation of more or less unfolded proteins may occur upon heating. Depending on the balance of attractive and repulsive interactions during heating, protein aggregates of various morphologies formed. Ovalbumin (OVA) is the major egg-white protein and a major egg-white allergen. OVA aggregates were shown to be differently digested (extent of digestion, nature of released peptides) depending on their morphologies. In the context of food allergy to egg, how the degranulation ability changed during short-duration digestion process of native and aggregated OVA was studied.

**Methods:** OVA solutions were heated to form nanometric linear or micrometric spherical-agglomerated aggregates. Native and aggregated OVAs were in vitro digested using a gastrointestinal digestion model based on the INFOGEST harmonized protocol with a final degradation with peptidases of the jejunal brush border membranes (BBM) enzymes. Degranulation abilities were studied for the three OVAs and their digests using a pool of eight sera from egg-allergic children and the RBL-SX38 cell line.

**Results:** Undigested, both aggregates had similar degranulation abilities lower than the ability of native OVA. Native and aggregated OVAs exhibited a similar reduced ability at the end of the digestion but were differently affected during the digestion process. Heated aggregated OVAs were more and more rapidly digested than the native OVA and the small more than the large aggregates. The duodenal phase mostly participated to the digestion of the native OVA and no further digestion during the BBM phase was noticed.

The degranulation abilities of the aggregates slightly changed during the digestion process. Although digestibility differed between the aggregates, they exhibited similar degranulation abilities at each step of the digestion process. The degranulation ability of native OVA was mostly decreased by the duodenal digestion; only a small decrease was noticed during the gastric phase and no further change with BBM digestion.

**Conclusions:** Compared to OVA aggregates, both the higher degranulation capacity of undigested native OVA and its late reduction during the duodenal phase of the digestion process could be responsible for the better tolerance of heated OVA by egg-allergic patients.

#### P17 Morphofunctional characteristics of regulatory cell compartments in patients with bronchial asthma and concomitant Epstein–Barr viral infection

##### Anna Konishcheva, Valentina Gervazieva, Svetlana Shodova

###### Mechnikov Research Institute of Vaccines and Sera, Moscow, Russia

**Correspondence:** Anna Konishcheva - ankon81@list.ru

*Clinical & Translational Allergy* (*CTA*) 2018, **8(Suppl 1):**P17

**Background:** Bronchial asthma is considered as chronic disease with heterogenous biological mechanisms, resulting in persistent and usually progressive airway inflammation. The functional balance between effector and regulatory cells compartments is critically important in prevention both allergic and inflammatory processes and could be altered in conditions of concomitant chronic DNA viral infection. Epstein–Barr Virus (EBV), as an important human DNA virus, establishes a latent chronic infection of lymphocytes by most adults worldwide. Modulation of the host innate immune responses is a key component of EBV lifecycle, strongly associated with B cell tumors and autoimmunity. We addressed two issues: to determine the frequency of the EBV active infection in the airways and blood cells of asthma patients and delineate characteristics of Treg and Breg cells in severe asthma, which might have the pathophysiological relevance to EBV carriage.

**Methods:** PBMCs and oropharyngeal swabs were collected from 25 patients with severe asthma compared with 13 moderate asthma, 18 patients with allergic rhinitis and 10 healthy controls. EBV DNA load in upper airways and peripheral lymphocytes was measured using PCR assays. Blood samples following staining with anti-CD45, CD3, CD4, CD16, CD25 and CD19 Abs were analyzed with Cytomics FC 500 flow cytometer. Breg and Treg cells were gated from PBMC as CD5+ CD19+ and CD4+ CD25high, respectively and analyzed for FOXP3 and Annexin PI staining.

**Results:** The percentage of CD5+ CD19+ cells (identified as Breg) was lower in EBV negative asthma patients, compared to AR and healthy, whereas EBV DNA detection was strongly associated with significant increase of CD5+ CD19+ cells (4.8 ± 7.9%). Among CD5+ CD19+ , FOXP3 positive cells were detected in 25% only in control samples, whereas asthma group distinguished by weak expression of CD5+ CD19+ FOXP3 (3.5–4.9%). The apoptotic activity, evaluated by Annexin V-PE, was significantly higher within the population of FOXP3+ Breg cells (CD19+ CD5+ and CD5-) only in asthma and strongly correlated with EBV DNA carriage (*p* = 0.003). The frequency of CD4+ CD25high FOXP3 T cells among CD4 T cells was lower in patients with severe asthma compared with AR (n = 19), whereas the content of CD4+ CD25high FOXP3+ cells in severe asthma in 97% was Annexin +, compared to 57% versus non-asthmatics

**Conclusions:** High frequency of active EBV infection, associated with impairment of Treg and high apoptosis of Bregs, suggestively aggravating regulatory cells deficiency in severe asthma.

#### P18 Presence of icarapin (Api M 10) In VIT anallergo

##### Neri Orsi Battaglini^1^, Laura Salvini^2^, Cristina Tinti^2^, Maurizio Severino^3^

###### ^1^Anallergo, Florence, Italy; ^2^TLS, Siena, Italy; ^3^San Giovanni di Dio, Florence, Italy

**Correspondence:** Neri Orsi Battaglini - neri.orsibattaglini@anallergo.it

*Clinical & Translational Allergy* (*CTA*) 2018, **8(Suppl 1):**P18

**Background:** Reactions to honeybee stings range from small local reactions to large local reactions, up to anaphylaxis.

Among the best characterized honey bee allergenes, recently it was discovered icarapin (Api m 10). Besides it was demonstrated that honeybee VIT may be less efficacious because of the absence of this specific allergen, perhaps lost during processing of the venom extract. Api m 10 was not found in any measureable concentration in therapeutic honey bee venom (HBV) preparations, though it appears to be a significant allergen. These findings were supported by subsequent observations that in patients with dominant sensitization to Api m 10, IgE reactivity to HBV (sIgE) could be inhibited by crude HBV preparations but not by therapeutic HBV preparations.

Frick et al. demonstrated that patients with a predominant sensitization to Api m 10 are at a higher risk of VIT treatment failure and that Api m 10 was underrepresented in 3 of 5 therapeutic HBV preparations while it was present in both of the crude HBV preparations analyzed. Besides, significant induction of sIgG4 was only observed in patients treated with the HBV that contained detectable amounts of Api m 10. The authors speculate that processing/purification of the crude HBV during the manufacturing process may lead to the loss of Api m 10 immunoreactivity.

The aim of the study was to characterize the allergens in the venom preparation marketed by Anallergo (Florence, Italy) for diagnosis and immunotherapy and particularly to demonstrate that Api m10 was present both in the crude venom (Entomon) and therapeutic Anallergo venom.

**Methods:** Venom was digested with trypsin. Aliquots of venom was analyzed by UHPLC-ESI–MS/MS (ThermoFisher Scientific)

**Results:** Shotgun proteomics analysis demonstrated that Anallergo venom contained major allergenes Api m1 Api m2 e Api m4; besides the analysis revealed the presence of Api 3 Api m5 e Api m10 too. The same results was obtained by the study of crude venom (Entomon) by which therapeutic preparation Anallergo is performed.

**Conclusions:** The study demonstrated that therapeutic preparation bee venom Anallergo contain all the relevant allergenes and in particularly Api m10

#### P19 Bet V1-like superfamily proteins in apple

##### Valentina Cova, Thomas Letschka

###### Laimburg Research Centre, Ora (BZ), Italy

**Correspondence:** Valentina Cova - valentina.cova@laimburg.it

*Clinical & Translational Allergy* (*CTA*) 2018, **8(Suppl 1):**P19

**Background:** Bet v 1 is the major birch pollen allergene and it is recognised by IgE antibodies from about 95% of birch-allergic patients.

The most striking feature of the three-dimensional structure of Bet v 1 is the presence of a large hydrophobic cavity, which is open to the exterior and functions as a ligand binding site. The surprising similarity of the structure of the Bet v 1-related allergens revealed the existence of a large superfamily of mostly lipid-binding proteins with a common fold.

**Methods:** Fifty-seven putative proteins belonging to the Bet v1-like superfamily have been downloaded from the apple genome at the Genome Database for Rosaceae website.

Sequences have been aligned and a phylogenetic tree has been constructed defining three main subfamilies: PR-10 (Mal d 1-like), PhBPs and MLR/RRPs.

**Results:** Mal d 1 is the founding member of the major apple allergen family and belongs to the PR-10 proteins (Pathogenesis related proteins). Up to now 31 members of this multigene family have been isolated and the three-dimensional structure of Mal d 1.0101 isoform has been recently solved. Mal d 1 and Bet v 1 proteins share 64.5% amino acid sequence identity and have common IgE epitopes that lead to allergic cross-sensitization.

Phytohormone Binding Proteins (PhBPs) have been firstly named as CSBP (cytokinin-specific binding proteins) because they were considered as strong cytokinin binders. However, recently the crystal structure of these proteins have been solved bound to another important phytohormone, that is gibberellic acid.

Act d 11 is a protein found abundantly in ripe green and yellow-fleshed kiwifruit. Ten percent of kiwifruit allergic individuals bear IgE that recognizes Act d 11. This protein belongs to the Major Latex Protein/Ripening Related Protein (MLP/RRPs) family and is the first protein of this family identified as an allergen. Act d 11 is immunologically related to Bet v 1-like allergens. MLP/RRP and PR-10 families both belong to the Bet v 1 superfamily, but the sequence identity between the members of the two protein groups is rather low (< 25%). However, it was shown that despite the low sequence identity, Act d 11 is able to inhibit, at least partially, binding of IgE to Bet v 1 and Mal d 1, suggesting that these allergens share some IgE epitopes.

**Conclusions:** The present study explores not only the proteins belonging to the Mal d 1 family, but enlarges its focus to the Bet v 1-like superfamily in the perspective to identify new putative unknown allergens.

#### P20 Development of sensitive and specific ELISA assays for the investigation of the transfer of Ara H 2 and Ara H 6 in human breast milk

##### Frauke Schocker^1^, Alexandra Scharf^1^, Skadi Kull^1^, Uta Jappe^2^

###### ^1^Division of Clinical and Molecular Allergology, Research Center Borstel, Borstel, Germany; ^2^Division of Clinical and Molecular Allergology, Research Center Borstel; Interdisciplinary Allergy Outpatient Clinic, Department of Internal Medicine, University of Lübeck, Borstel; Lübeck, Germany

**Correspondence:** Uta Jappe - ujappe@fz-borstel.de

*Clinical & Translational Allergy* (*CTA*) 2018, **8(Suppl 1):**P20

**Background:** Peanut allergy belongs to one of the most severe food allergies in the westernized counties and has emerged as a problem in the German speaking counties, too. Whether breast feeding induces tolerance development or on the contrary, leads to sensitization to peanuts is still under discussion. In this study, we developed sensitive and specific diagnostic tools for the investigation of two clinically relevant peanut allergens, Ara h 2 and Ara h 6, in human breast milk in our German breast milk study.

**Methods:** We recruited 40 lactating women without a history of peanut allergy, each consuming 100 g of dry roasted peanuts after which breast milk samples were retrieved at different time points. Two ELISA systems were developed and validated for the quantification of Ara h 2 and Ara h 6 in the low ng/mL range.

**Results:** The Ara h 2 ELISA revealed a limit of detection (LOD) of 1.3 ng Ara h 2/mL breast milk and a quantification range of 2.3–250 ng/mL. The Ara h 6 ELISA showed an LOD of 0.7 ng/mL and a quantification range of 1.1–14.4 ng/mL. No relevant cross-reactivities against potentially relevant cross-reactive legume, tree nut and seed extracts were noted. By means of these assays, Ara h 2 could be measured in 14/40 (35%) lactating women in concentrations between 2.3 and 184 ng/mL breast milk and Ara h 6 was detected in 9/40 (22.5%) of the participants between 1.1 and 9.7 ng/mL and one highly positive sample with 79 ng/mL. Notably, Ara h 2 and Ara h 6 were transferred at the same time courses of appearance after ingestion, but Ara h 6 in lower concentrations than Ara h 2.

**Conclusions:** The Ara h 2 and Ara h 6 ELISA were developed as sensitive and specific diagnostic tools for the assessment of the allergen concentration in human breast milk. Evidently, Ara h 2 and Ara h 6 are transferred at the same time points after peanut exposition, however a difference in concentration was observed. By this means investigations on the allergens’ sensitizing or tolerogenic properties in human breast milk become accessible on the molecular level.

#### P21 Functional characterization of TRP channels in bone marrow-derived dendritic cells

##### Robbe Naert, Alejandro López-Requena, Sven Seys, Karel Talavera, Yeranddy Aguiar Alpizar

###### K.U. Leuven, Leuven, Belgium

**Correspondence:** Robbe Naert - robbe.naert@kuleuven.vib.be

*Clinical & Translational Allergy* (*CTA*) 2018, **8(Suppl 1):**P21

**Background:** Several dendritic cell (DCs) stages, such as differentiation, maturation and migration, are strongly modulated by changes in intracellular Ca2+ concentration. These changes are promoted by activation of Ca2+ -release activated channels, ryanodine and purinergic receptors that are activated downstream of signalling pathways initiated by membrane receptors (G-protein coupled receptors) or by damage-associated signals (ATP). Recently, transient receptor potential (TRP) channels have been described to be expressed in immune cells, including DCs. However, the roles of these cation-permeable channels in these cells remain obscure. In this study, we determined the expression of TRP channels in mouse bone marrow-derived dendritic cells (BMDCs).

**Methods:** BMDCs were generated from WT and Trpv4 KO mice and were used to identify TRP channel expression via qPCR. We assessed the functional expression of TRPV2 and TRPV4 using calcium imaging. An immunofluorescent staining was performed to confirm the presence of TRPV4 in the plasma membrane of DCs. We used flow cytometry to check the purity of the BMDC cell population.

**Results:** We found that TRPM2, TRPM4, TRPM7, TRPV2 and TRPV4 are expressed in the CD11c+ BMDCs, and confirmed the functional expression of TRPV2 and TRPV4. Furthermore, we show that TRPV4 is dispensable for the differentiation and the LPS-induced maturation of CD11c+ BMDCs, and that activation of this channel induces an immediate transient expression of CCL-11, a chemotactic protein for eosinophils.

**Conclusions:** Since TRPV4 is a polymodal sensor, activated by mechanical and thermal stimuli, our findings suggest that TRPV4 activity may condition the activation state of DCs, as well as regulate the infiltration of eosinophils in the absence of pathogenic insults.

#### P22 Whole transcriptome association study identifies differentially expressed genes for allergic rhinitis shared between Singapore and Swedish cohorts

##### Anand Kumar Andiappan^1^, Bernett Lee^2^, Simon Kebede Merid^3^, Josephine Lum^1^, Shihui Foo^1^, Geraldine Koh^1^, John Edward Conolly^4^, Chiea Chuen Khor^5^, Jianjun Liu^5^, Michael Poidinger^1^, Francesca Zolezzi^1^, DeYun Wang^6^, Erik Melén^3^, Olaf Rotzschke^1^

###### ^1^Singapore Immunology Network (SIgN), Agency for Science, Technology and Research (A*STAR), Singapore, Singapore; ^2^Singapore Immunology Network (SIgN), Agency for Science, Technology and Research (A*STAR), Singapore, Singapore, Singapore; ^3^Institute of Environmental Medicine, Karolinska Institutet, Stockholm, Sweden, Stockholm, Sweden; ^4^Institute for Molecular and Cell Biology (IMCB), Agency for Science, Technology and Research (A*STAR), Singapore, Singapore; ^5^Genome Institute of Singapore (GIS), Agency for Science, Technology and Research (A*STAR), Singapore, Singapore; ^6^National University of Singapore (NUS), National University Health System (NUHS), Singapore, Singapore

**Correspondence:** Anand Kumar Andiappan - anand_andiappan@immunol.a-star.edu.sg

*Clinical & Translational Allergy* (*CTA*) 2018, **8(Suppl 1):**P22

**Background:** Allergic rhinitis (AR) is common chronic inflammatory disorder of the upper airway. An estimated 500 million people would have AR in less than a decade and this high prevalence is expected both in children and young adults. Recently we have reported high dust mite sensitisation prevalence in Singapore with nearly 40% population have recorded symptoms of AR. Although some genetic candidates have been associated to AR risk, these only account for a small amount of heritability. Here we investigate the transcriptional candidates differentially expressed in AR subjects.

**Methods:** Whole transcriptome analysis for the Singapore cohort was performed in 112 AR cases and 113 controls using the Illumina Human HT12 array. Replication analyses of significant candidates from Singapore was performed in the BAMSE cohort of 78 AR cases and 125 controls with expression data from the Affymetrix HTA 2.0 Genechips. Further enrichment analysis was performed using Ingenuity Pathway Analysis (IPA) and Gene ontology softwares.

**Results:** (Table 1) Discovery cohort: The Singapore cohort had 23 unique probes significant at a genome-wide FDR threshold of 0.05. Significant candidates had some previously known AR candidates such as CLC, IL5RA, ALOX15 and SIGLEC8. IPA analysis of nominally significant revealed components of both innate and adaptive immunity in the significant in AR pathogenesis. Some key functions included recruitment, infiltration and response of eosinophils, tolerisation of mast cells and activation of basophils; and activation of Th2 cells and myeloid cells, leukocyte migration.

**Table 1:** Results:

Gene	Probe	*P* value	P. adj	Avg_Expr	Fold change	P. eosadj
SIGLEC8	ILMN_1730295	2.22E−07	4.50E−03	11.92	1.49	2.25E−04
IL5RA	ILMN_2327812	4.01E−07	4.50E−03	13.46	1.39	4.04E−05
ALOX15	ILMN_1729320	4.59E−07	4.50E−03	8.95	1.44	3.49E−04
OLIG2	ILMN_1727567	1.16E−06	8.53E−03	9.61	1.53	5.45E−04
SMPD3	ILMN_1802316	1.74E−06	9.67E−03	9.89	1.28	4.53E−03
C21orf130	ILMN_3243121	1.98E−06	9.67E−03	7.68	1.34	1.23E−03
C10orf33	ILMN_1684497	2.72E−06	1.08E−02	12.48	1.15	7.11E−05
HES1	ILMN_1710284	2.95E−06	1.08E−02	10.84	1.28	2.42E−03
CCL23	ILMN_1686109	7.57E−06	2.21E−02	11.95	1.52	1.33E−02
TFF3	ILMN_1811387	8.55E−06	2.21E−02	10.03	1.53	1.68E−02
RASL11B	ILMN_2148469	8.70E−06	2.21E−02	8.39	0.82	2.88E−02
HRASLS5	ILMN_1674349	9.05E−06	2.21E−02	8.07	1.29	5.51E−03
ALOX15	ILMN_2403534	9.78E−06	2.21E−02	12.53	1.33	8.25E−04
EMR4	ILMN_2411998	1.16E−05	2.39E−02	11.97	1.33	5.75E−04
CLC	ILMN_1654875	1.29E−05	2.39E−02	14.8	1.14	1.06E−03
CCL23	ILMN_1764030	1.30E−05	2.39E−02	9.95	1.60	2.24E−03
P2RY2	ILMN_2297854	1.47E−05	2.54E−02	9.24	1.24	2.58E−02
THBS4	ILMN_1736078	2.43E−05	3.96E−02	8.93	1.31	2.64E−04
PRSS33	ILMN_1736831	2.59E−05	4.01E−02	10.22	1.51	1.05E−02
SLC29A1	ILMN_2338963	3.30E−05	4.63E−02	13.29	1.21	3.32E−02
IL17RB	ILMN_1767523	3.49E−05	4.63E−02	8.92	1.27	1.90E−02
GPR44	ILMN_1703326	3.57E−05	4.63E−02	11.96	1.32	1.78E−02
SLC29A1	ILMN_1723971	3.63E−05	4.63E−02	12.47	1.33	1.19E−02

Replication cohort: We then tested genes from these 23 probes (FDR < 0.05) for association in the Swedish BAMSE cohort. Of the 16 genes tested 12 genes had a significant validation FDR *p* value < 0.05.

Cell percentages: Given that some of these genes were eosinophil relevant, we then corrected the association analysis for eosinophil percentages. Interestingly, in the Singapore cohort all the genome-wide significant candidates were significant after correcting for eosinophil percentages. However in the BAMSE cohort, 6 out of the 12 genes were significant with eosinophil count added as a covariate.

**Conclusions:** This study has revealed a significant number of AR candidates shared between Singapore and Swedish cohorts. The pathways thus identified can be potentially targeted for disease treatment and management. It would be important to identify if genetic polymorphisms affect these expression candidates and evaluate if identified genes can also.

### Poster Discussion Session I - Topic 3: Novel allergen molecules

#### P23 Identification and molecular characterization of allergenic NsLTP from durum wheat (*Triticum durum*)

##### Andrea Wangorsch^1^, Héla Safi^2^, Jonas Lidholm^3^, Faiçal Brini^2^, Jelena Spiric^4^, Hans-Peter Rihs^5^, Stefan Vieths^1^, Alicia Armentia^6^, Laura Farioli^7^, Araceli Diaz-Perales^8^, Elide Anna Pastorello^7^, Stephan Scheurer^1^

###### ^1^Molecular Allergology, Paul-Ehrlich-Institut, Federal Institute for Vaccines and Biomedicines, Langen, Germany; ^2^Laboratory of Biotechnology and Plant Improvement, Centre of Biotechnology of Sfax, Sfax, Tunisia; ^3^Thermo Fisher Scientific, Uppsala, Sweden; ^4^Division Allergology, Paul-Ehrlich-Institut, Federal Institute for Vaccines and Biomedicines, Langen, Germany; ^5^Institut für Prävention und Arbeitsmedizin der Deutschen Gesetzlichen Unfallversicherung, Institut der Ruhr-Universität Bochum (IPA), Molekulare Genetik, Bochum, Germany; ^6^Hospital Universitario Río Hortega, Valladolid, Spain; ^7^Dipartimento Medico Polispecialistico & Università degli Studi di Milano, ASST Grande Ospedale Metropolitano Niguarda, Milano, Italy; ^8^Departamento de Biotecnología-Biología Vegetal, E.T.S. Ingenieros Agrónomos/Centro de Biotecnología y Genómica de Plantas (CBGP, UPM-INIA) Universidad Politécnica de Madrid, Madrid, Spain

**Correspondence:** Andrea Wangorsch - andrea.wangorsch@pei.de

*Clinical & Translational Allergy* (*CTA*) 2018, **8(Suppl 1):**P23

**Background:** Common wheat (*Triticum aestivum*, bread) and durum wheat (*Triticum durum*, semolina, pasta, couscous), can cause food allergy/WDEIA or baker’s asthma. Over-expression of non-specific lipid transfer proteins (nsLTPs) in *T. durum* is considered to increase its resistance against pathogens.

Aim of the study was the recombinant expression, molecular characterization and allergenicity assessment of nsLTP from *T. durum* (Tri d LTP) in comparison to Tri a 14 (*T. aestivum*) and Pru p 3 (peach).

**Methods:** Recombinant Tri d LTP was purified via two step chromatography. Secondary structure and purity were assessed by CD spectroscopy and SDS-PAGE, respectively. 32 wheat allergic patients were enrolled: 20 Spanish patients with baker’s asthma and positive bronchial challenge tests, and 12 Italian patients with wheat food allergy/WDEIA confirmed by positive DBPCFC or OFC. All patients were preselected by IgE sensitization to Tri a 14, either by SPT (Spanish) or ImmunoCAP testing (Italian). Moreover, 7 Italian Tri a 14 sensitized but asymptomatic subjects were included. Specific IgE values to wheat, Tri d LTP, Tri a 14 and Pru p 3 were determined by ImmunoCAP testing. Accumulation of natural Tri d LTP in *T. durum* and its cross-reactivity with Tri a 14 and Pru p 3 were analyzed by IgE immunoblot or ELISA inhibition experiments.

**Results:** The two wheat nsLTPs were found to share only 48% amino acid identity with each other, and 52% (Tri d LTP) and 46% (Tri a 14) with Pru p 3. Recombinant Tri d LTP, rTri a 14 and nPru p 3 displayed similar secondary structures. Among 25 Tri a 14 CAP positive sera, 92% were reactive to wheat extract, 88% to Tri d LTP and 80% to Pru p 3. The correlation between Tri a 14 and Tri d LTP specific IgE levels was r = 0.78 (baker’s asthma) and r = 0.93 (food allergy/WDEIA). The subgroup of food allergic/WDEIA patients showed highest specific IgE values to Tri d LTP (8.8 kUA/L) and Pru p 3 (5.8 kUA/L), whereas nsLTP-specific IgE values were low in patients with baker’s asthma. Tri d LTP displayed higher IgE cross-reactivity with Pru p 3 than Tri a 14, whereas IgE cross-reactivity between the two wheat LTPs varied between individual patients. IgE competition assays provided an indication of the abundance of Tri d LTP in *T. durum* wheat flour extract.

**Conclusions:** Sensitization to nsLTP appears more important in wheat food allergy than in baker´s asthma. The first time an allergen in *T. durum* was identified as nsLTP. Sensitization to Tri d LTP is closely associated to Pru p 3-mediated food allergy.

#### P24 Update of the AllergenOnline.org database for risk assessment of new proteins used in foods

##### Richard E Goodman^1^, Ronald Van Ree^2^, Barbara Bohle^3^, Fatima Ferreira^4^, Motohiro Ebisawa^5^, Joerg Kleine-Tebbe^6^, Afua Okobea Tetteh^7^, Plaimein Amnuaycheewa^8^, Frits Koning^9^, J. L. Baumert^1^, S. L. Taylor^1^

###### ^1^Food Allergy Research and Resource Program, Department of Food Science and Technology, University of Nebraska-Lincoln, Lincoln, NE, USA; ^2^Department of Experimental Immunology, Academic Medical Center, Amsterdam, the Netherlands; ^3^Institute of Pathophysiology and Allergy Research, Medical University of Vienna, Vienna, Austria; ^4^Department of Molecular Biology, University of Salzburg, Salzburg, Austria; ^5^Department of Allergy, Clinical Research Center for Allergy and Rheumatology, Sagamihara National Hospital, Sagamihara, Kanagawa, Japan; ^6^Allergy & Asthma Center Westend, Outpatient Clinic Ackermann, Hanf & Kleine-Tebbe, Berlin, Germany; ^7^Sean N. Parker Center for Allergy and Asthma Research, Stanford University School of Medicine, Stanford University, Palo Alto, CA, USA; ^8^Department of Agro-Industrial, Food and Environmental Technology, King Mongkut’s University of Technology, Bangkok, Thailand; ^9^Leiden University Medical Center, Leiden, the Netherlands

**Correspondence:** Richard E Goodman - rgoodman2@unl.edu

*Clinical & Translational Allergy* (*CTA*) 2018, **8(Suppl 1):**P24

**Background:** Proteins introduced in foods by genetic engineering are evaluated for potential risks of eliciting food allergy or celiac disease (CODEX, 2003). Primary risks occur by the transfer of an allergen or nearly identical protein that can cause IgE-mediated reactions in allergic consumers. Proteins from wheat relatives (Pooideae), should be evaluated for the possibility of eliciting celiac disease (CD). AllergenOnline.org was developed in 2005 and is updated annually to include proteins causing IgE mediated reactions and includes search routines listed by CODEX. The CD database was added in 2012 with evaluation by exact peptide match and FASTA searches.

**Methods:** Guidelines were developed for reviewing and classifying proteins as “allergens”, “putative allergens” or those with “insufficient evidence” of causing IgE mediated allergic reactions in humans. Airway, contact, venom, salivary and food allergens are included. Criteria were developed to define allergic subjects, allergen sources, protein characteristics, sequences, allergenic activity and IgE binding. Candidate allergens and peer-reviewed publications are identified from the NCBI Protein and PubMed databases. Data evaluations and decisions are achieved annually. Browse and FASTA searches are public, anonymous and not monitored. Peptides and proteins for the CD database represent > 1016 peptides and 68 proteins, from literature review. Most peptides bind HLA-DQ2, or DQ8 and stimulate CD specific CD4+ T cells. A few are toxic, not immunogenic.

**Results:** Version 17 of AOL includes 2035 allergens and putative allergens from 808 taxonomic protein groups (references listed). Version 18 will have a number of new entries. Proteins matching an allergen above CODEX criteria should be tested by serum IgE binding tests. A beta-version of the CD database has a beta version with 1030 peptides, including those recommended by the European Food Safety Authority. Many of those are HLA-specific 9 amino acid peptides. But, T cell reactivity requires more specificity so longer peptides and proteins are included. Matches indicate a probable need for CD-specific T cell assays if the matched protein would be present in non-wheat related foods. The database updates will occur in January 2018.

**Conclusions:** Publications and sequence entries claiming to identify new allergens are common. AllergenOnline provides a peer review system to improve safety evaluations of dietary proteins for risks of allergenicity or CD.

#### P25 Identification of a major allergen from macadamia nut

##### Stefanie Rohwer, Yvonne Denno, Alf Weimann, Winfried Stöcker, Waltraud Suer

###### EUROIMMUN AG, Lübeck, Germany

**Correspondence:** Stefanie Rohwer - s.rohwer@euroimmun.de

*Clinical & Translational Allergy* (*CTA*) 2018, **8(Suppl 1):**P25

**Background:** Macadamia nuts (*Macadamia integrifolia*) are predominantly grown and consumed in Oceania, although they become more and more part of the diet in Europe primarily in bakery products and snacks. With rising consumption the number of cases of macadamia nut allergies increases. Today, they are commonly diagnosed by the detection of serum IgE antibodies against the nut extract. However, for a reliable diagnosis and for the differentiation between allergies to macadamia nuts and other tree nuts and seeds, it is necessary to analyse the subfractions of macadamia nut and to determine the immunoreactivity against defined partial allergens.

The aim of this study was the identification of components of macadamia nut associated with allergy.

**Methods:** Proteins from macadamia nut extract (MNE) were separated by 2D gel electrophoresis and subsequently blotted onto nitrocellulose membrane. Blots were incubated with sera of macadamia nut sensitized patients and non-sensitized controls. Specific immunoreactive spots were analysed by means of MALDI-TOF. Proteins were identified, cloned, expressed in *E. coli*, purified and coated on membranes to produce line blots. IgE reactivity was determined in the serum of patients and controls.

**Results:** 2D Western blots of MNE with sera from macadamia nut sensitized patients showed major spots at a molecular mass around 53–67 kDa and in a pH interval of around 6–9. These spots corresponded to vicilin-like antimicrobial peptides of Macadamia integrifolia (Mac i-VLAP), which were apparently present in different modifications. 11 of 16 sera of macadamia nut sensitized patients reacted with recombinant Mac i-VLAP expressed in *E. coli*, but none of the controls. The cross reactivity of 24 rMac i-VLAP positive sera to 7 vicilins of other nuts and seeds was analysed in line blots. It was shown that 83% of the sera cross reacted with vicilins from peanut (rAra h 1) and cashew nut (rAna o 1). Furthermore, cross reactivity was observed to vicilins from pecan nut (rCar i 2, 67%), pistachio (rPis v 3, 63%), walnut (rJug r 2, 58%), sesame (rSes i 3, 54%) and hazelnut (rCor a 11, 46%).

**Conclusions:** Mac i-VLAP was identified as a major allergen in macadamia nut. It is useful for detailed partial allergen diagnostics in allergologic laboratories. For a comprehensive diagnosis of nut allergies IgE detection using profiles including different nut allergens especially storage proteins like vicilins is crucial.

#### P26 Identification and characterization of IgE reactive low molecular weight peanut proteins

##### Skadi Kull^1^, Christian Schwager^1^, Marisa Böttger^1^, Uta Jappe^1^

###### ^1^Research Center Borstel, Borstel, Germany

**Correspondence:** Christian Schwager - cschwager@fz-borstel.de

*Clinical & Translational Allergy* (*CTA*) 2018, **8(Suppl 1):**P26

**Background:** Peanut allergy is the most common cause of life-threatening anaphylaxis in adolescents and children in German speaking countries. Recently, we identified two novel peanut allergens, the defensins, denominated Ara h 12 and Ara h 13. Interestingly, in the course of purification we encountered a further IgE-reactive protein in the low molecular weight (LMW) range. In order to investigate the allergenic risk and improve diagnostic test systems, allergens that lead to clinical reactions need to be identified and characterized. Therefore, our aim was to isolate and characterize this potential novel allergen. Moreover, we wanted to compare the impact of thermal processing of this molecule with known LMW peanut allergens (Ara h 12/Ara h 13) on IgE reactivity as food processing (e.g. roasting) has been shown to increase the allergenicity of diverse peanut allergens.

**Methods:** LMW peanut proteins of raw and in-shell roasted peanuts were isolated by lipophilic extraction and subsequent chromatographic separation techniques. Isolated proteins were identified by mass spectrometry and N-terminal sequencing. Sera of peanut-allergic patients with severe allergic symptoms, sensitized but peanut-tolerant patients and non-allergic individuals were screened by immunoblot analysis for IgE binding to these molecules. Additionally, the ability of the isolated proteins to trigger allergic reactions was assessed by basophil activation test.

**Results:** In the course of Ara h 12/Ara h 13 purification, we encountered a novel LMW IgE reactive peanut protein which was able to stimulate basophils of peanut-allergic individuals in vitro. Mass spectrometric analysis and N-terminal sequencing revealed that the IgE reactive protein is a third novel peanut defensin with a homology of 32% to Ara h 12, 39% to Ara h 13.0101 and 41% to Ara h 13.0102, respectively. The majority of peanut-allergic patients sensitized to defensins displayed more severe allergic symptoms. Defensins from in-shell roasted peanuts showed a higher IgE binding capacity in western blot analysis and led to an increased basophil activation compared to peanut defensins from raw peanuts.

**Conclusions:** Roasting enhances the IgE binding of the novel identified peanut defensin, as well as of Ara h 12 and Ara h 13. Furthermore, our data suggests that IgE binding to peanut defensins correlates with the severity of allergic symptoms.

#### P27 IgE and allergenic activity against α-Gal containing proteins in the ticks ixodes *Ricinus* and *Amblyomma americanum*

##### Danijela Apostolovic^1^, Scott Commins^2^, Jelena Mihailovic^3^, Maria Starkhammar^4^, Tanja Cirkovic Velickovic^3^, Thomas A. Platts-Mills^5^, Carl Hamsten^1^, Marianne Van Hage^1^

###### ^1^Immunology and Allergy Unit, Department of Medicine Solna, Karolinska Institutet, Stockholm, Sweden; ^2^University of North Carolina School of Medicine, Chapel Hill, NC, USA; ^3^Center of Excellence for Molecular Food Sciences, Faculty of Chemistry, University of Belgrade, Belgrade, Serbia; ^4^Department of Internal Medicine, Södersjukhuset, Stockholm, Sweden; ^5^Asthma and Allergic Diseases Center, University of Virginia Health System, Charlottesville, VA, USA

**Correspondence:** Danijela Apostolovic - danijela.apostolovic@ki.se

*Clinical & Translational Allergy* (*CTA*) 2018, **8(Suppl 1):**P27

**Background:** The mammalian carbohydrate galactose-α-1,3-galactose (α-Gal) has shown to be the cause of a novel form of severe food allergy, red meat allergy. Today there is evidence for tick bites as the route of sensitization for the IgE response to α-Gal. The aim of this study was to compare the IgE reactivity against α-Gal in the ticks *Ixodes ricinus* (*I. ricinus*) and *Amblyomma americanum* (*A. americanum*), between Swedish and US red meat allergic patients. In addition, the allergenic activity was investigated by basophil activation test.

**Methods:** Protein extracts from *I. ricinus* (adult and larvae forms) and *A. americanum* (larvae form) ticks were coupled to streptavidin ImmunoCAP and IgE reactivity was measured among 25 Swedish and 18 US red meat allergic patients. IgE binding was analysed on 1D immunoblot. Allergenic activity against HSA-α-Gal, tick extracts and deglycosylated tick extract was tested by basophil activation assay on 6 Swedish red meat allergic patients.

**Results:** Our data showed that 96% of Swedish red meat allergic patients have an IgE response to extracts from *I. ricinus* and 42% from *A. americanum*. Among the US patients 89% had IgE reactivity to extracts from *I. ricinus* in comparison to 30% from *A. americanum*. There was moderate to high correlation between the IgE levels to α-Gal and extracts from adult (ρ = 0.54 for Swedish sera and 0.87 for USA sera) and larvae from *I. ricinus* (ρ = 0.54 for Swedish sera and ρ = 0.88 for US sera). There was no correlation between IgE levels to α-Gal and *A. americanum* larvae extract. In 1D-immunoblotting a wide-range of protein bands (25–150 kDa) were identified by the Swedish and American serum pools. The *A. americanum* larvae showed to have less IgE-binding protein bands than *I. ricinus*. The presence of α-Gal in both tick species was further supported by IgE inhibition using thyroglobulin. All patients tested showed allergenic activity against extract from adult *I. ricinus* in the basophil activation assay. Deglycosylation of the *I. ricinus* extract reduced the allergenic activity.

**Conclusions:** The results support the strong relationship with tick bites for the production of IgE to α-Gal. Furthermore, that tick proteins have allergenic activity. However, the IgE reactivity to α-Gal containing tick extract seem to vary between species and stadium of the tick life cycle, suggesting that ticks may acquire the α-Gal epitope from their mammalian hosts.

#### P28 Pru P X is a new allergen of peach tree pollen involved in sensitization

##### Maria Luisa Somoza-Alvarez

###### Hospital Infanta Leonor, Madrid, Spain

**Correspondence:** Maria Luisa Somoza-Alvarez - mblancago@gmail.com

*Clinical & Translational Allergy* (*CTA*) 2018, **8(Suppl 1):**P28

**Background:** Peach tree pollen has ben identified as relevant in areas of peach tree cultivar. After olive and grass, it is the third one inducing sensitisation in these areas. Our aim was to study if peach tree pollen contain other allergens that can induce sensitisation in addition to Pru p 3.

**Methods:** Skin tests with peach pollen extracts were made in subjects with seasonal symptoms during the period of production of this pollen in an area of high exposure. Sera from positive skin tests cases were obtained and SDS-PAGE and immunoblotting analysis was made.

**Results:** Using pool of sera of mono-sensitized cases negative to Pru p 3 and other pollens several bands were identified that corresponded to 45, 25 and 15 kD. We named the 15D band as Pru p X. This protein and Pru p 3 in 110 cases skin test positive to peach pollen. The 40% were prick positive to Pru p X and the 35% to Pru p 3. The 12% were positive to both and in the remaining cases with skin test positive to peach pollen both were negative.

**Conclusions:** Peach pollen has several allergens that can be involved in the induction of sensitisation and allergy in highly exposed populations. From these we identify the Pru p X that has not been previously recognized. Because subjects were also positive to Pru p 3, the respiratory tract can be a pathway of sensitisation to this pan-allergen. The clinical relevance of these findings is under evaluation.

#### P29 Class III chitinase is a new IgE binding protein identified in pomegranate

##### Lisa Tuppo^1^, Ivana Giangrieco^1^, Claudia Alessandri^2^, Maurizio Tamburrini^1^, Chiara Rafaiani^2^, Michela Ciancamerla^2^, Adriano Mari^2^, Maria Antonietta Ciardiello^1^

###### ^1^Istituto di Bioscienze e Biorisorse, Consiglio Nazionale delle Ricerche, Naples, Italy; ^2^Centri Associati di Allergologia Molecolare - CAAM, Rome, Italy

**Correspondence:** Adriano Mari - adriano.mari@caam-allergy.com

*Clinical & Translational Allergy* (*CTA*) 2018, **8(Suppl 1):**P29

**Background:** Pomegranate, *Punica granatum*, is a fruit whose consumption is growing as it is considered a health-promoting food. Mild to very severe allergic reactions to this fruit are also increasingly reported. Class III chitinase is one of the major protein components found in the extracts from pomegranate pulp, but its potential allergenicity has not been investigated so far.

**Methods:** Aim of this study was the isolation of pomegranate class III chitinase and the investigation of its allergenicity. The protein was isolated from pomegranate pulp extract by RP-HPLC, identified by direct protein sequencing, purified by conventional chromatographic separations and the capacity to bind specific IgE in the sera of allergic subjects was investigated using the FABER^®^ test bearing five pomegranate’s preparations in total: Pun g (seed extract), Pun g 1 (9k-LTP), Pun g 5 (Hevein like protein), Pun g 7 (Pommaclein), Pun g 14 (class III Chitinase).

**Results:** The pomegranate class III chitinase (UniProtKB accession number G1UH28) is a 273-residue protein migrating on SDS-PAGE as a band of about 30 kDa. The homology search performed in the Allergome database shows a high similarity with the latex hevamine (Hev b 14) and with the raspberry homolog (Rub i Chitinase), sharing 69 and 62% sequence identity, respectively. A lower structural similarity has been recorded with other IgE binding class III chitinases, such as the one from Chinese-date (Ziz m 1) and from coffee (Cof a 1), sharing 42 and 38% identity, respectively. The purified Pun g 14 was spotted on the FABER^®^ biochip and the results obtained after testing a large population of allergic subjects were analyzed in comparison with those obtained for the other pomegranate allergenic preparations available on the FABER^®^ test. Out of 4537 tested patients 266 (6%) turned out to be sensitized to at least one of the 5 pomegranate allergenic preparations present on FABER^®^, with the following prevalence calculated out of the 266 patients: Pun g seed extract and Pun g 1 54%, Pun g 14 23%, Pun g 7 13% and Pun g 5 6%.

**Conclusions:** A new IgE binding protein, the class III chitinase, Pun g 14, has been identified in pomegranate. It displays high structural similarity with the homologous allergens from latex and raspberry. Pun g 14 can contribute to improve the allergy diagnosis to pomegranate and, in general, to plant allergenic sources.

#### P30 Evaluation and characterization of Ory c 3, a major rabbit allergen

##### Stephanie Kler^1^, Delphine Lentz^1^, Dominique Revets^1^, Christiane Lehners^2^, Catherine Charpentier^3^, Martine Morisset^3^, Markus Ollert^1^, François Hentges^2^, Christiane Hilger^1^

###### ^1^Department of Infection and Immunity, Luxembourg Institute of Health (LIH), Esch-sur-Alzette, Luxembourg, Luxembourg; ^2^Immunology-Allergology Unit, Centre Hospitalier, Luxembourg, Luxembourg; ^3^Department of Pneumology, Centre Hospitalier, Luxembourg, Luxembourg

**Correspondence:** Stephanie Kler - stephanie.kler@lih.lu

*Clinical & Translational Allergy* (*CTA*) 2018, **8(Suppl 1):**P30

**Background:** Ory c 3 is a major rabbit allergen. It is a heterodimer composed of two peptide chains, lypophillins called CL2 and AL. Ory c 3 belongs to the secretoglobin family and it has high structural identity with Fel d 1, the major cat allergen. Objective: To produce Ory c 3 as recombinant protein, to compare it to the natural allergen and to set up a detection assay.

**Methods:** cDNAs corresponding to Ory c 3.A.0101 (CL2) and Ory c 3.B.0101 (AL) were isolated from rabbit salivary gland by RACE PCR. Both cDNAs were cloned as head-to-tail construct with C-terminal His-tag. Recombinant Ory c 3 (rOry c 3) was expressed in *E. coli* and purified by affinity and ion exchange chromatography. Native Ory c 3 (nOry c 3) was purified from rabbit fur by gel filtration and ion exchange. Identity was assessed by by mass spectrometry. Secondary structure analysis was performed using circular dichroism. IgE-binding of rOry c 3 and nOry c 3 was analysed by ELISA using sera from 36 rabbit-allergic patients. Polyclonal anti-sera to rOry c 3 were raised in guinea-pigs and an Ory c 3 detection assay was established.

**Results:** rOry c 3 was expressed as head-to-tail fusion protein. The recombinant protein showed a folding which was similar to nOry c 3. Thermal stability was very high and both proteins readily folded back to their initial structures. Mass spectrometry of purified nOry c 3 confirmed that the heterodimer is composed exclusively of CL and AL2. 81% of the rabbit-allergic patients were sensitized to nOry c 3 and IgE-binding to rOry c 3 and nOry c 3 was very similar (r = 0.9689). Ory c 3 could be detected in rabbit urine and dander. The allergen was also confirmed to be present in the New Zealand White rabbit, dwarf rabbit and two breeds raised for meat.

**Conclusions:** The expression of rOry c 3 as fusion protein of two monomers yielded a recombinant protein of similar structure, stability and IgE-binding as the natural allergen. Ory c 3 is a specific marker of rabbit allergy and a valuable diagnostic tool for determining a primary sensitization.

#### P31 Characterization of allergenic parvalbumins from angler fish (*Lophius piscatorius*)

##### Thorsten Graf^1^, Andrea Steinbauer^2^, Françoise Codreanu-Morel^3^, Tanja Scheuermann^1^, Dominique Revets^1^, François Hentges^1^, Walter Keller^4^, Markus Ollert^1^, Martine Morisset^3^, Ines Swoboda^2^, Annette Kuehn^1^

###### ^1^Department of Infection and Immunity, Luxembourg Institute of Health, Esch-Sur-Alzette, Luxembourg; ^2^Molecular Biotechnology Section, FH Campus Wien, University of Applied Sciences, Campus Vienna Biocenter, Vienna, Austria; ^3^National Unit of Immunology and Allergology, Centre Hospitalier de Luxembourg, Luxembourg, Luxembourg; ^4^Institute of Molecular Biosciences, University of Graz, Graz, Austria

**Correspondence:** Thorsten Graf - thorsten.graf@lih.lu

*Clinical & Translational Allergy* (*CTA*) 2018, **8(Suppl 1):**P31

**Background:** Most fish-allergic patients are sensitized to muscle parvalbumin. Clinical cross-reactions are common, but a number of patients tolerate specific fishes. The knowledge on molecular and immunological properties of parvalbumins from different fishes is essential to understand this variable clinical reactivity. Angler fish (*Lophius piscatorius*) is a food fish which is popular as a delicacy but not yet characterized concerning its potency to induce allergic reactions. The aim of this project was to analyse angler fish parvalbumins regarding their properties as putative food allergens.

**Methods:** Angler fish protein extracts were separated by gel electrophoresis, parvalbumins identified in immunoblots with specific antibodies and quantified in SDS-PAGE by densitometric analysis. cDNAs coding for parvalbumin isoforms were cloned and one isoform expressed in Escherichia coli. Natural, purified parvalbumins were analyzed regarding their IgE reactivity by ELISA, their stability towards in vitro gastrointestinal digestion and their structural properties by circular dichroism spectroscopy. The humoral immune response to angler fish parvalbumin was investigated in a BALB/c mouse model.

**Results:** Angler fish contains 0.6–1.5 mg parvalbumins per gram muscle. We identified three parvalbumin isoforms which differed by their migration behavior in SDS-PAGE (6–14 kDa), their isoelectric points (pH 4–5) and in their N-termini. Protein sequence comparison of cloned parvalbumins gave an identity of 69%, confirming the presence of true isoforms. Purified natural angler fish parvalbumins and a recombinant parvalbumin were recognized by IgE antibodies from 70% of cod-allergic individuals. The natural parvalbumins showed thermally stable alpha-helical structures sensitive to calcium depletion. Analysis of the proteins’ stability towards gastrointestinal digestion revealed that an angler fish parvalbumin isoform resisted partially to this treatment and was still detectable by specific antibodies. A mouse model substantiated that angler fish parvalbumins represent immunogenic molecules, although the humoral immune response to carp parvalbumin was stronger than to the angler fish homologs.

**Conclusions:** Angler fish parvalbumins might be important food allergens as they are stable, highly abundant and recognized by fish-allergic patients’ IgE-antibodies. Recombinant angler fish parvalbumin could be an important reagent for a future diagnostic panel of standardized molecules.

#### P32 Evolution and current status of the official allergen nomenclature system and the WHO/IUIS allergen nomenclature sub-committee

##### Richard E Goodman^1^, Anna Pomés^2^, Gabriele Gadermaier^3^, Janet M. Davies^4^, Thomas A. E. Platts-Mills^5^, Christian Radauer^6^, Andreas Loptata^7^, Andreas Nandy^8^, Jonas Lidholm^9^

###### ^1^Food Allergy Research and Resource Program, Department of Food Science and Technology, University of Nebraska-Lincoln, Lincoln, NE, USA; ^2^INDOOR Biotechnologies, Inc., Charlottesville, VA, USA; ^3^University of Salzburg, Salzburg, Austria; ^4^Institute of Health and Biomedical Innovation, Centre for Children’s Health Research, Queensland University of Technology, South Brisbane, Queensland, Australia; ^5^University of Virginia Medical Center, Department of Medicine, Charlottesville, VA, USA; ^6^Department of Pathophysiology and Allergy Research, Medical University of Vienna, Vienna, Austria; ^7^Centre for Biodiscovery and Molecular Development of Therapeutics, Townsville, Australia; ^8^Allergopharma GmbH & Co. KG, Reinbek, Germany; ^9^Thermo Fisher Scientific, Uppsala, Sweden

**Correspondence:** Richard E Goodman - rgoodman2@unl.edu

*Clinical & Translational Allergy* (*CTA*) 2018, **8(Suppl 1):**P32

**Background:** The WHO/IUIS Allergen Nomenclature system was first defined in the mid-1980’s as described in the Bulletin of the World Health Organization article 64(5):767–770 (1986). A dedicated Allergen Nomenclature Sub-Committee was formed under the World Health Organization (WHO) and International Union of Immunological Societies (IUIS). The objective is to maintain an unambiguous and consistent nomenclature system for allergenic proteins

**Methods:** The allergen nomenclature is based on an abbreviation of the genus (three or four-letters) and species (one or two-letters) with a number assigned based on naming order and protein biochemical type. Allergenic proteins previously characterized and named by authors were renamed (e.g. Group I pollen allergens of *Lolium perenne*, became Lol p I; hazelnut pollen allergen Hla of *Corylus avellana* became Cor a l. In the 1990’s many allergens were produced as recombinant proteins from cDNA, others by purification of proteins. Roman numerals were replaced with Arabic numerals (e.g. Lol p 1) and four decimal places were added for closely related isoallergens and variants. The Sub-Committee now includes a panel of 18 experts that review allergen submissions and update the database. Structurally related allergens from closely related species receive the same number designation. Individual purified proteins have to be characterized by amino acid sequence, apparent molecular weight as well as other biochemical properties, and meet the criterion of demonstrated IgE binding. The database is available at www.allergen.org. Publications from the Sub-Committee are available on the website. Scientists describing novel allergens are expected to submit the detailed application to the Sub-Committee for an official designation of name and number before publishing allergen discovery. The European Academy of Allergy and Clinical Immunology and the American Academy of Allergy, Asthma and Immunology are joining the IUIS in supporting the Allergen Nomenclature Sub-Committee and associated database.

**Results:** In August 2017, the WHO/IUIS Allergen Nomenclature Database contains 876 allergens from 265 sources including 1.259 isoallergens and variants. Requirements on the updated Submission form will be presented.

**Conclusions:** Support from IUIS, EAACI and AAAAI will help maintain the database. Authors publishing work identifying new allergens should submit their data in a confidential manner to the WHO/IUIS Allergen Nomenclature Sub-Committee, prior to submission to a journal.

#### P33 The app for allergens

##### Nishant Jha^1^, Sayeh Agah^2^, Martin Chapman^2^

###### ^1^University of Virginia, Charlottesville, VA, USA; ^2^Indoor Biotechnologies, Charlottesville, VA, USA

**Correspondence:** Nishant Jha - nj7kv@virginia.edu

*Clinical & Translational Allergy* (*CTA*) 2018, **8(Suppl 1):**P33

**Background:** Rationale: Many existing web technologies have made the jump to mobile devices. Scientific resources, however, have been slow to follow. Current allergen databases are a powerful source of bioinformatics knowledge, but their utility is diminished by a lack of accessibility. Most productive science occurs at the lab bench, away from desktop computers but accessible to mobile devices. Our aim was to develop an Android application that could provide up to date information about allergens and be immediately accessible.

**Methods:** A C ++ program was written to download HTML content from Allergen.org. These HTML files were processed through the command-line tools grep and sed, as well as through a Python program. The entries were then validated and parsed into a SQLite database. Finally, a user interface was written in XML format with underlying logic written in Java. The source code is made freely available on github.com (https://github.com/ninjha01/Mast).

**Results:** An Android application that will automatically update as new information is added to the WHO/ISIS allergen nomenclature database was successfully developed. This was made possible by constructing a web scraper that would periodically create a local, searchable database using the technologies outlined above. The app replicates functionality present in the WHO/IUIS website; allergens can be searched by name, taxonomy, source, or biochemical name. All information contained in the online database is stored in the application locally, so users are not required to maintain an internet connection—functionality that will never be present in the webpage-based implementation.

**Conclusions:** With the rise of mobile computing, scientists should expect their tools to accompany them wherever they go, whether it be the desk or the bench. The App for Allergens updates and improves a valuable bioinformatics resource, the WHO/IUIS allergen database, for allergy/immunology research. In addition, it provides an upgradeable, extendible platform that can quickly absorb changes in the database, as well as provide new features (e.g. 3D structures and offline access) and research capabilities.

#### P34 Drugs of porcine origin: A risk for patients with alpha-gal syndrome?

##### Kyra Swiontek^1^, Martine Morisset^2^, Françoise Morel-Codreanu^2^, Jana Mehlich^3^, Jörg Fischer^4^, Ulf Darsow^3^, Markus Ollert^1^, Tilo Biedermann^3^, Bernadette Eberlein^3^, Christiane Hilger^1^

###### ^1^Luxembourg Institute of Health, Esch-Sur-Alzette, Luxembourg; ^2^Centre Hospitalier Luxembourg, Luxembourg, Luxembourg; ^3^Technical University Munich, Munich, Germany; ^4^Eberhard Karls University Tübingen, Tübingen, Germany

**Correspondence:** Kyra Swiontek - kyra.swiontek@lih.lu

*Clinical & Translational Allergy* (*CTA*) 2018, **8(Suppl 1):**P34

**Background:** The alpha-gal syndrome is characterized by a delayed (3–6 h) allergic reaction to red meat and the presence of specific IgE antibodies directed at the carbohydrate epitope galactose-alpha-1,3-galactose (alpha-gal). In particular pork kidney and innards seem to be triggers that induce faster and more severe allergic reactions. Moreover, several drugs of mammalian origin have been reported to trigger allergic reactions in these patients, e.g. therapeutic antibodies (cetuximab), antivenoms and Gelofusine, a volume replacement. Objective: To determine if further drugs composed of proteins of porcine origin contain clinically relevant quantities of alpha-gal and induce positive skin tests and basophil activation in patients with alpha-gal syndrome.

**Methods:** Creon^®^ and Enzynorm^®^ f were obtained from the hospital pharmacy. Creon is a pancreatic enzyme preparation, a combination of porcine-derived lipases, proteases, and amylases indicated for the treatment of exocrine pancreatic insufficiency. Enzynorm contains mainly the protease pepsin which is obtained from porcine gastric mucosa. Both drug preparations were analysed by immunoblot and ELISA using a monoclonal antibody directed against alpha-gal and sera of patients with alpha-gal syndrome. Different fresh meat sources as well as Creon and Enzynorm were used to perform skin tests in 10 patients. Furthermore basophil activation (Flow CAST^®^) with different concentrations of a pork kidney extract and these two drugs were assessed in 14 patients with alpha-gal syndrome and 4 controls.

**Results:** The main ingredient of Enzynorm, pepsin, was shown to carry the alpha-gal epitope. Creon was shown to contain alpha-gal epitopes, but no protein carrying this epitope could be identified so far. Skin tests using Enzynorm produced systematically a stronger reaction than pork kidney. All patients were positive with Enzynorm and Creon, 9/10 with pork kidney, 5/10 with raw pork meat and 7/10 with raw beef meat. Creon showed a strong basophil activation in 13 out of 14 patients, Enzynorm in all 14 patients analysed. When compared to pork kidney extract, basophil sensitivity was found to be increased about tenfold with Creon and Enzynorm. No activation was detected in basophils of 4 control patients.

**Conclusions:** Creon and Enzynorm, both drugs of porcine origin, were shown to contain alpha-gal. The medications are assumed to be of high risk to patients with alpha-gal syndrome as shown by clear positive skin tests and strong basophil activation.

## Friday 10 November 2017

### Oral abstracts: Omics in allergy

#### O01 Identification and immunological characterization of novel *Polistes* venom allergens

##### Maximilian Schiener^1^, Christiane Hilger^2^, Bernadette Eberlein^3^, Mariona Pascal^4^, Annette Kuehn^2^, Dominique Revets^2^, Sébastien Planchon^5^, Gunilla Pietsch^3^, Pilar Serrano^6^, Carmen Moreno-Aguilar^6^, Federico De La Roca^7^, Tilo Biedermann^3^, Ulf Darsow^3^, Carsten Schmidt-Weber^1^, Markus Ollert^8^, Simon Blank^1^

###### ^1^Center of Allergy and Environment (ZAUM), Technical University of Munich and Helmholtz Center Munich, Munich, Germany; ^2^Department of Infection and Immunity, Luxembourg Institute of Health (LIH), Esch-Sur-Alzette, Luxembourg; ^3^Department of Dermatology and Allergy Biederstein, Technical University of Munich, Munich, Germany; ^4^Immunology Department, CDB Hospital Clinic de Barcelona, Universitat de Barcelona, Barcelona, Spain; ^5^Department of Environmental Research and Innovation, Luxembourg Institute of Science and Technology, Belvaux, Luxembourg; ^6^Hospital Universitario Reina Sofía and Maimonides Institute for Research in Biomedicine (IMIBIC), Córdoba, Spain; ^7^Allergy Unit, Pneumology Department, ICR, Hospital Clinic de Barcelona, Barcelona, Spain; ^8^Department of Infection and Immunity, Luxembourg Institute of Health (LIH); ^9^Department of Dermatology and Allergy Center, Odense Research Center for Anaphylaxis, University of Southern Denmark, Esch-Sur-Alzette, Luxembourg

**Correspondence:** Maximilian Schiener - Maximilian.Schiener@helmholtz-muenchen.de

*Clinical & Translational Allergy* (*CTA*) 2018, **8(Suppl 1):**O01

**Background:** Allergies due to venoms of hymenoptera can cause severe anaphylaxis in untreated patients. In the last years, progress of component-resolution advanced the differential diagnosis of honeybee and wasp venom allergic patients. To date, the discrimination between *Vespula* and *Polistes* venom allergy is still challenging, as only few allergens have been identified for component-resolved diagnostics. Both species live side to side in Mediterranean regions and the US, but with *Polistes dominula* being an invasive species, *Polistes* venom allergy is likely to evolve in more moderate climate zones of Europe. In this study, *Polistes* venom was analyzed for the presence of additional allergens. Newly identified allergens were subsequently characterized in detail to broaden the available panel of important allergens.

**Methods:** *Polistes* venom was analyzed by mass spectrometry. Identified components were cloned from venom gland mRNA and recombinantly produced in insect cells. The resulting purified proteins, together with their homologues of different hymenoptera species, were characterized by immunoblotting and assessed for IgE cross-reactivity. Moreover, their capacity to activate basophils of either honeybee or wasp venom allergic patients was evaluated.

**Results:** Several *Polistes* venom components were identified and two proteins (100 kDa and 41 kDa) were successfully produced in Sf9 insect cells together with the homologous allergens from *Apis mellifera* and *Vespula vulgaris*. The analysis of specific IgE in sera from honeybee, *Vespula* and *Polistes* venom allergic patients identified the novel components as major allergens. Additionally, basophil activation tests proved their clinical relevance. Cross-reactivity on IgE level and basophil activation indicates the presence of shared IgE epitopes, probably in conserved regions of venom proteins.

**Conclusions:** The analysis of crude *Polistes* venom identified several, yet unknown components. The two novel recombinantly produced proteins proved to be allergens of *Polistes* venom and, therefore, might become key elements for molecular diagnostics in the future.

#### O02 Early and persistent changes in MiRNA expression influencing T Cell plasticity and Th2 cytokine production are specific for epicutaneous immunotherapy in a mouse model of peanut sensitized mice and are not induced by oral immunotherapy

##### Jorg Tost^1^, Yimin Shen^1^, Camille Plaquet^2^, Elodie Roche^1^, Veronique Dhelft^2^, Vincent Dioszeghy^2^, Christian Daviaud^1^, Florence Busato^1^, Christophe Dupont^3^, Hugh Sampson^4^, Lucie Mondoulet^4^

###### ^1^CEA-IBFJ, Centre National de Recherche en Génomique Humaine, Evry, France; ^2^DBV Technologies, Paris, France; ^3^Necker Hospital, Paris, France; ^4^DBV technologies, New York, NY, USA

**Correspondence:** Jorg Tost - tost@cng.fr

*Clinical & Translational Allergy* (*CTA*) 2018, **8(Suppl 1):**O02

**Background:** Epicutaneous immunotherapy (EPIT) is a promising treatment for food allergy under clinical investigation. In animal models, EPIT seems to confer sustained unresponsiveness and prevents further sensitization. In this study, we investigated the kinetics of miRNA expression patterns underlying the therapeutic effect of EPIT and its persistence compared to placebo or oral immunotherapy protocols (OIT).

**Methods:** BALB/c mice were orally sensitized to peanut and then treated with EPIT or not treated (sham). Mice (n = 112) were sacrificed during treatment at 1, 2, 4, 6 and 8 weeks; and 8 weeks after the end of treatment. MiRNAs were analysed in sorted CD4+ cells from spleen using high-throughput sequencing on a HiSeq4000. Results: were validated in an independent experiment (n = 112) including also a group treated with OIT with mice sacrificed during treatment at 2, 4 and 8 weeks, and 8 weeks after the end of treatment by LNA-enhanced qPCR assays targeting 40 miRNAs identified in the sequencing experiment.

**Results:** Global miRNA profiles consisting of ~ 1000 miRNAs reproducibly distinguished EPIT-treated mice from controls as early as one week following initiation of treatment. Between 23 and 190 MiRNAs were found to be differentially expressed (padj < 0.05) with a large overlap of miRNAs between adjacent time points. Differentially expressed miRNAs include miRNAs controlling T cell stability and plasticity (e.g. Tregs, miR-10a) and Th2 cytokine production (e.g. miR-92a-3p and miR-423-5p). 34 miRNAs were differentially expressed eight weeks after the end of the treatment. Experiments in the second cohort confirmed large changes in miRNA early during treatment with 29 miRNAs differentially expressed at 2 weeks, and 12, 4 and 9 miRNAs at 4, 8, and 8 weeks after the end of the treatment. In contrast only a single of the selected miRNAs differed between sham and OIT treated animals.

**Conclusions:** EPIT leads to early and reproducible changes in miRNA expression shortly after the initiation of treatment differentiating EPIT from sham or OIT-treated mice and expression changes are maintained after the termination of treatment. Differentially expressed miRNAs include miRNAs in T cell plasticity and postulated targets include genes previously associated with allergy and asthma. Our study provides further evidence for the molecular alterations underlying sustained unresponsiveness in EPIT.

### Poster Discussion Session II - Topic 1: Biomarkers in allergy diagnosis

#### P35 CC chemokine receptor 8 is engaged in eosinophil migration in experimental allergic enteritis

##### Frank Blanco Pérez^1^, Maren Krause^1^, Jonathan Laiño^1^, Jörg Kirberg^1^, Stefan Vieths^1^, Stephan Scheurer^1^, Masako Toda^1^

###### ^1^Paul-Ehrlich-Institut, Langen, Germany

**Correspondence:** Frank Blanco Pérez - febp84@gmail.com

*Clinical & Translational Allergy* (*CTA*) 2018, **8(Suppl 1):**P35

**Background:** The pathological mechanism of allergic enteritis (AE) is not well known in comparison to other clinical phenotypes in food allergy. The aim of our study is to elucidate cellular and molecular mechanism of AE using a murine model. Our previous microarray analysis indicated that gene expressions of CC chemokine receptor 8 (CCR8) and its ligand, CC chemokine ligand 1 (CCL1 or I-309) were up-regulated in the inflamed tissues of AE mice (unpublished data). In the present study, we investigated the role of CCR8 in induction of AE using CCR8 knock out (KO) mice.

**Methods:** BALB/c wild type (WT) and CCR8 KO mice were sensitized by i.p. injection with ovalbumin (OVA, a major egg white allergen) plus ALUM, and challenged by feeding egg white diet. Morphological changes and granulocytes accumulation in the inflamed jejunum were assessed by histological analysis. The frequency of granulocytes in lamina propria of small intestines was assessed by FACS. Serum levels of OVA-specific IgE antibodies and concentrations of cytokines and CC chemokines in homogenates of small intestines were measured by ELISA. T cell responses in the mice were assessed by in vitro antigen-recall assay using CD4+ T-cells isolated from mesenteric lymph nodes.

**Results:** CCR8 KO mice exhibited similar inflammatory features (e.g. disrupted villi, crypto elongation and goblet hyperplasia) but less accumulation of eosinophils in the inflamed tissues, when compared to WT mice. FACS analysis showed a decreased frequency of eosinophils (CD11b- SiglecF+ cells) and an increased frequency of neutrophils (Ly6G+ CD11b+ SiglecF-cells) in lamina propria leukocytes (CD45+ cells) of CCR8 KO mice. Interestingly, the concentrations of CCL11 (eotaxin-1), but not of IL-5, another eosinophil chemoattractant, were reduced in intestinal homogenates of CCR8 KO mice, compared to those of WT mice. Production of Th2 cytokines (IL-4 and IL-5) by CD4+ T-cells and the serum levels of OVA-specific IgE antibodies were similar in both mice, suggesting that deficiency of CCR8 does not influence T cell and antibody responses upon allergen challenge.

**Conclusions:** Our results suggest that CCR8 is engaged in CCL11 production and thereby contribute to eosinophil migration to inflammatory sites in AE, whereas neutrophils migrate in a CCR8 independent mechanism. Through a better understanding of the AE mechanism, this study will provide the basis to establish a novel anti-inflammatory strategy for treatment of food allergy.

#### P36 Eosinophilic esophagitis detection based on peptide binding to eosinophil cationic protein

##### Tafarel Andrade De Souza^1^, Ana Paula Carneiro^1^, Andréia Narciso^1^, Cristina Palmer Barros^2^, Luciane Marson^2^, Tatiane Tunala^3^, Tânia Alcântara^3^, Peter Briza^4^, Fátima Ferreira Briza^4^, Luiz Ricardo Goulart^1^

###### ^1^Laboratory of Nanobiotechnology, Institute of Genetics and Biochemistry, Federal University of Uberlandia, Uberlândia, Minas Gerais, Brazil; ^2^Pediatric Department, Federal University of Uberlandia, Uberlândia, Minas Gerais, Brazil; ^3^Pathology Laboratory, Clinical Hospital, Federal University of Uberlandia, Uberlândia, Minas Gerais, Brazil; ^4^Department of Molecular Biology, University of Salzburg, Salzburg, Austria

**Correspondence:** Tafarel Andrade De Souza - tafarel.andradedesouza@sbg.ac.at

*Clinical & Translational Allergy* (*CTA*) 2018, **8(Suppl 1):**P36

**Background:** Eosinophilic esophagitis (EoE) is an inflammatory condition of the esophagus characterized by the presence of a large number of eosinophils. Currently, EoE diagnosis is based on invasive endoscopic procedures for histopathological examination in combination with the clinical history of the patient. Hence, the identification of non-invasive biomarkers would be valuable for diagnosis and monitoring of EoE. In this study, our aim was to select and validate short peptides with potential to be used as novel biomarkers for EoE detection.

**Methods:** For the biomarkers selection, we performed a comparative proteomics analysis using liquid chromatography–tandem mass spectrometry (LC–MS/MS) of esophageal biopsies from pediatric patients with eosinophilic esophagitis, gastroesophageal reflux disease and healthy individuals. Phage display technology was used to select peptides against up-regulated proteins identified in patients with EoE. A total of twelve phage clones were selected after three biopanning rounds, while their reactivity was evaluated in a phage-ELISA assay using patient mucus samples. Furthermore, sequences of the peptides were determined by phage-DNA sequencing and the binding between peptide and protein target analyzed by in silico prediction tools.

**Results:** Mass spectrometry results showed that eosinophil cationic protein (ECP) was up-regulated in EoE patients. ECP is an eosinophil granule protein that is deposited in tissues, indicating tissue damage. A high reactive ECP-binding peptide (E8) was able to distinguish mucus of eosinophilic esophagitis patients from gastroesophageal reflux disease and healthy individuals by ELISA, achieving sensitivity of 84.62, specificity of 82.72, a positive likelihood ratio of 4896, and an area under the curve of 0.84.

**Conclusions:** This is the first study to demonstrate the detection of eosinophil cationic protein using peptides isolated from a phage display library. The peptide presented herein could be a useful diagnostic tool for the detection of EoE patients.

**Acknowledgments:** This study was supported by the Brazilian funding agency Coordenação de Aperfeiçoamento de Pessoal de Nível Superior – CAPES (88881.133291/2016-01) and the Priority Program “Allergy-Cancer-BioNano Research Centre” of University of Salzburg.

#### P37 Early and late phase responses of human mast cells increase with increasing IgE affinity

##### Charlotte Hjort^1^, Peter Adler Würtzen^2^, Hans Jürgen Hoffmann^1^

###### ^1^Department of Respiratory Diseases and Allergy, Aarhus University Hospital, Aarhus, Denmark; ^2^ALK-Abelló A/S, Global Research, Horsholm, Denmark

**Correspondence:** Charlotte Hjort - charlotte.hjort@post.au.dk

*Clinical & Translational Allergy* (*CTA*) 2018, **8(Suppl 1):**P37

**Background:** We have previously shown that reactivity and sensitivity of the early response of cultured human mast cells (MCs) increase with increasing IgE affinity. Similar results have been obtained with bone marrow-derived murine mast cells (BMMCs) activated with antigens with different relative affinities for binding to FcεRI-bound specific IgE. The late phase response was surprisingly different in BMMCs; the low affinity interaction gave rise to enhanced chemokine expression, whereas the high affinity interaction resulted in an enhanced cytokine expression. Here we explore whether differences in the affinity of IgE for allergen result in a similar pattern of mediator release from human mast cells.

**Methods:** Human MCs generated from CD133+ stem cells were sensitized with pairs of recombinant human IgE clones with either high or low affinity for *Dermatophagoides pteronyssinus* antigen 2 (Der p 2). Activation of MCs was measured as upregulation of CD63 by flow cytometry. MC reactivity (fraction of MCs activated,  %CD63+ MC) and sensitivity (allergen concentration triggering a half-maximal response, EC50) were estimated by non-parametric curve fitting. The release of cytokines and chemokines from activated MCs was measured using a multiplex immunoassay based on the Proximity Extension Assay (PEA) technology (Olink, Uppsala, Sweden).

**Results:** The combination of two high affinity IgE clones significantly increased MC reactivity (*p* = 0.0286) and MC sensitivity (*p* = 0.0286) relative to a pair of low affinity IgE clones (n = 4). Interleukin (IL)-6 (*p* = 0.0187), IL-13 (*p* = 0.0018) and IL-8 (*p* = 0.003) secretion was significantly increased at high IgE affinity compared with baseline and with low affinity stimulation. Secretion of the chemokines CCL3 (*p* < 0.0001) and CCL4 (*p* < 0.0001), but not CCL2 (*p*; ns), was significantly increased at both high and low affinity stimulation compared with baseline. However, the response was not affected by IgE affinity.

**Conclusions:** The differential chemokine response at low IgE affinity could not be reproduced. Increased IgE affinity for the allergen increased MC reactivity and sensitivity, and enhanced MC cytokine, but not chemokine, response. This suggests that affinity maturation of the IgE population is likely to substantially enhance the MC response in vivo and thus the extent and characteristics of the clinical response upon allergen encounter.

#### P38 Immunomodulatory activity of An IL10-Like peptide in allergy

##### Emilia Rezende Vaz^1^, Galber Rodrigues Araujo^2^, Patricia Tiemi Fujimura^1^, Barbara Bohle^3^, Birgit Nagl^3^, Carlos Ueira-Vieira^1^, Luiz Ricardo Goulart^1^, Fatima Ferreira^2^

###### ^1^Federal University of Uberlândia, Uberlândia, Brazil; ^2^University of Salzburg, Salzburg, Austria; ^3^Medical University of Vienna, Vienna, Austria

**Correspondence:** Emilia Rezende Vaz - emiliarezendev@gmail.com

*Clinical & Translational Allergy* (*CTA*) 2018, **8(Suppl 1):**P38

**Background:** Interleukin-10 (IL-10) is an anti-inflammatory cytokine secreted by many different cells, including antigen-presenting cells, mast cells, eosinophils, B cells, and T cells. The regulatory activity of IL-10 includes the inhibition of proinflammatory cytokines involved in Th1 and Th2 differentiation, chemokines, as well as antigen-presenting and costimulatory molecules in monocytes/macrophages, neutrophils, and T cells. Within the field of allergy, to investigate the immunosuppression of allergic reactions mediated by IL-10 produced by functional Tregs during the generation of immune tolerance to allergens is of high interest. In the present study, an IL-10-like peptide was investigated for its capability of suppressing a proinflammatory immune response.

**Methods:** IL-10-like peptides were selected from a phage-displayed peptide library through their capabilities of binding to the IL-10 receptor on macrophages. Aiming to evaluate peptides action, structural analysis was carried out using in silico approaches, while immunoregulatory activity analyzes were carried out through ELISA, macrophage stimuli, mediator release and antigen-specific T cell proliferation.

**Results:** A total of 46 different peptides were selected and based in the immunoreactivity obtained in a phage-ELISA assay performed, the mimIL-10-like peptide was chosen to be further investigated. However, the other IL-10-like peptides will be also investigated in future studies. In silico analysis showed that, the mim-IL-10-like peptide selected interacts with the IL-10 receptor in the same site as the IL-10 molecule. The synthetic peptide was able to decrease lL-6, MCP-1 and TNF-α secretion in macrophage, decrease basophil degranulation and antigen-specific T cell proliferation isolated from birch pollen allergic patients.

**Conclusions:** The mimIL-10-like peptide described in this study was able to modulate the expression of proinflammatory cytokines and other events that are crucial for the development of an allergic or inflammatory response. Hence, these results suggest that the mimIL-10-like peptide has potential to be explored as an immunomodulatory compound.

**Acknowledgments:** Coordenação de Aperfeiçoamento de Pessoal de Nível Superior-CAPES (88881.135351/2016-01), the National Institute of Science and Technology in Theranostics and Nanobiotechnology (CNPq 465669/2014-0, CAPES 23038.000776/2017-54 and FAPEMIG CBB-APQ-03613-17), and the Priority Program “Allergy-Cancer-BioNano Research Centre” of University of Salzburg.

#### P39 Hazelnut specific IL-31+ Th cells predominant in primary Cor A 1 and not Cor A 9 and Cor A 14 sensitized subjects

##### Lars H. Blom^1^, Nanna Juel-Berg^1^, Kirsten Skamstrup Hansen^1^, Lars K. Poulsen^1^

###### ^1^Department of Dermatology and Allergy, Allergy Clinic, Copenhagen University Hospital, Gentofte, Denmark

**Correspondence:** Lars H Blom - lars.blom@regionh.dk

*Clinical & Translational Allergy* (*CTA*) 2018, **8(Suppl 1):**P39

**Background:** Most individuals with hazelnut allergy have milder allergic reactions to birch pollen when sensitized to the PR-10 protein Bet v 1 a homologue to the major hazelnut allergen Cor a 1. Severe symptoms to hazelnut have been shown in individuals primary sensitized to the hazelnut storage proteins Cor a 9 and Cor a 14. Th2 cells producing the cytokines IL-4, IL-5 and IL-13 constitute the majority of the allergen-specific Th cell responses in allergic diseases. However, other cytokines as IL-9 and IL-31 have recently been associated with a Th2 phenotype. To better understand the different clinical reactivity to PR-10 and storage proteins, we aimed to investigate if hazelnut-specific Th cells of primary PR-10 and storage protein sensitized individuals differ in their production of Th2 associated cytokines in a population of tree nut allergic subjects.

**Methods:** Subjects (n = 36), with a clinical reactivity to tree nuts were included. PBMCs was stained with CFSE and stimulated (2 × 10^6^ cells/mL) with and without whole hazelnut extracts (100 µg/mL) for 7 days. Allergen-specific Th cell phenotypes was analyzed by flow cytometry.

**Results:** The included individuals were first grouped by their sIgE levels in storage protein (Cor a 9+ Cor a 14 > Cor a 1), PR-10 protein (Cor a 1 > Cor a 9+ Cor a 14) and non-sensitized (< 0.35 kUA/L). As expected, the hazelnut-specific Th cells of the storage and PR-10 protein sensitized groups showed higher levels of highly differentiated IL-4+ IL-5+ Th2 cells than in the non-sensitized group. In contrast, only the hazelnut-specific Th cells of the PR-10 sensitized subjects had more IL-31+ Th2 cells compared with the non-sensitized. We next subdivided the subjects in three groups of sIgE (Cor a 1, Cor a 9 and Cor a 14) negative or positive with no birch pollen allergy as well as sIgE positive with birch pollen allergy. Interestingly, a higher frequency of IL-31+ IL-5- hazelnut-specific Th cells were found in the sIgE sensitized subjects with birch pollen allergy compared with both groups with no birch pollen allergy.

**Conclusions:** A higher frequency of the Th2 cell associated itch cytokine IL-31 was found in the hazelnut-specific Th cells of PR-10 sensitized subjects compared to the non-sensitized. We furthermore found a larger fraction of IL-31+ IL-5- hazelnut-specific Th cells in the subjects having pollen allergy indicating a different allergen-specific Th2 response in PR-10 and storage sensitized subjects.

#### P40 Effect of CTLA4-Ig on steroid responsiveness of eosinophilic asthma

##### Akio Mori^1^, Satoshi Kouyama^1^, Miyako Yamaguchi^1^, Chiemi Kumitani^1^, Akemi Ohtomo-Abe^1^, Yuto Nakamura^1^, Yasuhiro Tomita^1^, Yuto Hamada^1^, Yosuke Kamide^1^, Hiroaki Hayashi^1^, Kentaro Watai^1^, Chihiro Mitsui^1^, Kiyoshi Sekiya^1^, Yuma Fukutomi^1^, Masami Taniguchi^1^, Takayuki Ohtomo^1^, Osamu Kaminuma^1^

###### ^1^National Hospital Organization, Sagamihara National Hospital, Sagamihara, Japan

**Correspondence:** Akio Mori - moriakiojp@yahoo.co.jp

*Clinical & Translational Allergy* (*CTA*) 2018, **8(Suppl 1):**P40

**Background:** To investigate the role of CD28 signal on the steroid responsiveness in asthma, effects of CTLA4-Ig and glucocorticoid on T cell activation and asthma model was analyzed.

**Methods:** Ovalbumin (OVA) specific murine helper T cell (Th) clones were derived from either Balb/c mice immunized with OVA/CFA or DO11.10 transgenic mice expressing T cell receptor specific for OVA/H-2d. To analyze steroid responsiveness in vitro, Th clones were cultured with antigen presenting cells and OVA in the presence of various concentration of dexamethasone (DEX). Proliferative responses of were measured by 3H-thymidine incorporation. For in vivo analysis, unprimed BALB/c mice were transferred with Th clones, challenged with OVA, and administered with DEX subcutaneously. Bronchoalveolar lavage fluid (BALF) was obtained 48 h after challenge, and the number of infiltrating cells was differentially counted. CTLA4-Ig was administered intravenously.

**Results:** Steroid sensitive (SS) and steroid resistant (SR) clones were selected based on the effect of DEX on the proliferative responses of antigen-stimulated Th clones. Airway infiltration of eosinophils of mice transferred with SS clones were effectively inhibited by DEX administration. In contrast, those of mice transferred with SR clones were not significantly inhibited by DEX. Administration of CTLA4-Ig significantly suppressed the proliferation of DEX-treated SR clones in vitro, and the eosinophil infiltration of SR asthma model transferred with SR clones in vivo. In addition, CTLA4-Ig and DEX synergistically suppressed BALF eosinophilia of mice transferred with SS clones.

**Conclusions:** CD28 signal is involved in steroid responsiveness both in vitro and in vivo, and a good therapeutic target.

#### P41 Epigenetics of toll-like receptors and their role in allergy

##### Elizaveta Bystritskaia^1^, Ludmila Gankovskaya^2^, Leila Namazova-Baranova^3^, Bella Bragvadze^4^, Victor Gankovskii^3^, Victor Novoselov^5^, Oksana Svitich^6^

###### ^1^Mechnikov Research Institute of Vaccines and Sera, Sechenov First Moscow Medical University, Moscow, Russia; ^2^Pirogov Russian National Research Medical University, Moscow, Russia; ^3^Scientific Center of Children`s Health, Moscow, Russia; ^4^Mechnikov Research Institute of Vaccines and Sera, Moscow, Russia; ^5^Sechenov First Moscow Medical University, Moscow, Russia; ^6^Mechnikov Research Institute of Vaccines and Sera, Pirogov Russian National Research Medical University, Sechenov First Moscow Medical University, Moscow, Russia

**Correspondence:** Elizaveta Bystritskaia - lisabystritskaya@gmail.com

*Clinical & Translational Allergy* (*CTA*) 2018, **8(Suppl 1):**P41

**Background:** Atopy is a condition that predisposes a person to certain allergic responses. This pathology includes some diseases such as atopic dermatitis, bronchial asthma, hives, etc. Asthma particularly is one of the most prevalent chronic diseases, in which an innate immune component and epigenetic mechanisms take place (DNA methylation and regulation of gene expression by miRNA mainly).

The aim of this study is to compare the level of methylation and expression of TLR2 and TLR4 genes in atopic diseases (bronchial asthma).

**Methods:** Scrapings from the mucous membranes of the upper respiratory tract were taken from 43 children from the age of 2–7, who were treated for bronchial asthma in Scientific Center of Children`s Health. They also were divided in 3 groups: patients without any allergic diseases, autoimmune disorders or infections (16), children with moderate (13) and severe (14) asthma.

During the research the following methods were used: DNA extraction, sodium bisulfite conversion, methylation-specific PCR, restriction and detection.

**Results:** At the first stage of data analysis a strong correlation between the methylation degree and the severity of asthma was found out.

It has been shown that healthy patients get methylated or partially methylated regions in 50% of cases. There is also a slight increase of incompletely methylated sites in children with moderate asthma. In contrast to the previous groups, a small amount of unmethylated gene promoters appears in patients who developed severe form of bronchial asthma.

The same situation also holds for methylated promoter sites in TLR4. But this time the amount of unmethylated parts becomes bigger and occurs in all 3 experimental groups. It should be noted that the number of unmethylated sites in patients with severe asthma double that in healthy ones (from 25 to 50%).

Based on data for methylation, the expression profile of targeted gene promoters was also estimated.

**Conclusions:** The present study demonstrates a strong connection between methylation status and the incidence of bronchial asthma.

TLR2 and TLR4 are significant markers of innate immunity. They might be used in early case detection and in further epigenetic discovery of asthma.

#### P42 *Mycoplasma pneumoniae* with enhanced pathogenic properties is prevalent in children and teens with mycoplasma associated pneumonia and asthma

##### Tatsiana Vladimirovna Hlinkina^1^, Svetlana Andreevna Kostiuk^1^

###### ^1^Belarusian Medical Academy of Post-Graduate Education, Minsk, Belarus

**Correspondence:** Tatsiana Vladimirovna Hlinkina - kuklitsk@mail.ru

*Clinical & Translational Allergy* (*CTA*) 2018, **8(Suppl 1):**P42

**Background:** *Mycoplasma pneumoniae* is the etiological agent in about 60% of all cases of community-acquired pneumonia in children older than 7 years and teens, is associated with the development of asthma. The involvement of *M. pneumoniae* in the pathogenesis of respiratory diseases is because of the multiplicity of pathogenicity factors, the major of which is hydrogen peroxide, which is released in enzymatic reaction catalyzed by glycerol-3-phosphate oxidase (gene name MPN051). In the structure of the enzyme amino acid His in position 51 is significant, because His51 participates in proton transfer during the oxidase reaction and the rate of this process determines the rate of hydrogen peroxide formation. The aim of the study was to detect possible mutations in His51 codon containing fragment (location: from 50 to 260 in gene) of MPN051, capable to influence the rate of hydrogen peroxide formation in *M. pneumoniae* isolates.

**Methods:** *Mycoplasma pneumoniae* was isolated from sputum and throat swabs of 54 children and teens (7–17 years old) with pneumonia, 18 of them had also asthma, with a preliminary positive result of *M. pneumoniae* DNA detection by PCR. MPN051 gene of all *M. pneumoniae* isolates was tested for mutations by sequencing. On the 10th day of culturing for all *M. pneumoniae* isolates the formation of hydrogen peroxide was studied in a semiquantitative peroxide test.

**Results:** Mutations associated with reduced and enhanced levels of hydrogen peroxide production by *M. pneumoniae* were detected. Mutation A152T, leading to change His51Leu, was observed in 7 isolates of *M. pneumoniae* from children and teens with pneumonia without asthma and was associated with reduced production of hydrogen peroxide (about 4 mg/l) in comparison with *M. pneumoniae* isolates without mutations (about 10 mg/l). Mutation G163C, leading to change Asp55His, was associated with enhanced production of hydrogen peroxide (about 20 mg/l) and was prevalent in *M. pneumoniae* isolates from children and teens with pneumonia and asthma (61%). The newly appeared His55 near His51 might promote proton transfer during the oxidase reaction, thereby accelerating the formation of hydrogen peroxide and enhancing the pathogenic properties of *M. pneumoniae*.

**Conclusions:** Missense mutation G163C in MPN051 is associated with enhanced *M. pneumoniae* pathogenic properties and is prevalent in children and teens with mycoplasma associated pneumonia and asthma.

#### P43 Does MrgprB3 plays human MrgprX2 role in rat mast cell?

##### Muhammad Novrizal Abdi Sahid^1^, Shuang Liu^1^, Takeshi Kiyoi^1^, Kazutaka Maeyama^1^

###### ^1^Ehime University, Toon-Shi, Japan

**Correspondence:** Muhammad Novrizal Abdi Sahid - rizal.pela@gmail.com

*Clinical & Translational Allergy* (*CTA*) 2018, **8(Suppl 1):**P43

**Background:** Mast cells activation could occur through immunological and non-immunological pathways. In immunological pathway, the interaction between IgE and its receptor (FcεRI) and the downstream signal rose from this interaction has been widely studied and well characterized. On the other hand, the non-immunological pathway is less understood. The shade of light about this pathway starts by the report of Tatemoto et al. (2006) followed by McNeil and co-workers (2015), which states the existence of Mrgpr receptor family that important in the non-immunological pathway in human and mouse, accordingly. In the present report, we identify and characterized the Mrgpr receptor family that responsible for the non-immunological activation of rat mast cells. Rat as long as mice are common animals that used for the laboratory experiment in the wide area of study. Hence it is important to investigate the existence of Mrgpr receptor family in this organism.

**Methods:** Here we performed histamine release experiment in rat basophilic leukemia (RBL-2H3) cells transfected with the human MrgprX2 gene (named as 2H3X2 cells), un-transfected RBL-2H3 cells and rat peritoneal mast cells (RPMCs) under the activation with various dose of DNP-BSA against IgE, compound 48/80, and ciprofloxacin. The detection of Mrgpr receptor expression in wild type (Wt) and mast cells deficient rat (Ws/Ws) was also done by reverse-transcriptase polymerase chain reaction (RT-PCR). MrgprB3 silencing was performed with MrgprB3 siRNA.

**Results:** As expected, RPMCs exhibited the increase in histamine release as a function of dose of compound 48/80 as shown by 2H3X2 cells. Un-transfected RBL-2H3 cells did not show any changes in histamine release after compound 48/80 administrations. Interestingly, ciprofloxacin could not induce histamine release as shown by McNeil et al., 2015. MrgprB3, the rat orthologue of the human MrgprX2 was observed in rat skin tissues, whereas lower levels of MrgprB3 mRNA were expressed Ws/Ws rats compared with the Wt rats. In present work, we failed to down regulated the expression of MrgprB3.

**Conclusions:** In conclusion, based on the localisation of MrgprB3 and pharmacological responses of RPMCs after histamine release experiment we suggested that MrgprB3 plays human MrgprX2 role in rat mast cell. However, more study is needed to explain several discrepancies.

### Poster Discussion Session II - Topic 2: Molecular diagnosis

#### P44 ALLERT: Handheld allergens detector

##### Jamal Badir^1^, Benjamain Smits^1^, Auxane Ladang^1^, Jean-Luc Gala^1^

###### ^1^Centre de Technologies Moléculaires Appliquées/Université Catholique de Louvain, Brussels, Belgium

**Correspondence:** Jamal Badir - jamal.badir@uclouvain.be

*Clinical & Translational Allergy* (*CTA*) 2018, **8(Suppl 1):**P44

**Background:** The scope of ALLERT project is to provide a practical, portable, rapid, and effective diagnostic system to detect allergens in foods. The system includes a multiplex Lateral-Flow Immunochromatographic Assay and a handheld Reader providing a qualitative response (“yes/no”) regarding the presence of targeted allergens. This diagnostic system answers a growing need in food safety management and mainly targets agro-alimentary industries and end-user affected by a severe risk in food allergy. The device is meant to be used in remote situations from the laboratory, must therefore be portable, easy to handle and to operate by unexperienced users, be impact-resistant and withstand extreme conditions, works quickly (15 min maximum), have low production costs, and ensures a long shelf life. Furthermore, the device must provide clear and reproducible results at the cut-off level.

**Methods:** Design and construction of the multiplex detection test. Multiplexing is achieved by spotting technology, which consists of printing small quantities of antibodies and proteins in the shape of spots on the nitrocellulose membranes. Multiplex conjugate pads were made by integrating the various antibodies of interest conjugated with the gold nanoparticles.

**Results:** a panel of specific polyclonal antibodies directed against the allergens of interest (milk, egg, hazelnut, peanut, shrimp and mustard).

Development of a device for the preparation of the food samples. This easy-to-use device allows the extraction of allergens from different food matrices by a standard collection, filtration and purification technique.

Multiplex lateral flow method to detect simultanously six targeted allergens.

Photonic innovation that consists in manufacturing a signal acquisition device compatible with multiplex detection and adapted to a mobile testing. To increase the quality and the sensibility of the reader, we use the potential of the multispectral imaging approach and therefore increase the dynamic range of signal detection.

Signal analysis with a homemade software that provides an automatic qualitative interpretation of the test easily understandable by any untrained user.

**Conclusions:** This research has demonstrated the feasibility of multiplexing on a lateral flow chromatographic test. The future challenge will be to increase the level of multiplexing of the target allergens, to reduce the cross-reactions between the different molecules and to improve the quality of the signal obtained for each allergen.

#### P45 Allergen component specific IgE measurement with a novel immunoassay system: Diagnostic accuracy and intermethod comparison

##### Edsel Sinson^1^, Morkoah Blay^1^, Stephanie Ortega^1^, Sean-Xavier Neath^1^, Richard Hockins^1^, Eric Whitters^1^

^1^Research and Development, Hycor Biomedical, Garden Grove, CA, USA

**Correspondence:** Edsel Sinson - esinson@Hycorbiomedical.com

*Clinical & Translational Allergy* (*CTA*) 2018, **8(Suppl 1):**P45

**Background:** Component-resolved diagnostics (CRD) has become of growing importance in the field of clinical allergology, providing information that cannot be obtained from extract-based tests. It utilizes purified native(n) or recombinant(r) allergens to detect IgE sensitivity to individual allergen molecules facilitating more precise diagnosis of allergic diseases and identifying sensitizations attributable to cross-reactivity. The information may also aid the clinician in prescribing oral immunotherapy (OIT) in patients with severe symptoms, giving advice on allergen avoidance, or needing to perform food challenges. In this study, we evaluate the performance of allergen components on the new system assessing both diagnostic accuracy and intermethod comparison.

**Methods:** The new system is a fully-automated immunoassay platform to quantify specific IgE concentrations in human serum. It utilizes magnetic microparticles to which allergens are coupled by a process called “on-board kitting,” allowing for individual or multiple allergens to be used in a single test at the discretion of the clinician. The assay then adds 4 µL of serum to the coated beads in order to quantify sIgE concentrations for the individual allergen or “custom mix”. For this study, a total of 100 patients were used that were positive to 10 components found in either cow’s milk, egg, peanut, hazelnut, or short ragweed pollen. A panel of 10 negative patients were also tested against each component. All patients were tested on the new system and on a commercially available allergy platform (Thermo Fisher Scientific, Uppsala, Sweden).

**Results:** The overall agreement between the two systems was 95.5% (Cohen’s Kappa = 0.918; confidence interval [CI] 95%: 0.864–0.973) for nBos d 4, nBos d 5, nBos d 8, nGal d 2, nAra h 1, nAra h 2, nAra h 3, rAra h 8, rCor a 14, and nAmb a 1. A strong, positive linear correlation between the assays (r2 from 0.730 to 1.00, and Spearman’s rho from 0.746 to 0.985) was also observed. Passing–Bablok regression analysis resulted in a slope from 0.82 (95%[CI] 0.560–1.610) to 1.72 (95%[CI] 0.950–3.210). All allergen confidence intervals included the slope of 1.

**Conclusions:** The new system is capable of high diagnostic accuracy of sIgE to allergen components and provides a strong agreement with the commercially available allergy diagnostic platform.

#### P46 Impact of CCD-specific IgE on insect venom-related molecular IgE diagnostic

##### Joerg Fischer^1^, Gwendolyn Glatthaar^2^, Stephan Forchhammer^1^, Amir S. Yazdi^2^

###### ^1^Department of Dermatology, Faculty of Medicine, Eberhard Karls University Tuebingen/Department of Diagnostic Laboratory Medicine, Faculty of Medicine, Eberhard Karls University Tuebingen, Tuebingen, Germany; ^2^Department of Diagnostic Laboratory Medicine, Faculty of Medicine, Eberhard Karls University Tuebingen, Tuebingen, Germany

**Correspondence:** Joerg Fischer - joerg_fischer2@web.de

*Clinical & Translational Allergy* (*CTA*) 2018, **8(Suppl 1):**P46

**Background:** Polyclonal specific IgE (sIgE) directed against ubiquitous present glycan structures on plant or insect venom proteins, so-called cross-reactive carbohydrate determinants (CCD), have no independent clinical impact, but are a major cause for false positive double sensitization against bee and wasp venom in IgE diagnostic. Recombinant produced molecular insect allergens are CCD-free and are considered as a solution to overcome this phenomenon. Recently it was shown that the ImmunoCAP matrix adds CCD reactivity to the assay and the specificity of molecular diagnostic is also affected by CCD. Objective: To determine the degree of CCD-sIgE associated alteration of venom-related molecular IgE diagnostic with focus on decision-relevant changes.

**Methods:** The RIDA CCD-inhibitor (R-Biopharm AG, Darmstadt, Germany) was established in serological routine diagnostic of insect venom allergy. All sera were tested twice for sIgE against bee and wasp venom with extracted-based and recombinant allergens (Api m1, Ves v1, Ves v5) on an ImmunoCAP 250 automated platform (Thermo Fisher Scientific, Uppsala, Sweden) with and without pretreatment with CCD inhibitor. The effect of the CCD inhibitor was verified with an ImmunoCAP containing MUXF3 from Bromelin.

**Results:** In total the CCD inhibition procedure was applied to 20 CCD-negative samples as controls and 68 CCD-positive samples, from which n = 60 showed sufficient CCD inhibition and were included in further analysis. For bee-related molecular diagnostic CCD-related effects were found in 26.7% of the samples and 20.0% of the results had to be classified as false positive. CCD-related effects at least in one of the wasp-related recombinant assays were found 18.4 and 11.7% of the results had to be classified as false positive. Translating these results on a routine diagnostic laboratory setting for molecular diagnostic, a rate of < 5% of false positive results can be assumed.

**Conclusions:** ImmunoCAP assays with recombinant allergens are indeed partially biased by the CCD sIgE. The extent is substantially lower than the known phenomenon for extract-based allergens. CCD inhibition is a useful tool in special clinical situations but no prerequisite for a routine diagnostic laboratory.

#### P47 Complexes of peanut allergens present unique challenges for natural allergen purification

##### Sabina Wünschmann^1^, Catherine Thorpe^1^, Lisa D. Vailes^1^, Heaven Cerritos^1^, Sayeh Agah^1^, Martin D. Chapman^1^

###### ^1^INDOOR Biotechnologies Inc., Charlottesville, VA, USA

**Correspondence:** Sabina Wünschmann - sabina@inbio.com

*Clinical & Translational Allergy* (*CTA*) 2018, **8(Suppl 1):**P47

**Background:** Highly purified allergens are core components of in vitro molecular diagnostics. The absence of any allergenic impurities is a fundamental quality criterion for diagnostic allergen components. Manufacturing of high purity peanut allergens from peanut flour is known to be challenging. The aim of this study was to use mass spectrometry (LC–MS/MS) to aid the development of effective purification strategies, establish criteria of purity, and validate purified peanut allergens for use in molecular diagnostics.

**Methods:** Natural peanut allergens Ara h 1, Ara h 2, Ara h 3, and Ara h 6 were extracted from blanched or light roast peanut flour at neutral pH (7.4). Peanut allergens were purified by monoclonal antibody affinity chromatography, followed by gel-filtration- and/or hydrophobic interaction chromatography and analyzed by LC–MS/MS, ELISA, and FEIA or chimeric IgE ELISA. SDS-PAGE and Western Blots of peanut extracts and purified allergens were performed under non-reducing and reducing conditions using peanut allergen-specific monoclonal antibodies.

**Results:** Monoclonal antibody chromatography for purification of peanut allergens results in co-purification of other un-wanted peanut allergens. Western Blots of peanut extracts suggest the formation of high molecular weight complexes, notably between Ara h 1 and 2S-albumins Ara h 2 and Ara h 6. After extensive chromatographic clean-up, allergen purity assessed by LC–MS/MS, was > 93%. Immunoreactivity of purified peanut allergens was confirmed in ELISA and by FEIA or chimeric IgE ELISA using sera from peanut-allergic patients.

**Conclusions:** Optimized, ISO-9001 compliant bioprocessing pathways have been established to yield high purity natural peanut allergens. The sensitivity provided by mass spectrometry is critical to confirm allergen purity.

#### P48 Current challenges in fish allergy diagnosis: review of a Spanish cohort

##### Mariona Pascal^1^, Olga Dominguez^2^, Rosa Maria Jiménez-Feijoo^2^, Thorsten Graf^3^, Tanja Scheuermann^3^, Dominique Revets^3^, Clara San Bartolomé^1^, Jaime Lozano^2^, Monica Piquer^2^, Montserrat Alvaro^2^, Adrianna Machinena^2^, Maria Teresa Giner^2^, Markus Ollert^3^, Ana Maria Plaza-Martin^2^, Annette Kuehn^3^

###### ^1^Immunology Department, CDB, Hospital Clinic, Barcelona, Spain; ^2^Allergy and Clinical Immunology Department, Hospital Sant Joan de Déu, Esplugues De Llobregat, Spain; ^3^Department of Infection and Immunity, Luxembourg Institute of Health, Esch-Sur-Alzette, Luxembourg

**Correspondence:** Mariona Pascal - mpascal@clinic.ub.es

*Clinical & Translational Allergy* (*CTA*) 2018, **8(Suppl 1):**P48

**Background:** Fish is not only an important component in the Mediterranean diet, it is also a common elicitor of food-allergic reactions. The clinical work-up includes anamnesis, sera and skin reactivity analysis and, in some patients, oral provocations. Diagnostic algorithms allowing to predict the patients’ clinical reactivity are missing representing an important medical need. The aim of this study was to analyse the correlation of clinical tests (in vitro, in vivo) in a well-characterized Spanish cohort.

**Methods:** Fish-allergic patients (n = 34; mean age 13.1 years) were characterized by detailed clinical records, skin testing with commercial extracts (8 fishes) and ImmunoCAP sera IgE-testing (7 fishes, Gad c 1). IgE line blots were done with extracts from tuna, hake and sole. A total of 84 open food challenges was performed, in the order from tuna (canned, fresh), over hake to sole.

**Results:** Reported clinical symptoms varied from mild to severe, with patients mostly (62%) knowing about the fish species. Skin tests were positive in almost all patients (94%). IgE tests were all positive with extracts, although variable for the different fishes. Specific IgE to parvalbumin (Gad c 1) was positive in 82% of the patients while line blots revealed IgE-reactivity to homologs at variable levels (tuna, 15%; hake 15%; sole 68%), indicating that conformational epitopes might be more common than linear epitopes. IgE-binding was also found for other allergens, such as enolases (tuna, hake, sole; each 9%), aldolases (tuna, 12%; hake 44%; sole 24%) and other 30 to 40 kDa-proteins (tuna, 18%; hake 15%; sole 24%). Food challenges to canned tuna were all negative (n = 29) whereas oral provocations were positive to fresh tuna in 14% (4/29), to hake in 40% (6/15) and to sole in 27% (3/11). Data from sera analysis as well as skin test were found to correlate poorly with results from diagnostic food challenges.

**Conclusions:** The integration of large datasets, ranging from anamnesis, skin testing, allergen-based IgE-measurement, are an important challenge for clinicians in today’s clinical practice. A significant number of children may tolerate specific fishes but this still needs to be confirmed by oral challenges as the golden standard.

#### P49 A new multiplex IgE diagnostic test based on nanobeads: allergen IgE binding, reproducibility and comparative performances toward three different singleplex testing systems

##### Adriano Mari^1^, Chiara Rafaiani^1^, Michela Ciancamerla^1^, Claudia Alessandri^1^, Danila Zennaro^1^, Rosetta Ferrara^1^, Maria Livia Bernardi^1^, Lisa Tuppo^2^, Ivana Giangrieco^2^, Maurizio Tamburrini^2^, Maria Antonietta Ciardiello^2^

###### ^1^Centri Associati di Allergologia Molecolare - CAAM, Rome, Italy; ^2^Istituto di Bioscienze e Biorisorse, Consiglio Nazionale delle Ricerche, Naples, Italy

**Correspondence:** Adriano Mari - adriano.mari@caam-allergy.com

*Clinical & Translational Allergy* (*CTA*) 2018, **8(Suppl 1):**P49

**Background:** Allergy diagnosis can be performed in vivo using a restricted number of extracts, whereas extracts and allergenic proteins can be tested in vitro by measuring specific IgE. Multiplex testing tools are available since more than 10 years. They allow to get many IgE results from a small blood sample. Recently the FABER^®^test has been released. It represents a new generation of in vitro diagnostic devices using nanobeads for allergen immobilization.

**Methods:** To detail the overall set up of the FABER^®^ test and some of its performances. FABER^®^ test bears 244 allergenic preparations, namely 122 molecules and 122 extracts, coupled to nanobeads. The particles are arrayed to a solid phase matrix and allow a one-step comprehensive array-based testing solution needing only 120 µL of serum per test. Each allergen particle population can be individually optimized to achieve the maximum testing performance. The Biorad Lyphocheck Allergen IgE (BL-IgE) is a standard polyclonal commercial preparation obtained by pooling human sera.

**Results:** BL-IgE has been used for the evaluation of the specific IgE. By means of BL-IgE 174 out of 244 allergens gave positive IgE results. BL-IgE is supplied after being tested on 3 different IgE commercial testing systems (ImmunoCAP, Immulite, Hytech). IgE mean values and ranges are provided. Twelve allergen extract results out of 15 provided with the BL-IgE were used for comparison: Alt a, Ara h, Art v, Asp f, Bet v, Bos d, Can f, Der p, Equ c, Fel d, Gal d, Phl p. BL-IgE was tested on 22 consecutive FABER batches, and extract to extract comparison was performed when the same was available on FABER^®^. FABER^®^ IgE, expressed as arbitrary units (FIU), gave the following results: Ara h, overlapping with CAP-IMM-HYT; Art v, slightly below CAP, overlapping with IMM, above the HYT; Bet v, slightly below CAP-IMM; Bos d, above CAP-IMM-HYT; Can f, slightly below CAP-IMM, overlapping with HYT; Fel d, reproducible but below the 3 systems; Gal d, below IMM, overlapping with CAP-HYT; Phl p, overlapping with CAP-IMM. Alt a 1 performed better than CAP-HYT, overlapping with IMM. The 6 Der p FABER allergens gave overlapping results with the 3. Moreover our study disclosed IgE binding to allergens not declared in the BL-IgE data sheet (e.g. Cup a 1, Pru p 3), mostly all the molecule detected specificities (e.g. mite allergens) and all the IgE co-recognized preparations (e.g. egg allergens).

**Conclusions:** FABER^®^ IVD is a new lab test for multiplex specific IgE detection using allergenic molecules and extracts showing very good performances. All steps in assembling the test are verified and all allergens bind IgE. The 3 systems do not overlap each other as they have different reference standards. FABER^®^ IgE measurements performs very well with almost all allergens and it helps to disclose unknown sensitizations.

#### P50 Usefulness of component Ana O3 IgE in comparison with specific IgE and skin prick test in the diagnosis cashew allergy in children

##### Ariel Tsai^1^, Gill Vance^1^, Louise Michaelis^1^, James Gardner^1^

###### ^1^Great North Children’s Hospital, Newcastle Upon Tyne, United Kingdom

**Correspondence:** James Gardner - jgardner@nhs.net

*Clinical & Translational Allergy* (*CTA*) 2018, **8(Suppl 1):**P50

**Background:** Recent studies on cashew nut allergy suggest that the prevalence of cashew nut allergy is increasing likely due to changes in eating habits and increase in consumption.

Studies by Savvatianos et al. (2015) and Van der Valk (2016) suggested that sensitisation to Ana o 3 is highly predictive of clinical reactivity in cashew nut sensitised patients. Sensitisation to Ana o 3 indicates a primary cashew nut allergy and is known to be associated with systemic reactions. However there is limited literature.

**Methods:** We performed a retrospective analysis of Ana O3 IgE results children aged < 18 years over 12 months at a tertiary paediatric hospital. 21.6% (8/37) had history of acute anaphylaxis while 13.5% had only been sensitised but never eaten cashew. Out of the 37 children who had Ana O3 tested, 15 underwent hospital based open food challenge (OFC) at the mean age of 9.4 years (1.4–16.9).

**Results:** 80% of children whom participated OFC passed their challenges. All of 3 who failed OFC had other nut allergies. 7% (4/15) of children whom had the challenges did not have personal history of atopy. Further analysis showed that Ana O3 at threshold of < 0.35 kUA/L and < 1kUA/L provided satisfactory specificity (100%), positive predictive value (PPV at 100%). Cashew specific IgE at the cut-off of < 0.35 had a better sensitivity (100% as compared to 66.7%) and negative predictive value (NPV at 100% as compared to 92.3%). Sensitivity, specificity, PPV and NPV profile for cashew IgE threshold < 3.5 (Grade 2) was similar to those for Ana O3 threshold at < 1. Skin prick test were neither sensitive or specific in the diagnosis of cashew allergy (Table 1).

**Table 1:** Results:

Test	Threshold	Sensitivity (%)	Specificity (%)	Positive predictive value (%)	Negative predictive value (%)
Ana O3	< 0.35	66.7	100	100	92.3
Ana O3	< 1	33.3	100	100	85.7
Cashew IgE	< 0.35	100	66.7	42.9	100
Cashew IgE	< 0.7 (Grade 1)	66.7	66.7	33.3	88.9
Cashew IgE	< 3.5 (Grade 2)	33.3	100	100	85.7
SPT	< 3 mm	66.7	33.3	20	80
SPT	< 5 mm	33.3	50	14.2	75

**Conclusions:** Ana O3 might be used in addition to cashew IgE in the selection of children undergoing hospital based OFC while Cashew IgE < 0.35 may be a good guide for choosing patients for home OFC. Although the application of component resolved IgE testing for cashew allergy may have improved diagnostic characteristics than the use of cashew extract alone, it cannot yet replace clinical history and oral food challenge.

#### P51 Mast cell Tryptase is a marker of *Aspergillus fumigatus* Asp F 1-host crosstalk in cystic fibrosis patients

##### Carine Gomez^1^, Ania Carsin^2^, Marion Gouitaa^3^, Martine Reynaud-Gaubert^1^, Jean-Christophe Dubus^2^, Jean-Louis Mège^4^, Stéphane Ranque^4^, Joana Vitte^4^

###### ^1^Aix-Marseille Univ, APHM Assistance Publique Hôpitaux de Marseille, Hôpital Nord, Centre de Ressources et de Compétences en Mucoviscidose, Marseille, France, Metropolitan; ^2^Aix-Marseille Univ, APHM Assistance Publique Hôpitaux de Marseille, Hôpital Timone Enfants, Pneumo-pédiatrie, Centre de Ressources et de Compétences en Mucoviscidose, Marseille, France, Metropolitan; ^3^Aix-Marseille Univ, APHM Assistance Publique Hôpitaux de Marseille, Hôpital Nord, Service de Pneumologie, Marseille, France, Metropolitan; ^4^Aix-Marseille Univ, APHM Assistance Publique Hôpitaux de Marseille, IHU Méditerranée Infection, Marseille, France, Metropolitan

**Correspondence:** Joana Vitte - jvitte@hotmail.fr

S. Ranque and J. Vitte share senior authorship.

*Clinical & Translational Allergy* (*CTA*) 2018, **8(Suppl 1):**P51

**Background:** Cystic fibrosis (CF) is a life-limiting autosomal recessive genetic disease. Quality of life and survival mainly depend on pulmonary status, and thus on the management of pathogen assaults. For patients with end-stage pulmonary disease, lung transplantation (LTx) is an established therapy and its commonest indication in patients under 50 is CF. *Aspergillus fumigatus* (Af) is a major threat in CF, asthmatic and immunosuppressed patients. Af antigens induce immunoglobulin (Ig) G or IgE-and engage in direct interactions with mast cells (MC). Lung MC are involved in antifungal defenses and allergic inflammation to fungi. In normal airways, the mucosal MC population is tryptase-producing (MCT) while chymase- and tryptase-producing MC (MCTC) reside in the submucosa. MCT and MCTC contain similar amounts of the MC-specific tryptase. The relative abundance and functions of lung MCT and MCTC are altered during bronchial inflammatory processes driven by Af, CF or asthma. Aim: To evaluate serum baseline tryptase (sbT) as a potential biomarker of the Af-host interaction in CF patients.

**Methods:** 79 CF and 7 asthmatic patients were studied. Serum baseline tryptase (sbT), total and specific IgE and IgG directed to Af extract and recombinant allergens Asp f 1, 2, 3, 4 and 6 were measured with ImmunoCAP (Thermo Fisher Scientific, Sweden). IgG to Af were measured with an ELISA kit (Orgentec, France). Anti-Af precipitins were evaluated by immunoelectrophoresis (IEP, Sebia, France).

**Results:** Median sbT was 3.4 µg/L (1–21.6). In 83/86 patients, sbT was lower than 11.5 µg/L. In multivariate analysis (age, sex, total IgE, Af-IgE, Af-IgG, IEP, culture) sbT was statistically significantly associated with Af-IgE (*p* < 0.001). sbT was higher in Af-IgE-negative (neg) compared to Af-IgE-positive (pos) patients. This finding held true for head-to-head comparison: adult patients with native lungs, adult LTx recipients, and children. sbT levels varied as a function of Asp f 1 responses according to the Af-IgE status: In Af-sIgE-neg patients, sbT levels were negatively correlated with IgG to Asp f 1, while in Af-IgE-pos patients, sbT levels were negatively correlated with IgE to Asp f 1.

**Conclusions:** Asp f 1, a ribonuclease secreted during Af germination, is highly specific of Af and a potent inducer of IgG and IgE. Our results suggest that early steps in the Asp f 1-CF host interaction and progression from “default” Af-IgG to Af-IgE responses may affect MC regulation. In Af-IgE-neg CF patients, the negative correlation between sbT and IgG to Asp f 1 is in line with the inhibitory effect exerted by IgG on IgE-dependent MC activity. In Af-IgE-pos CF patients, the negative correlation between sbT and IgE to Asp f 1 may be linked to a chymase effect. CF lungs contain large numbers of MCTC. Chymase has been shown to decrease IgE and allergic inflammation through IL-33 modulation. IgE-independent chymase exocytosis following direct MCTC-microbial interaction and direct Af-MC interaction have been reported. sbT may thus be a novel systemic biomarker candidate for CF lung disease monitoring.

#### P52 The most comprehensive allergen panel to diagnose peanut sensitization using a new multiplex nanobead-based biochip

##### Adriano Mari^1^, Claudia Alessandri^1^, Danila Zennaro^1^, Rosetta Ferrara^1^, Maria Livia Bernardi^1^, Lisa Tuppo^2^, Ivana Giangrieco^2^, Chiara Rafaiani^1^, Michela Ciancamerla^1^, Maurizio Tamburrini^2^, Maria Antonietta Ciardiello^2^

###### ^1^Centri Associati di Allergologia Molecolare - CAAM, Rome, Italy; ^2^Istituto di Bioscienze e Biorisorse, Consiglio Nazionale delle Ricerche, Naples, Italy

**Correspondence:** Adriano Mari - adriano.mari@caam-allergy.com

*Clinical & Translational Allergy* (*CTA*) 2018, **8(Suppl 1):**P52

**Background:** Peanuts, *Arachis hypogaea*, are edible seeds enclosed in pods, belonging to the Leguminosae or Fabaceae family. Peanuts provide a lot of nutritious into just one serving. Peanut allergy is generally lifelong, it can be responsible of mild to very severe allergic reactions, ranging from rhino conjunctivitis to anaphylactic shock. So far seventeen allergenic proteins have been identified in peanuts as reported in the Allergome database. FABER^®^ test is a new nanobead-based IVD for specific IgE detection using 122 molecular allergens and 122 allergenic extracts, coupled to chemically activated nano-particles. The aim of this study is to investigate the different profiles of peanut sensitization by means of FABER^®^ test.

**Methods:** The analyzed allergenic preparations, all spotted on the FABER^®^ chip, were peanut extract (Ara h) and Ara h 1, Ara h 2, Ara h 3, Ara h 6, Ara h 9, Ara h Agglutinin, along with markers for IgE-CCD reactivity and plant allergens belonging to other groups.

**Results:** Out of 1807 routinely tested patients, 293 (16%), turned out to be sensitized to at least one peanuts allergenic preparation, with the following prevalence calculated on the 293 patients: Ara h 73%, Ara h 1 39%, Ara h 2 13%, Ara h 3 32%, Ara h 6 12%, Ara h 8 28%, Ara h 9 21%, Ara h Agglutinin 15%. Within the peanut sensitized patients 42% turned out to be positive also to Pru p 3, the most recognized LTP, 53% to Arm r HRP, the most recognized CCD marker and 22% to Bet v 1, the most recognized PR-10 allergen. The number of simultaneously recognized peanut proteins decreases with the age of the patients.

**Conclusions:** The results confirm differences in sensitization towards different allergenic peanuts protein. Filtering the sensitization results by positive tests, taking into account the chance of co-sensitization to panallergens and CCD, the decision making if peanuts have to be excluded from the diet and if they are to be considered a real risk become easier with the FABER^®^ IgE test.

#### P53 Quantitative measurement of house dust mite allergen-specific IgE at the point-of-care

##### Petra Zavadakova^1^, Aurélie Melgar^1^, Fabien Rebeaud^1^

###### ^1^Abionic SA, Epalinges, Switzerland

**Correspondence:** Petra Zavadakova - fabien.rebeaud@abionic.com

*Clinical & Translational Allergy* (*CTA*) 2018, **8(Suppl 1):**P53

**Background:** The abioSCOPE is a point-of-care IVD platform which brings diagnostic test results conveniently and immediately to the physician’s office. Quantitative IgE antibody assays are prepared with highly pure allergen components immobilized in biosensors assembled in a disposable capsule. These tests allow rapid identification of a patient’s sensitization pattern, thus assessing the risk of severe reaction and assisting Allergists to take the best clinical management decision.

We report on the analytic performance characterization of a capsule containing four major allergens of the house dust mite (HDM) species *D. pteronyssinus* and *D. farinae* (Der p 1, Der p 2, Der f 1 and Der f 2). Sensitization to HDM is very common worldwide and is an important cause of asthma and rhinitis. Lack of standardization of HDM extracts supports the use of molecular components for the identification of genuine sensitizations.

**Methods:** Human serum samples were analyzed on abioSCOPE (Abionic SA) and on ImmunoCAP ISAC or Phadia Laboratory System (ThermoFisher Scientific) according to manufacturer’s instructions. Overall agreements were computed as the number of tests results measured positive or negative on both methods (assay cut-off at 0.7 kUA/L on abioSCOPE; 0.3 ISU on ImmunoCAP ISAC) divided by the total number of samples.

**Results:** The nanofluidic technology used in these biosensors relies on the enhancement of molecular interactions in a nanometer-size channel. The excellent analytic specificity of the tests is highlighted by the absence of signal in nonallergic patient samples. Clinically elevated total and allergen-specific IgE levels did not interfere with test results. Overall agreements of 100% for group 2 allergens, and 78% (Der p 1) and 80% (Der f 1) allergens were found in a method comparison of the investigated tests with the respective tests in ImmunoCAP ISAC, a semi-quantitative multiplex IgE antibody array used in laboratories.

**Conclusions:** Thanks to novel point-of-care technologies, IgE antibody levels can today be obtained almost immediately, at no quality cost and for the benefit of the patient. The intuitive handling, minute blood volume and short turnaround time offer opportunities for allergy testing not only in Allergy practices, but also in primary cares (GPs, Pediatricians). Further test panels are currently in development with a strong focus on evaluating the impact of using allergy tests on abioSCOPE at the point-of-care in comparison to sending samples to laboratory.

#### P54 Blocking CCD specific IgE-antibodies in a multiplex environment

##### Martina Aumayr^1^, Peter Forstenlechner^1^, Heinz Kofler^2^

###### ^1^Macro Array Diagnostics, Vienna, Austria; ^2^Allergieambulatorium Hall, Hall In Tirol, Austria

**Correspondence:** Martina Aumayr - forstenlechner@macroarraydx.com

*Clinical & Translational Allergy* (*CTA*) 2018, **8(Suppl 1):**P54

**Background:** Cross-reactive carbohydrate determinants (CCDs) are a common cause for elevated in vitro test results, especially when allergen sources of plant or insect venom origin are tested. This leads to results that can take a long time to interpret and explain to patients. A new multiplex diagnostic tool (Allergy Explorer^®^, ALEX) promises to block the binding of CCD specific IgE antibodies.

**Goal:** We investigated the CCD IgE antibody blocking efficiency of the ALEX test system and its impact on the effort of interpreting IgE test results. A further goal was to evaluate the performance of the two CCD markers spotted on this system (Ana c 2 and Hom s LF, human Lactoferrin—expressed in rice cells).

**Methods:** 9 CCD positive samples (determined by ImmunoCAP^®^ o214, MUXF3) were tested with ALEX before and after CCD blocking (performed with MUXF-HSA).

**Results:** All samples showed positive results for Hom s LF and four out of nine samples were found positive for Ana c 2 prior to CCD blocking. After blocking of CCD specific IgE antibodies the results for Hom s LF were decreased by 100% in 6 out of 9 samples. The other three samples showed a blocking rate of 82–92%. The Ana c 2 positive results were reduced to one positive result, which was reduced by 65%.

In a plethora of results a 100% CCD antibody blocking rate was observed for grass pollen, certain tree pollen, weed pollen, cereals, fruit, legumes, mussels, nuts, seeds, spices, vegetables, hymenoptera venoms, latex and *Ficus benjamina*.

Prior to blocking 446 positive results were obtained with ALEX. After blocking 117 positive results remained.

Sera with a lower MUXF3 value (2.0–4.0 kUA/L) showed a CCD blocking efficiency (BE, based on Hom s LF) of 87–100%, one serum with 12.8 kUA/L showed a 100% BE and one sample with 26.7 kUA/L showed 82% BE.

**Conclusions:** Based on our results Hom s LF is superior to Ana c 2 as a CCD marker, as 9 out of 9 samples were found positive compared to 4 out of 9 for Ana c 2.

The dramatic decrease in positive sIgE results (74% fewer positive results), especially for pollen, foods, latex and hymenoptera venoms lead to a shortened time for interpretation and explaining results to patients. Patient’s anxieties over positive test results can also be reduced, especially for high risk allergen sources.

#### P55 Diagnostic value of positive result for recombinant allergen Api M 1 and Ves V 5 determined by IgE multiplex test ImmunoCAP ISAC: results of multicenter study

##### Urska Bidovec Stojkovic^1^, Martina Vachova^2^, Mira Silar^1^, Ziga Kosnik^1^, Mitja Kosnik^1^, Petr Panzner^2^, Peter Korosec^1^

###### ^1^University Clinic of Respiratory and Allergic Diseases, Golnik, Slovenia; ^2^Department of Immunology and Allergology, Faculty of Medicine and Faculty Hospital in Plzen, Charles University in Prague, Prague, Czech Republic

**Correspondence:** Urska Bidovec Stojkovic - urska.bidovec@klinika-golnik.si

*Clinical & Translational Allergy* (*CTA*) 2018, **8(Suppl 1):**P55

**Background:** In the allergy field, the IgE multiplex test ImmunoCAP ISAC (ISAC) is an additional diagnostic tool for the assessment of complex cases. When using the ISAC chip in a routine clinical laboratory setting positive result for recombinant allergen Api m 1 and Ves v 5 is frequently observed. In this multi-center study, we evaluated the meaning of this positive result and its clinical relevance.

**Methods:** This study evaluated results of 2877 ISAC test for rVes v 5 and/or rApi m 1. Positive results for rVes v 5 and rApi m 1 from ISAC were compared with ImmunoCAP (CAP) results. Clinical relevance of the result for honeybee venom or wasp venom allergy was established based on a patient clinical history.

**Results:** From all analyzed ISAC results 12% had positive IgE antibodies against rVes v 5 and 9% against rApi m 1 allergen. Positive IgE reactivity for rVes v 5 and rApi m 1 determined with ISAC was compared with CAP’s reactivity for the two allergens on 118 and 37 patients, respectively. Among them 85 (55%) patients had clinical data available; in 29% wasp and in 40% honeybee venom allergy was clinically confirmed. Diagnostic sensitivity/specificity of ISAC in comparison to CAP was for rVes v 5 94%/87% and for rApi m 1 75%/95%, respectively.

**Conclusions:** Diagnostic accuracy, for major Hymenoptera venom allergens, of ISAC multiplex IgE assay is comparable to ImmunoCAP singleplex IgE test. Thus all positive results for rVes v 5 and rApi m 1 determined with ISAC have to be interpreted carefully by the clinician with relevant clinical data of the patient.

#### P56 A new multiplex diagnostic test for identification of the appropriate venom immunotherapy

##### Styliani Taka^1^, Dimitris Mitsias^2^, Panagiota Stefanopoulou^3^, Nikolaos G. Papadopoulos^4^

###### ^1^Department of Allergy and Clinical Immunology, 2nd Paediatric Clinic, National and Kapodistrian University of Athens 2.2. StArtBio P.C, Molecular Allergy Diagnostics and Biotechnology services, Athens, Greece; ^2^Department of Allergy and Clinical Immunology, 2nd Paediatric Clinic, National and Kapodistrian University of Athens, Athens, Greece; ^3^Department of Allergy and Clinical Immunology, 2nd Paediatric Clinic, National and Kapodistrian University of Athens 2. StArtBio P.C, Athens, Greece; ^4^Department of Allergy and Clinical Immunology, 2nd Paediatric Clinic, National and Kapodistrian University of Athens, Greece 3. University of Manchester, Center for Pediatrics and Child Health, Manchester, United Kingdom

**Correspondence:** Styliani Taka - takastella@hotmail.com

*Clinical & Translational Allergy* (*CTA*) 2018, **8(Suppl 1):**P56

**Background:** Hymenoptera venom allergy (HVA) is a potentially life-threatening allergic reaction following stings to bees, wasps and polistes. The only treatment that can potentially prevent further severe reactions is venom immunotherapy (VIT) which is effective, with long-term clinical benefits. The correct identification of the allergy-relevant insect is prerequisite for accurate therapy of venom-allergic patients. We aimed to investigate a new component based multiplex sIgE test in diagnosing patients with Honey bee (HB), Common Wasp (CW) and/or Paper Wasp (PW) venom allergy and in accurately predicting the appropriate VIT.

**Methods:** Patients from Greece (n = 42) with at least one systemic reaction to insect venom were recruited and characterized in detail with a clinical questionnaire, skin prick testing (SPT) and sIgE measurement (ImmunoCAP). Here, we established a prototype assay including a panel of extracts and recombinant allergens from HB, CW and PW for molecular dissection of IgE reactivities in patients with HVA. Recombinant venom allergens (i208, i209, i210, i211, i213, i216, i220, i221), venom extracts (i1, i3, i75, i77) and a CCD‐marker were immobilized on membrane chips assembled as a multi‐parameter test for sIgE testing (EUROLine). The capability of prediction of the appropriate (i.e. finally prescribed) VIT was evaluated.

**Results:** For this purpose 42 serum samples were used to measure a panel of 12 allergens and compare with the respective values of the golden standard sIgE tests ImmunoCAP. Significant correlation of absolute values from multi‐parameter test and sIgE test was observed for all the comparable allergens (i1; R2 = 0.83, i3; R2 = 0.87, i75; R2 = 0.6, i208; R2 = 0.93, i209; R2 = 0.94, i210; R2 = 0.92, *p* = 0.00; and for i211; R2 = 0.6 and *p* = 0.023). More important, the VIT prediction from the new multiplex method was in agreement in 90.5% of those patients ultimately took based on the overall clinical diagnosis using the sIgE tests. The remaining 9.5% regards patients that were finally given double VIT (instead of one suggested by the Euroline DPA-Dx insect venom method) and this decision was based on clinical history and the inability to confidently exclude one venom.

**Conclusions:** In conclusion, the Euroline DPA-Dx insect venom profile was able to identify the culprit venom in our cohort. Given that there is a high prevalence of double, or even multiple, positive extract-based results, the need for an accurate component resolved diagnostic tool is important to avoid unnecessary VIT courses.

### Poster Discussion Session II - Topic 3: Immunotherapy/clinical studies

#### P57 Allergy confers protection against glioblastoma expansion

##### Aurélie Poli^1^, Anaïs Oudin^2^, Vincent Puard^2^, Olivia Domingues^1^, Oliver Hunewald^1^, Virginie Baus^2^, Tatiana Michel^1^, Gunnar Dittmar^2^, Simone P. Niclou^2^, Jacques Zimmer^1^, Markus Ollert^1^

###### ^1^Luxembourg Institute of Health, Department of Infection and Immunity, Esch-Sur-Alzette, Luxembourg; ^2^Luxembourg Institute of Health, Department of Oncology, Luxembourg, Luxembourg

**Correspondence:** Aurélie Poli - aurelie.poli@lih.lu

*Clinical & Translational Allergy* (*CTA*) 2018, **8(Suppl 1):**P57

**Background:** Glioblastoma (GBM) is the most common and deadly primary malignant brain tumour worldwide. It is invariably fatal with a median life span of less than 15 months despite therapeutic treatments including a combination of surgery with chemo- and radiotherapy. Therefore, there is an urgent need for the development of new prognostic markers and treatment modalities.

Many epidemiological studies emphasised an inversed correlation between pre-existing IgE-mediated allergy and GBM risk. Indeed, such an allergy was correlated with a reduction of the risk to develop a GBM later in life by 20–40%. Epidemiological investigations demonstrated that GBM patients presenting high levels of total IgE in their serum live 9 months longer on average compared to those with lower levels. The intrinsic immunobiological and molecular mechanisms responsible for these correlations are not implicit and require more in-depth exploration.

**Methods:** We postulated that allergies may promote a state of increased immuno-surveillance in the brain through the presence of immunological factors such as immunoglobulins, cytokines and cells involved in Th2-driven allergic reactions. Such factors may favour the elimination of the nascent tumour in brain parenchyma. Thus, we implemented a long term allergic airway inflammation in a GBM mouse model.

**Results:** We demonstrated an increase of the animal survival that was correlated with a delayed tumour engraftment and a reduced tumour growth. These phenotypes were associated with functional modification of microglia from sensitized mice. Indeed, these microglia showed a rise in the production of IL-6 and TNF-alpha as well as an increase in cytotoxic functions against a syngeneic GBM cell line ex vivo.

**Conclusions:** These findings implicate microglia as a potential player favouring the anti-GBM effect conferred by the allergy state. Future investigations should delineate the molecular pathways and events by which the Th2-driven allergic reactions protect against GBM development focusing on microglia in our murine model with the aim of developing more effective treatments.

#### P58 IgE Epitope capping in the major apple allergen Mal D 1 by phenolic compounds and vitamin C

##### Linda Ahammer^1^, Reiner Eidelpes^1^, Jana Unterhauser^1^, Anna Sophia Kamenik^2^, Klaus Roman Liedl^2^, Kathrin Breuker^1^, Martin Tollinger^1^

###### ^1^Institute of Organic Chemistry, Innsbruck, Austria; ^2^Institute of General, Inorganic and Theoretical Chemistry, Innsbruck, Austria

**Correspondence:** Linda Ahammer - linda.ahammer@uibk.ac.at

*Clinical & Translational Allergy* (*CTA*) 2018, **8(Suppl 1):**P58

**Background:** The major apple allergen Mal d 1 (*Malus domestica*) is the predominant cause of apple allergies in large parts of Europe and Northern America. Allergic reactions against this 17.5 kDa protein are the consequence of initial sensitization to the structurally homologous major allergen from birch pollen, Bet v 1. Consumption of apples can subsequently provoke immunologic cross-reactivity of Bet v 1-specific antibodies with Mal d 1 and trigger severe oral allergic syndromes, affecting more than 70% of all individuals that are sensitized to birch pollen.

After treatment with polyphenol oxidase (PPO) in the presence of catechin the IgE-binding by Mal d 1 decreases. Our recently published structure of Mal d 1 provides the basis for elucidating this effect on a molecular level.

**Methods:** NMR Spectroscopy, Mass Spectrometry, MD Simulation

**Results:** We obtained different covalently modified Mal d 1 protein by adding polyphenolic compounds and vitamin C. Localization of these modifications by mass spectrometry and NMR spectroscopy reveals reduced accessibility of the IgE epitope on the Mal d 1 surface. A three-dimensional structural model for modified Mal d 1 is presented.

**Conclusions:** Our data suggest that apple cultivars with a high content of polyphenols and vitamin C may be less allergenic and therefore better suited for apple allergic individuals.

#### P59 Induction of cross-reactive Bet V 1-specific IgG antibodies by BM4, a hypoallergenic candidate for AIT of birch pollen allergy

##### Lorenz Aglas^1^, Frank Stolz^2^, Paulina Chrusciel^3^, Ville Ranta-Panula^3^, Ulla-Marjut Jaakkola^3^, Laurian Jongejan^4^, Ronald Van Ree^4^, Angela Neubauer^2^, Fatima Ferreira^1^

###### ^1^University of Salzburg, Salzburg, Austria; ^2^Biomay AG, Vienna Competence Center, Vienna, Austria; ^3^Central Animal Laboratory of the University of Turku (UTUCAL), Turku, Finland; ^4^Academic Medical Center, Amsterdam, the Netherlands

**Correspondence:** Lorenz Aglas - lorenz.aglas@sbg.ac.at

*Clinical & Translational Allergy* (*CTA*) 2018, **8(Suppl 1):**P59

**Background:** In course of the EU-funded initiative “BM4SIT – Innovations for Allergy” (www.bm4sit.eu), the efficacy of the hypoallergenic Bet v 1 derivative BM4 is evaluated in order to be used as novel AIT vaccine for the treatment of birch pollen allergy.

Within the present study we evaluated the humoral immune response induced by immunizations of Wistar rats with BM4. We further investigated the cross-reactivity of the induced serological antibodies to the major birch pollen allergen Bet v 1.

**Methods:** Rats were immunized bi-weekly for a period of 26 weeks with either the intended human clinical dose of BM4 (80 μg), a high dose (160 μg BM4), or placebo. Aluminum hydroxide was used as adjuvant. Animals of the main group (3 × 20 rats) were sacrificed 1 week after the last injection. Animals of the recovery group (3 × 10 rats) were sacrificed after a 6-week observation period. BM4- and Bet v 1-specific IgE, IgG1, IgG2a, and IgG2b levels in rat sera were determined by ELISA.

**Results:** We found that BM4 was able to induce high titers of BM4-specific IgG1, IgG2a and IgG2b antibodies (105-, 103- and 102-fold higher compared to the placebo group, respectively) upon immunization with either 80 or 160 µg of BM4. No significant differences in the levels of IgG2a, IgG1, and IgG2b were observed between the two groups receiving different amounts of BM4. The BM4-induced IgG1, IgG2a and IgG2b antibodies were cross-reactive with Bet v 1. In contrast, the IgE levels induced by BM4 immunization were much lower (main group) or undetectable (recovery group).

**Conclusions:** Upon immunization with BM4, the animals developed a robust IgG immune response. The induced antibodies are cross-reactive with Bet v 1, therefore we hypothesize that BM4 also has the potential to induce strong IgG immune responses in humans. This property is highly relevant as it can contribute to the clinical benefits of AIT via blocking of IgE-mediated capture of allergens.

The research was supported by the University of Salzburg Priority Program “Allergy-Cancer-BioNano Research Centre” and the BM4SIT project (grant number 601763) in the European Union’s Seventh Framework Programme FP7.

#### P60 An efficient approach for recombinant expression and purification of rhinovirus 16 (HRV-16) capsid proteins in Escherichia Coli

##### Sabina Wünschmann^1^, Martin D. Chapman^1^, Sayeh Agah^1^, James Hindley^2^

###### ^1^Indoor Biotechnologies Inc., Charlottesville, VA, USA; ^2^Indoor Biotechnologies, Cardiff, United Kingdom

**Correspondence:** James Hindley - hindleyjp@indoorbiotech.co.uk

*Clinical & Translational Allergy* (*CTA*) 2018, **8(Suppl 1):**P60

**Background:** There is strong evidence that human rhinovirus (HRV) infections and respiratory allergies are the two most significant risk factors for asthma exacerbations leading to acute care visits or hospitalization. Surface-exposed capsid proteins (VP1, VP2, and VP3) are important for binding of HRV to corresponding receptors on human epithelial cells. To facilitate research, vaccine development and diagnosis, we developed an efficient method for homogenous production of HRV capsid proteins in *E. coli*.

**Methods:** HRV-16 capsid proteins were expressed in *E. coli* Rosetta 2 cells under IPTG induction. Proteins were re-folded and purified from the insoluble fraction by stepwise dialysis followed by Immobilized Metal Affinity- and gel-filtration chromatography steps.

**Results:** HRV-16 capsid proteins VP1, VP2, VP3, and VP0 (VP2 plus VP4, as a single poly peptide chain) expressed mainly as insoluble proteins in inclusion bodies, while only small amounts expressed in the soluble fraction. Protein solubility was highly dependent on the presence of 0.5 M l-Arginine in most of the purification and storage buffers. The protein preparations were > 90% pure as assessed by silver-stained SDS-PAGE and western blot analysis using HIS-tag and HRV-16 VP2-specific antibodies.

**Conclusions:** Expression of individual HRV capsid proteins is feasible in *E. coli* and the purified proteins will provide useful tools to study the immune mechanisms involved in rhinovirus-induced asthma exacerbations, epitope mapping, and for diagnostic purposes.

#### P61 Rational design of hypoallergenic Phl P 7 variant for the treatment of Phl P 7-sensitized patients

##### Marianne Raith^1^, Doris Zach^1^, Linda Sonnleitner^2^, Konrad Woroszylo^1^, Margarete Focke-Tejkl^3^, Herbert Wank^1^, Thorsten Graf^4^, Annette Kuehn^4^, Mariona Pascal^5^, Rosa Maria Muñoz-Cano^6^, Judith Wortmann^7^, Walter Keller^7^, Ines Swoboda^1^

###### ^1^Molecular Biotechnology Section, FH Campus Wien, University of Applied Sciences, Vienna, Austria; ^2^Department of Biomedical Analytics, University of Applied Sciences Wiener Neustadt, Wiener Neustadt, Austria; ^3^Division of Immunopathology, Department of Pathophysiology and Allergy Research, Center for Pathophysiology, Infectiology and Immunology, Medical University of Vienna, Vienna, Austria; ^4^Department of Infection and Immunity, Luxembourg Institute of Health, Esch-Sur-Alzette, Luxembourg; ^5^Hospital Clínic de Barcelona, Immunology Department, CDB, IDIBAPS, University of Barcelona, Barcelona, Spain; ^6^Hospital Clínic de Barcelona, Allergy Unit, Pneumology Department, ICR, IDIBAPS, University of Barcelona, Barcelona, Spain; ^7^Institute of Molecular Biosciences, University of Graz, Graz, Austria

**Correspondence:** Marianne Raith - Marianne.Raith@fh-campuswien.ac.at

*Clinical & Translational Allergy* (*CTA*) 2018, **8(Suppl 1):**P61

**Background:** Immunotherapy is the only causative treatment for type I allergies, however, it may cause severe side effects. Development of genetically engineered hypoallergenic molecules offers the possibility to improve the safety of immunotherapy.

**Methods:** Previously, a hypoallergenic variant of the calcium-binding fish allergen parvalbumin was successfully engineered by mutating four calcium-coordinating amino acids. We aimed to analyse, whether mutating the same, highly conserved amino acids in the calcium-binding domains of the grass pollen allergen Phl p 7 would also lead to a hypoallergenic molecule. Recombinant wildtype and mutant Phl p 7 were expressed in Escherichia coli and purified to homogeneity.

**Results:** Analysis of the allergenic activity using sera and blood from Phl p 7 sensitized patients in IgE dot blots and basophil activation tests revealed a drastically reduced IgE reactivity and a strongly reduced allergenicity of the mutant variant. To test whether the Phl p 7 mutant protein is an immunogenic molecule, we immunized rabbits with wildtype and mutant Phl p 7 and tested the sera for the presence of Phl p 7-specific IgG antibodies. We saw that rabbit IgG titers were increasing after immunization and that Phl p 7 mutant IgGs were able to block patients’ IgE binding to the Phl p 7 wildtype protein. Both, the immunogenicity as well as the blocking potential are prerequisites for a potential applicability of the mutant molecule for immunotherapy of Phl p 7-sensitized individuals. Analysis of the protein structures using circular dichroism spectroscopy revealed that both variants were expressed as predominantly alpha-helical folded proteins. However, temperature scan experiments revealed a reduced thermal stability of the mutant. Size exclusion chromatography linked to inductively coupled mass spectrometry showed that the mutant protein has lost its calcium-binding capacity.

**Conclusions:** By mutagenesis of specific amino acids involved in calcium-binding of the grass pollen allergen Phl p 7, we were able to produce an immunogenic molecule which showed diminished IgE reactivity and a highly reduced allergenic activity. We therefore suggest that mutating specific amino acids responsible for the coordination of calcium ions might represent a general strategy to generate hypoallergenic variants of calcium-binding allergens.

#### P62 Immunophenotypic changes induced by successful CpG/Fel D 1-based immunotherapy in a murine asthma model

##### Guillem Montamat^1^, Cathy Leonard^1^, Justine Heckendorn^1^, Olivia Domingues^1^, Caroline Davril^1^, Markus Ollert^1^

###### ^1^Department of Infection and Immunity, Luxembourg Institute of Health (LIH), House of BioHealth, Esch-Sur-Alzette, Luxembourg, Esch-Sur-Alzette, Luxembourg

**Correspondence:** Guillem Montamat - Guillem.Montamat@lih.lu

*Clinical & Translational Allergy* (*CTA*) 2018, **8(Suppl 1):**P62

**Background:** Specific-allergen immunotherapy (SIT) is the only disease-modifying treatment for perineal allergic rhinitis/asthma. SIT can be improved through the use of adjuvants to drive the immune system towards tolerance. Our preliminary results have shown a reduction of several allergic parameters in a well-established murine asthma model of CpG oligodeoxynucleotides (CpG-ODN) based immunotherapy using the major cat allergen: Fel d 1. In order to analyse the immunophenotypic changes after CpG/Fel d 1-based immunotherapy, we performed extensive analysis in the lungs and immune relevant organs by mass cytometry.

**Methods:** BALB/c mice were sensitized i.p. using a mixture of Fel d 1 and aluminium hydroxide. Subsequently the mice received three courses of immunotherapy i.p. using a solution of Fel d 1 and CpG-ODN. Allergen challenge was performed through nasal instillation of Fel d 1 solution to trigger the allergic response in murine airways. Mass cytometry (CyTOF 2) was used to study the cellular phenotypic changes 18 h after the final allergen challenge. A panel of 34 markers was used, including surface markers, transcription factors and cytokines. We applied the 34 marker panel in three organs: lungs (effector organ), mediastinal lymph nodes (draining LN) and spleen (general immune status). Three groups were analysed: allergic mice without SIT, allergic SIT treated mice and untreated control mice (n = 5/group).

**Results:** The analysis of the three different organs showed significant results reflecting an overall tolerogenic environment in the SIT treated mice. T and B cells were less activated in the SIT group compared to allergic mice. NK cells showed a twofold higher production of TNFα in the treated mice with respect to the two other groups. We also found substantial changes in the myeloid compartment with dramatic fivefold decrease in Th2-type macrophage subpopulation and tenfold decrease in mast cells in SIT treated mice compared to the allergic group. This was accompanied by changes in eosinophils and others myeloid cells in the lung parenchyma.

**Conclusions:** Using CyTOF 2, a high throughput and innovative immunophenotyping technology we analysed the immune cell specific changes in a CpG/Fel d 1 SIT model. Our promising results will help to further understand how CpG/allergen SIT treatment modulates the immune system towards tolerance. Our data will help to further develop novel SIT approaches using CpG as adjuvant for patients with perennial rhinitis/asthma.

#### P63 Inquiry about the association of cultivable human skin microbiota with asthma outcomes in a group of children and adolescents of Salvador-Bahia

##### Talita Ferreira^1^, Thainah De Almeida Rocha Abreu^1^, Emília M. M. De Andrade Belitardo^1^, Flávia Sena^1^, Alana Alcantara Galvão^1^, Carolina Silva Santos^1^, Mauricio Barreto^1^, Camila Alexandrina Figueiredo^1^, Luis Gustavo Carvalho Pacheco^1^, Neuza Maria Alcântara-Neves^1^, Carina Da Silva Pinheiro^1^

###### ^1^UFBA, Salvador, Brazil

**Correspondence:** Carina Da Silva Pinheiro - carinasilvapinheiro@gmail.com

*Clinical & Translational Allergy* (*CTA*) 2018, **8(Suppl 1):**P63

**Background:** Asthma is a chronic inflammatory disease of the airways associated with airway remodeling leading to bronchial hyperactivity and mucus hypersecretion. It is assumed that children, who grow up in an environment with a high number of pathogens, protect themselves from allergic sensitization. Thus, to understanding the relationship between skin microbiote diversity with asthma and atopy, the present study aims to evaluate the skin microbiota of individuals in Salvador city, Bahia, belonging to a prospective study of respiratory allergies through metagenomics assays.

**Methods:** The skin bacterial flora was collected from the forearm of 50 individuals, separated in five groups based in the phenotypes of asthma and atopy (Atopic Asthmatic-AA; Atopic Asthmatic Remission-AAR; Non-Atopic Asthmatic-NAA; Non-asthmatic Atopic-ANA; Healthy), grown in BHI medium, and bacterial DNA extracted using a commercial extraction kit. DNA from 16S rRNA region was sequencing in 454 GS-FLX Titanium platform by a specialized company. The identification of genders with modulatory action as Acinetobacter was did using specific primers through PCR, and the absence or presence of this bacteria was associated with the production of the regulatory cytokine IL-10 in blood culture stimulated with antigens of *Ascaris lumbricoides*, *Blomia tropicalis*, Dermatophagoides farinae and vitamin D.

**Results:** The result of the 16S rRNA sequencing of the cultivable skin bacteria resulted in the identification of 27 bacterial genera in the study population. The group of individuals classified as AAR and Healthy were found with the higher biodiversity of genders. The presence of Acinetobacter was confirming in 31 individuals and it was observed a higher production of IL-10 in the culture supernatant stimulated with the *A. lumbricoides* and *B. tropicalis* antigens in the AA group without presence of Acinetobacter. In all the supernatants analyzed it was possible to detect higher levels of IL-10 production in healthy individuals who presented Acinetobacter bacteria when compared to the Healthy group without the presence of this bacteria.

**Conclusions:** The identification of species with modulatory action as Acinetobacter in AAR and Healthy group corroborates the hypothesis that the individual microbiota can influence the development or remission of asthma symptoms. And further studies with a higher number of individuals should be performed to confirm the correlation between the presence of Acinetobacter and the asthma and atopy outcomes.

#### P64 Immune modulation of inflammatory and allergic responses by a TGF-ß1-like peptide

##### Galber R. Araujo^1^, Lorenz Aglas^1^, Emilia R. Vaz^2^, Luiz R. Goulart^2^, Fatima Ferreira^1^

###### ^1^University of Salzburg, Salzburg, Austria; ^2^Federal University of Uberlândia, Uberlândia, Brazil

**Correspondence:** Galber R. Araujo - galber.araujo@gmail.com

*Clinical & Translational Allergy* (*CTA*) 2018, **8(Suppl 1):**P64

**Background:** Transforming growth factor-β1 (TGF-β1) has been shown to exert immunosuppressive functions, as reflected by inhibition of immune cell differentiation (Th1 and Th2 responses) and induction of regulatory T cells (Treg), thus playing an important role against the development of immunological disorders. Hence, peptides that mimic the active core of TGF-β1 could be highly promising candidates for immune modulation. In the present study, a TGF-β1-like peptide was evaluated for its capability of modulating the immune response.

**Methods:** TGF-β1-like peptide was selected by phage display technology through competitive elution with the recombinant TGF-β1. Flow cytometry, ELISA, ELISpot, reporter gene, mediator release, intravital microscopy and peritonitis assays were conducted to evaluate the capacity of the peptide to modulate the in vitro or in vivo immune response under inflammatory or allergic conditions.

**Results:** The synthetic TGF-β1-like peptide was able to decrease TNF-α and increase IL-10 production in human PBMCs, and to decrease IL-8 gene expression and cytokine production in Jurkat cells. In vivo experiments showed that in mice sensitized with Phl p 5, the major allergen from timothy grass pollen, the TGF-β1-like peptide was able to decrease IL-4 and IFN-γ, and increase IL-10 production in murine splenocytes. In the same model, the peptide was also able to decrease basophil degranulation and induce Treg cell differentiation. In another mouse model, the TGF-β1-like peptide was able to decrease leukocyte rolling and neutrophil migration under an inflammatory condition.

**Conclusions:** The TGF-β1-like peptide presented herein was able to induce Treg cell differentiation, modulate Th1 and Th2 responses, as well as other important events that promote the exacerbation of an inflammatory or allergic microenvironment. These findings strongly imply a potential use of the TGF-β1-like peptide as immunomodulatory compound for therapeutic approaches.

**Acknowledgments:** This research was supported by the Brazilian funding agency Conselho Nacional de Desenvolvimento Científico e Tecnológico – CNPq (153753/2015-3), the National Institute of Science and Technology in Theranostics and Nanobiotechnology (CNPq 465669/2014-0), and the Priority Program “Allergy-Cancer-BioNano Research Centre” of University of Salzburg. G.R. Araujo is a recipient of an EAACI Research Fellowship 2017.

#### P65 How the use of molecular allergology can guide us in the diagnosis of specific IgE sensitizations of patients with multiple plant-food allergies, even with a limited availability of allergen components

##### Csilla Csáki^1^, Zsuzsanna Ragó^1^

###### ^1^Svábhegy National Center for Pediatric Pulmonology and Allergology, Budapest, Hungary

**Correspondence:** Csilla Csáki - cscs@euroweb.hu

*Clinical & Translational Allergy* (*CTA*) 2018, **8(Suppl 1):**P65

**Background:** Multiarray allergen technology measuring over one hundred of allergenic molecules proved to be very useful in the diagnosis of patients with multiple specific IgE (sIgE) sensitizations. In multiallergic patients the clinician has the difficult task to differentiate between true allergies and numerous cross-sensitizations, and give meaningful recommendations for avoidance diet and immunotherapy. However, multiplex essays are very expensive and not yet available in countries with limited financial resources or no insurance reimbursement

In the past few years molecular allergy testing has become available for routine clinical practice. Allergy Lateral Flow Assay (ALFA) is a rapid test for qualitative determination of sIgE in human serum, plasma or whole blood. It enables the reliable and cost effective measurement of allergen components on a single-strip or an eight-strip cassette even in a non-hospital based outpatient setting.

**Case Report:** A child was presented to our clinic with symptoms of allergic rhinitis since the age of 2. Parents complained of heavy nasal and eye symptoms with eyelid edema occurring seasonally in early spring and late summer. Consumption of hazelnuts and lentils repeatedly caused the patient lip swelling. After eating peanuts the patient developed vomiting, throat swelling and breathing difficulties. Specific IgE examination with whole extracts was positive to almost all of the tested 30 inhalants and 30 food allergens. Class 6 sIgE (value higher than 100 IU/mL) was detected to peanuts, ragweed, mugwort and birch pollen. Class 3–5 sIgE (3.5–100 IU/mL) was measured to alder, oak, hazelnut, 12-grasses, rye, dust mites, nettle, kiwi, latex and egg white. Based on clinical history the following structural molecular components were tested in order to determine true allergies: Ara h2, Ara h6, Amb a1, Art v1, Phl p1, Phl p5, Hev b5, Cor a9. For cross-reactivity mapping the key components of cross-reactive protein families were tested additionally: Bet v1, Pru p3, Phl p7, Phl p12. A detailed map of cross-reactivities will be presented.

**Conclusions:** How this report contributes to current knowledge: Authors present a diagnostic algorithm developed for molecular allergy testing of patients with multiple plant-food allergies and cross-reactivities. This problem oriented approach enables the clinician to make the correct diagnosis even in circumstances of limited component availability.

#### P66 Sensitization profiles and efficacy of sublingual immunotherapy in children with pollen-food allergy syndrome associated with birch pollen allergy in The Russian Federation (preliminary results)

##### Oksana Ereshko^1^, Leyla Namazova-Baranova^1^, Svetlana Makarova^1^, Elena Vishneva^1^, Marina Snovskaya^1^, Julia Levina^1^, Kamilla Efendieva^1^, Anna Alekseeva^1^

###### ^1^Scientific Centre of Children Health, Moscow, Russia

**Correspondence:** Oksana Ereshko - ksenya2005@inbox.ru

*Clinical & Translational Allergy* (*CTA*) 2018, **8(Suppl 1):**P66

**Background:** Pollen-food allergy syndrome (PFAS) describes allergic reactions on products of vegetable origin in pollen-sensitized individuals. Significant prevalence of allergic rhinoconjunctivitis (ARC) and wide range of clinical manifestations of cross-reactions to food make actual the study of sensitization profiles and sublingual immunotherapy (SLIT) efficacy in these patients.

To evaluate the prevalence of sensitization to recombinant component-resolved allergens (CRA) and SLIT efficiency among children with PFAS in Russian Federation

**Methods:** 54 children (5–18 years) with PFAS were examined. The sIgE assays to birch pollen and to CRA (Bet v1, Bet v2, Bet v4, Bet v6) were performed using ImmunoCap. SLIT was applied with standardized commercial birch pollen extracts.

**Results:** Sensitization to Bet v1 was found in all patients—100%; to Bet v2—17%; to Bet v4—2%; to Bet v6—29%. 52% of patients had monosensibilization to Bet v1 component.

We identified 5 IgE profiles to CRA (group I—Bet v1; II—Bet v1/Bet v6; III—Bet v1/Bet v2; IV—Bet v1/Bet v2/Bet v6; 1 patient had sensibilization to all CRA).

Results: of the SLIT in:

- Group I (n-28): 20 patients had decreased ARC and PFAS symptoms; 3 had decreased only symptoms of ARC.
- Group II (n-13): 7 patients had decreased both ARC and PFAS symptoms; 5 had decreased only symptoms of ARC.
- Group III (n-7): 4 patients had decreased both ARC and PFAS symptoms; 3 had decreased only ARC symptoms.
- Group IV (n-5): 2 patients had decreased both ARC and PFAS symptoms; 3 had decreased only ARC symptoms.

1 patient with sensibilization to all CRA had no improvement after treatment.

**Conclusions:** Bet v6 is the most common minor allergen in children with PFAS associated with birch pollen allergy.

61% of our patients had visible effect of SLIT treatment with decrease of both ARC and PFAS symptoms, 26% had effect on ARC symptoms. Efficiency of SLIT was higher in patients with monosensibilization to Bet v1.

#### P67 Some Haemodynamic data during bronchial asthma in children

##### Liana Jorjoliani^1^, Rusudan Karseladze^1^, Lali Saginadze^1^, Tinatin Bigvava^1^

###### ^1^Iv. Javakhishvili Tbilisi State University, Tbilisi, Georgia

**Correspondence:** Liana Jorjoliani - l_jorjoliani@yahoo.com

*Clinical & Translational Allergy* (*CTA*) 2018, **8(Suppl 1):**P67

**Background:** The chronicle, progressive character of bronchial asthma, the repeated episodes of hypoxemia and hypoxia in bronchial asthma (BA), worsen functional condition of myocardium, which is accompanied by development of pulmonary hypertension. The mentioned thing determines the early diagnostic necessity of transit changes of cardiovascular system in children with BA.

**Methods:** The integral estimation of the cardio-respiratory changes in the children with BA.

The research covered 52 children with BA aged 6–15. In the first group, 22 children aged 6–10 years, the maximum asthma duration was ≥ 6 years. Whereas in the second group 30 children aged 11–15 years, the maximum asthma duration was < 6 years. All subjects were examined by conventional Doppler echocardiography and they had pulmonary function tests on spirometry. The statistic procession of the received data was performed on the bases of the program package SPSS/V.12.

**Results:** Haemodynamic changes characteristics of BA was dependent on duration of the disease. The patients, who had the Doppler echocardiography normal pressure gradient (9–13 mmHg) in the pulmonary artery, had moderate decrease of Spirometric indices (VC, FEV1, IT). The mentioned data were detected in the first group of BA, where the duration of disease was ≥ 6 years. In the second groups of the patients, with < 6 years BA period, Spirometric datum (VC, FEV1, IT) decrease is detected and pressure gradient increase (21–35 mmHg) in pulmonary artery. The reliable negative correlation connection was revealed in the pulmonary artery between the pressure gradient and the most indexes of external respiration.

It is worth mentioning, that there is the moderate connection for IT (r = 0.45, *p* = 0.01). There was no connection revealed in the pulmonary artery between the pressure gradient VC and FEF75. The sensitivity of the mentioned connections turned out low (*p* = 0.05): from 1.1% for FEV1, to 21% for FEF25.

**Conclusions:** These findings signify the diagnostic value of Doppler echocardiography in the early detection and monitoring of such deleterious effects among asthmatic patients.

#### P68 Oropharyngeal symptoms on first exposure to persimmon *Diospyros kaki* in an adult patient with Bet V 1-related allergy

##### Florin-Dan Popescu^1^, Mariana Vieru^1^, Carmen Saviana Ganea^2^

###### ^1^Carol Davila University of Medicine and Pharmacy, Bucharest, Romania; ^2^Nicolae Malaxa Clinical Hospital, Bucharest, Romania

**Correspondence:** Florin-Dan Popescu - florindanpopescu@ymail.com

*Clinical & Translational Allergy* (*CTA*) 2018, **8(Suppl 1)**: P68

**Background:** Allergy to *Diospyros kaki*, commonly called Oriental persimmon, kaki or Sharon fruit (Ebenaceae family), has been very rarely reported. Cross-reactivity with pollen was involved in some cases, being discussed Bet v 1-like Dio k 1, profilin Dio k 4 and Bet v 6-like isoflavone reductase.

**Methods:** We selected a 46-year-old male patient with seasonal allergic rhinoconjunctivitis and convincing history of oral allergy syndrome to hazelnuts, apple and kiwifruit, reporting immediate oropharyngeal allergy symptoms after first eating exposure to raw ripe nonastringent variety of kaki. He never ate other Diospyros fruits, such as American persimmon (*D. virginiana*) or date plum (*D. lotus*). Skin prick testing was performed with commercial allergen extracts and soymilk, while prick–prick testing was done with Bet v 1-containing plant foods: apple, hazelnut, kiwifruit, and persimmon *D. kaki* from South Africa. Multi-parameter line blot immunoassays with native extracts and recombinant molecular allergen components were used for in vitro allergy diagnosis.

**Results:** Regarding Bet v 1-related allergy, the patient presented positive skin prick tests to birch and hazel pollen commercial extracts (each 18 mm diameter wheal), positive prick–prick tests with fresh apple and kaki persimmon (each 4 mm wheal), kiwifruit (3 mm wheal), and hazelnut (6 mm wheal), and negative skin test to soymilk, while serum specific IgE levels were found increased for birch (3.5 kU/L), hazel (0.43 kU/L) and alder (13.4 kU/L) pollen. Serum specific IgE antibodies against Rosaceae (apple, peach) and Actinidiaceae (kiwi) fruits, Betulaceae (hazelnut) and Rosaceae (almond) nuts, Apiaceae vegetables (celery, carrot), Fabaceae legumes (peanut, soybean) and tomato, were not found (< 0.35 kU/L). Specific IgE profile to recombinant components revealed sensitization to rBet v 1 (0.51 kU/L), while serum IgE antibodies to profilin (rBet v 2, rPhl p 12) and polcalcin (rBet v 4, rPhl p 7) biomarkers, and to isoflavone reductase rBet v 6, were not detected (< 0.35 kU/L). Serum IgE level to cross-reactive carbohydrate determinant marker was also below detection (< 0.35 kU/L).

**Conclusions:** Patients with Betulaceae pollen-related food allergies should be asked about oropharyngeal or other allergic symptoms occurring after eating raw foods from a panel list of potentially Bet v 1-cross-reactive plant foods, not only apples and hazelnuts, but also persimmon, a relatively new introduced edible fruit in Europe.

**Consent to publish:** Written informed consent to publish was obtained from the patient involved in this study.

#### P69 Six recurrent Bet V 1 related episodes of anaphylaxis over 6 months. Case report - Think together

##### Zsuzsanna Ragó^1^

###### ^1^Svabhegy National Center of Pediatric Pulmonology and Allergology, Budapest, Hungary

**Correspondence:** Zsuzsanna Ragó - zsuzsanna_rago@yahoo.com

*Clinical & Translational Allergy* (*CTA*) 2018, **8(Suppl 1)**: P69

**Background:** Anaphylaxis is a life threatening allergic reaction, can cause also severe anxiety disorder and bad quality of life for the patients ever experienced it. Recurrent anaphylaxis is very rare in the literature and due to lack of known triggering factors most of them considered idiopathic.

During the 3rd trimester of her 2nd pregnancy a 33 year old women experienced allergic rhinitis symptoms in the BIRCH pollen season. She used no medication. She was otherwise a healthy female and had no atopic disease in her medical history.

**Case Report:** The next birch season while eating RAW FRUITS, like cherry, apple, carrot, plum, celery or peach she experienced swallowing difficulty, and was diagnosed with supraglottic oedema. She had no problem with cooked carrot or celery.

In the 3rd birch season she experienced bad cough while consuming PEANUT.

That time she also had an early miscarriage.

The 4th birch season:

1st ANAPHYLAXIS: 2 min after eating HASELNUT for breakfast in a ski resort she felt itchy and swollen throat, then dizziness and collapsed. No adrenalin was administered by the ambulance staff (Poland).

2nd ANAPHYLAXIS: 1 month later they went to another ski resort in Austria. 2 min after drinking SOY extract another anaphylactic episode occured. Her husband gave her adrenalin shot im. She was hospitalized.

3rd ANAPHYLAXIS: Next day in the hotel she ate only 1 bite of a TUNA salad and immediately felt low blood pressure, dizziness and fainted.

4th ANAPHYLAXIS attacked her next morning with having no meal.

5th ANAPHYLAXIS: 3 month later at home 20 min after eating a BANANA she felt discomfort, light headedness and dizziness. She promptly laid down waiting for the ambulance car.

6th ANAPHYLAXIS: 2 weeks later having OATFLAKES in coconut milk with cacao powder caused the same symptoms as happened before.

She has been undergone many examinations, blood tests, CRD, a bone marrow sample was also taken.

We will unveil her results and her recent treatment. We would appreciate if you could share your opinion with us in order to find the best medical solution.

**Conclusions:** How this report contributes to current knowledge: No such case has been reported to date.

**Consent to publish:** Written informed consent to publish was obtained from the patient involved in this study.

#### P70 Highly structured bioparticles for an immunostimulant allergen presentation technology

##### Véronique M. Gomord^1^, Louis-Philippe Vezina^1^, Anne-Catherine Fitchette^1^, Elizabeth Fixman^2^, Lorna Garnier^3^, Virginie Stordeur^1^, Brian Ward^2^, Sébastien Viel^3^, Guy Tropper^1^, Loic Faye^1^

###### ^1^ANGANY Genetics, Val De Reuil, France; ^2^Mc Gill University, Montreal, Canada; ^3^CHU Lyon, Lyon, France

**Correspondence:** Véronique M Gomord - vgomord@crihan.fr

*Clinical & Translational Allergy* (*CTA*) 2018, **8(Suppl 1)**: P70

**Background:** Nanotechnology is revolutionizing many aspects of modern medicine, including diagnostics and therapeutics. It is now well established that an antigen presented in soluble monomeric form is a relatively weak immunogen, whereas the same antigen presented on the surface of an organized 3D structure will trigger a broader and stronger immune response. From the strong immunogenicity of these 3D structures, it is hypothesized that bioparticles presenting allergens at their surface should enable protective immunization against major allergens with a low number of injections. In this context, we have investigated the efficiency of a new plant-based platform for the production of allergen presenting bioparticles.

**Methods:** A plant host has been devised for the spontaneous expression of allergen presenting bioparticles. This proceeds through transient expression of the fusion of a synthetic non-immunogenic carrier with an allergen component presented in a geometrically repetitive pattern. Bioparticles harboring spikes of oligomerized major dust mite allergen Der p 2 were produced. These bioparticles were purified, characterized and used for a head-to-head mouse immunogenicity and efficacy study in comparison with the same allergen in a soluble form and whole dust mite commercial extracts.

**Results:** Among the great advantages of this new approach to allergen immunotherapy through allergen presenting bioparticles are its features of simplicity and efficiency. These plant produced bioparticles have been designed to elicit an immune response by direct immunostimulation of antigen-presenting cells. They have at their surface a constant and high density of organized allergens. Product quality is easily standardizable and its composition is perfectly reproducible from batch to batch. The production technology is GMP compliant, low cost and has unparalleled scalability. Its functionality has now been tested with several major allergens. These allergen bioparticles contain several hundreds of allergen molecules on their surface. Current trials in murine allergy models suggest their high potential for fast and efficient desensitization.

**Conclusions:** Our current results suggest that plant-made allergen bioparticles have a real potential to create a strong protective immune response against respiratory allergens.

### Oral abstracts: Integrating molecular and cellular biomarkers in diagnostic pathways

#### O03 Distinct parameters of the basophil activation test are useful as biomarkers for the clinical outcome of patients with Alpha-Gal sensitization

##### Jana Mehlich^1^, Jörg Fischer^2^, Christiane Hilger^3^, Martine Morisset^4^, Françoise Codreanu-Morel^4^, Simon Blank^5^, Maximilian Schiener^5^, Kyra Swiontek^3^, Markus Ollert^3^, Ulf Darsow^1^, Tilo Biedermann^1^, Bernadette Eberlein^1^

###### ^1^Department of Dermatology and Allergy Biederstein, Klinikum rechts der Isar, Technical University of Munich, Munich, Germany; ^2^Department of Dermatology, Universitätsklinikum Tübingen, Tübingen, Germany; ^3^Department of Infection and Immunity, Luxembourg Institute of Health (LIH), Esch-Sur-Alzette, Luxembourg; ^4^Immunology-Allergology Unit, Centre Hospitalier, Luxembourg, Luxembourg; ^5^Center of Allergy and Environment (ZAUM) Technical University of Munich and Helmholtz Center Munich, Munich, Germany

**Correspondence:** Jana Mehlich - jana.mehlich@aol.com

*Clinical & Translational Allergy* (*CTA*) 2018, **8(Suppl 1)**: O03

**Background:** The alpha-gal syndrome is characterized by a delayed type 1 allergic reaction to the carbohydrate galactose-alpha-1,3-galactose (alpha-gal) after consumption of mammalian (red) meat products, some drugs of mammalian origin e.g. cetuximab or gelafundin and the presence of specific IgE antibodies directed to alpha-gal. Diagnostics currently rely on detailed patient history, skin prick tests, the determination of specific IgE antibodies (e.g. to alpha-gal, pork, beef, milk protein) and oral food challenges. Objective: Assess the utility of different basophil parameters (basophil reactivity, basophil sensitivity, i.a.) as biomarkers of the clinical outcome of patients with alpha-gal syndrome compared with individuals with alpha-gal sensitization (alpha-gal sIgE antibody ≥ 0.10 kU/L) without clear history to improve diagnostics and care for patients.

**Methods:** Skin tests with different alpha-gal containing substances were performed and specific alpha-gal IgE antibodies were determined in all individuals. A basophil activation test (Flow CAST^®^) with different concentrations of several allergens (e.g. commercially available alpha-gal-compounds, pork, beef, pork kidney extracts, pork-derived medical preparations) was performed in 51 individuals (21 patients with alpha-gal syndrome, 12 alpha-gal sensitized individuals, 18 controls). Basophil activation expressed in percentage of CD63 activated cells was measured and basophil sensitivity (EC50, CD-sens) as well as the quotient of %CD63 +/Anti-IgE (anti-FcεRI antibody) were calculated.

**Results:** Pork kidney extract, commercially available alpha-gal-compounds and pork-derived medical preparations induced a high basophil activation in a dose-dependent manner. Basophil activation was significantly higher in patients with alpha-gal-syndrome compared to sensitized individuals at distinct allergen concentrations. The pork kidney extract produced a significantly higher CD-sens value in patients with alpha-gal-syndrome (*p* = 0.001). %CD63 +/Anti-IgE was significantly higher in patients with alpha-gal-syndrome across most concentrations of all tested allergens. In basophils of controls no activation was detected.

**Conclusions:** Distinct parameters of the basophil activation test displayed significant differences between patients with alpha-gal-syndrome compared to individuals with alpha-gal sensitization. The basophil activation test should therefore be considered an as additional diagnostic test before performing time-consuming and risky oral provocation tests.

#### O04 Diagnostic value of Recombinant Ara H 2 isoforms and derived synthetic peptides in peanut allergic versus sensitized but clinically tolerant children

##### Jasmin Popp^1^, Valérie Trendelenburg^2^, Bodo Niggemann^2^, Stefanie Randow^1^, Elke Völker^1^, Jelena Spiric^1^, Andreas Reuter^1^, Dirk Schiller^1^, Stefan Vieths^3^, Kirsten Beyer^2^, Thomas Holzhauser^1^

###### ^1^Paul-Ehrlich-Institut, Division of Allergology, Langen, Germany; ^2^Charité Universitätsmedizin, Department of Pediatric Pneumology and Immunology, Berlin, Germany; ^3^Paul-Ehrlich-Institut, Division of Allergology, Vice President’s Research Group, Langen, Germany

**Correspondence:** Jasmin Popp - Jasmin.Popp@pei.de

*Clinical & Translational Allergy* (*CTA*) 2018, **8(Suppl 1)**: O04

**Background:** Ara h 2 is a major allergen with high diagnostic value in peanut allergy. The diagnostic value of the individual Ara h 2 isoforms in direct comparison to Ara h 2-derived synthetic peptides has not been investigated within one study group so far. Thus, we aimed at comparing IgE binding and diagnostic value of the recombinant mature isoforms rAra h 2.01 and rAra h 2.02, and of derived synthetic peptides in peanut-allergic versus sensitized but clinically tolerant children.

**Methods:** 35 children with peanut-specific IgE ≥ 0.35 kUA/L (ThermoFisher ImmunoCAP) were included in the study. 23 children were allergic and 12 clinically tolerant to peanut. Recombinant mature Ara h 2 isoforms were expressed in Pichia pastoris. Serum IgE binding to rAra h 2.01 and rAra h 2.02 was determined in immunoblot analysis. 15-mer overlapping peptides (offset 4 aa) representing the entire amino acid sequence of each isoform were synthesized on a cellulose matrix. IgE binding to peptides was analyzed on CelluspotTM multipeptide microarrays. IgE binding to hydroxylated proline residues was additionally investigated. The diagnostic value of rAra h 2.01, rAra h 2.02, and of Ara h 2 peptides was determined as area under curve (AUC) by receiver operating characteristic (ROC) curve analysis.

**Results:** rAra h 2.01 and rAra h 2.02 bound serum IgE of 15/23 (65%) and 17/23 (74%) peanut-allergic children, respectively. Serum IgE of peanut sensitized but tolerant children did not bind to the Ara h 2 isoforms. Serum IgE to peanut extract had the lowest AUC (0.79) compared to IgE that bound to rAra h 2.01 (0.93) and rAra h 2.02 (0.95). IgE binding to selected Ara h 2 peptides correlated well with IgE binding to mature rAra h 2 isoforms and was comparably sensitive. Hydroxylation of proline residues increased peptide-IgE binding in 12/23 peanut allergic children. Two peptide pairs with AUC (0.91–0.93) comparable to recombinant Ara h 2.01/2.02 (0.93–0.95) were identified.

**Conclusions:** In this study group, rAra h 2.02 had the highest diagnostic value for peanut allergy. The diagnostic value of two peptide pairs of Ara h 2 was comparable to rAra h 2. Theses peptides, if verified in a prospective study may serve as peptide biomarkers in the diagnosis of peanut allergy.

### Oral abstracts: Natural tolerance induction and immune intervention

#### O05 Ex vivo and in vivo analyses of early immune events induced by CpG-Based immunotherapy in a mouse model of allergy to Fel D 1

##### Cathy Leonard^1^, Justine Heckendorn^1^, Guillem Montamat^1^, Olivia Domingues^1^, Caroline Davril^1^, Markus Ollert^1^

###### ^1^Department of Infection and Immunity, Luxembourg Institute of Health (LIH), Esch-Sur-Alzette, Luxembourg

**Correspondence:** Cathy Leonard - cathy.leonard@lih.lu

*Clinical & Translational Allergy* (*CTA*) 2018, **8(Suppl 1)**: O05

**Background:** CpG-ODN are used as adjuvant for their propensity to induce effector Th1 cells and reverse allergic immune responses. Our preliminary data showed in an experimental model of asthma to Fel d 1 that Fel d 1+ CpG specific immunotherapy (SIT) efficiently induced tolerance to Fel d 1 challenge with an unexpected role for TNF-α. In order to identify the actors and mechanisms of this unconventional tolerizing reaction, we investigated the types of cells responsive to CpG and analysed the early immune events during CpG/Fel d 1-based SIT.

**Methods:** Cells isolated from the peritoneal cavity and spleen of naïve or sensitized mice (3 i.p. injections with Fel d 1+ Alum) were submitted to increasing concentrations of CpG and analysed for the secretion of TGF-β and TNF-α by ELISA. The key immune cell populations (DCs, B cells, T cells, macrophages [MF]) were investigated by flow cytometry. In an in vivo approach, mice were sensitized to Fel d 1 and received one i.p. immunotherapy injection. Cells were collected 24 h after injection from the peritoneal cavity and spleen and analysed in depth via mass cytometry (CyTOF-2, 34 markers). Corresponding organs from control and allergic mice (sensitized but not SIT-treated) were also investigated.

**Results:** TNF-α was shown to be secreted ex vivo already 6 h after incubation with CpG, in a dose-dependant way, by cells from peritoneal cavity and splenic lymphocytes. No TGF-β was detectable. Plasmacytoid DCs (pDCs), B cells and MF were identified by FACS to be among the major TNF-α producers after CpG stimulation. Analysis of CyTOF data showed that pDCs and MF subpopulations of the peritoneal cavity were reduced 1 day after SIT injection, suggesting their migration to immune organs. In the spleen, B cells and T cells were strongly activated 24 h post injection. B cells were confirmed to be TNF-α positive, together with a previously not observed NK cell subpopulation, also stimulated by SIT.

**Conclusions:** A remodeling of antigen-presenting cell subpopulations (pDCs/MF) at the site of injection (i.p.) as well as a robust stimulation of B, T and NK cells in the spleen were observed at short term 24 h after a first CpG-based SIT injection. Further examination of the collected data, combined with similar analyses applied after a complete round of 3 SIT courses, will further clarify the tolerizing mechanism induced by CpG/Fel d 1 SIT. These data will help to optimize novel forms of SIT for patients with perennial rhinitis/asthma.

#### O06 An engineered IgE-Fc variant inhibits basophil degranulation ex vivo

##### Pascal Gasser^1^, Luke Pennington^2^, Daniel Brigger^3^, Noemi Zbären^3^, Theodore Jardetzky^2^, Alexander Eggel^3^

###### ^1^Department for BioMedical Research, University of Bern/Department of Rheumatology, Immunology and Allergology, University Hospital Bern, Bern, Switzerland; ^2^Department of Structural Biology, Stanford University School of Medicine, Palo Alto, CA, USA; ^3^Department for BioMedical Research, University of Bern/Department of Rheumatology, Immunology and Allergology, University Hospital Bern, Bern, Switzerland

**Correspondence:** Pascal Gasser - pascal.gasser@dkf.unibe.ch

*Clinical & Translational Allergy* (*CTA*) 2018, **8(Suppl 1)**: O06

**Background:** Allergen-specific IgE plays a major role in the development of allergic reactions. It binds with high-affinity to the primary IgE receptor FcεRI on basophils and mast cells. Upon exposure to the cognate allergen IgE-loaded cells immediately degranulate and release soluble mediators causing allergic symptoms. The therapeutic anti-IgE antibody Omalizumab is known to neutralize free IgE and to prevent binding of IgE to basophils and mast cells. Recently, we have reported that Omalizumab actively desensitizes basophils at high concentrations. Furthermore, we have provided evidence that a mutated IgE-Fc variant, which is resistant to Omalizumab binding, may be used to actively replace the IgE-repertoire on the surface of primary human basophils when co-applied with Omalizumab. This combination treatment significantly increased inhibition of antigen-mediated basophil activation ex vivo. Here, we aim to further investigate the exact mechanism of basophil inhibition for the mutated IgE-Fc variant.

**Methods:** Human primary basophils were isolated from whole blood donations of grass-pollen allergic individuals and treated with wiltype IgE-Fc or mutated IgE-Fc variants alone or in combination with Omalizumab. Subsequently, cells were challenged with a grass-pollen allergen mix. Basophil activation was measured by flow cytometry.

**Results:** Interestingly, the mutated IgE-Fc variant alone diminished basophil activation in a competition-independent manner, while the wildtype IgE-Fc variant showed no effect. Furthermore, the IgE-Fc variant showed synergistic and dose-dependent inhibition already at low concentrations when used in combination with Omalizumab.

**Conclusions:** Our data indicate that the mutated IgE-Fc variant might engage an inhibitory receptor on the surface of basophils. However, further studies are required to confirm this hypothesis. The IgE-Fc variant could potentially be used as an efficient add-on treatment to the current Omalizumab therapy.

### Oral abstracts: Molecular diagnostics in clinical management

#### O07 Towards a more complete allergen panel for component-resolved diagnosis of walnut allergy

##### Serge Versteeg, Ronald Van Ree, The Europrevall Consortium

###### Academic Medical Center, University of Amsterdam, Amsterdam, the Netherlands

**Correspondence:** Serge Versteeg - s.a.versteeg@amc.uva.nl

*Clinical & Translational Allergy* (*CTA*) 2018, **8(Suppl 1)**: O07

**Background:** The English walnut, *Juglans regia* (*J. regia*), is an important tree nut associated with food allergy. Recombinant production of the major walnut allergens will allow component resolved diagnosis (CRD) to complement walnut extract-based tests that often have insufficient sensitivity.

To express and characterize all walnut allergens known to date as recombinant proteins and perform a walnut CRD study in patients with reported adverse reactions to walnut, recruited at 12 clinical centers across Europe (EuroPrevall outpatient clinic survey).

**Methods:** Walnut 2S albumin (rJug r 1) and LTP (rJug r 3) were already commercially available. Walnut profilin, 7S globulin (rJug r 2) and a PR10 isoform (rJug r 5) were cloned and expressed in *E. coli*, purified and characterized by SDS-PAGE, immunoblot and ImmunoCAP. Patients with a well-documented history of walnut allergy were included (n = 225). All patients were tested by ImmunoCAP to walnut and to the resulting panel of five available recombinant walnut allergens.

**Results:** Walnut profilin cDNA encoding a protein of 131 amino acids was cloned into pSUMOpro3 and expressed in *E. coli*. Sequence homology with other profilins (Ara h 5, Cor a 2, Gly m 3, Bet v 2 and Phl p 12) ranged from 80 to 87%. Recombinant Jug r 2 was expressed as a precursor protein of 70 kDa as shown by SDS-PAGE. Recombinant Jug r 5, a Bet v 1 homologue with 84% homology to another recently published isoform (A. Wangorsch et al. 2017), was cloned and expressed in *E. coli*.

Specific (s)IgE against walnut and the five walnut allergens was measured: 22/217 patients (10.1%) were positive for rJug r 1 (> 0.35 kUA/L), 20/211 (9.5%) for rJug 2, 29/217 (13.4%) for rJug r 3, 134/225 (59.6%) for Jug r 5 and 48/217 (22.1%) for walnut profilin. The vast majority of patients (mainly) sensitized to Jug r 5 and/or profilin were not or poorly picked up by extract ImmunoCAP. Only ~ 40% of the 225 patients had detectable IgE against walnut extract.

**Conclusions:** CRD significantly improves sensitivity to detect sensitization to walnut. Walnut PR10 is the most frequently recognized allergen followed by profilin. Sensitization to storage proteins is far less common (~ 10%) and often seen together with that to pollen-associated allergens. Development of two missing molecular allergen reagents (rJug r 4 and walnut oleosin) is ongoing. Analyses will be carried out to associate molecular sensitization profiles with severity of reported (and DBPCFC-induced) reactions.

#### O08 A more accurate approach for the molecular diagnosis of the tomato allergy

##### Laura Martin-Pedraza^1^, Cristina Bueno Díaz^1^, Andrea Wangorsch^2^, Carlos Pastor Vargas^3^, Javier Cuesta-Herranz^3^, Stephan Scheurer^2^, Mayte Villalba Díaz^1^

###### ^1^Universidad Complutense de Madrid, Bioquímica y Biología Molecular I, Madrid, Spain; ^2^Paul-Ehrlich-Institute, Molekulare Allergologie, Langen (Hessen), Germany; ^3^Hospital Funfación Jiménez Díaz, Madrid, Spain

**Correspondence:** Laura Martin-Pedraza - lauramp627@gmail.com

*Clinical & Translational Allergy* (*CTA*) 2018, **8(Suppl 1)**: O08

**Background:** Several clinical reports of patients allergic to certain foods without positive in vitro diagnosis tests with their corresponding commercial extracts, have required the identification of new allergens located in specific tissues poorly represented in the whole extract to clarify the diagnosis of these particular food allergic-patients. Two different non-specific lipid transfer proteins (nsLTPs) have been specifically identified in tomato seeds: Sola l 6 and Sola l 7, not present in the peel or pulp of this fruit where the nsLTP, Sola l 3, is described as the main allergen responsible of the IgE sensitization of patients with allergic symptoms to this vegetable.

The main objective of this study is to analyse if there is an independent sensitization to these tomato nsLTPs or if the cross-reactivity could be involved in the sensitizations mediated by these allergens and with other vegetables extracts using the three purified allergens and evaluating the recognition with polyclonal antibodies (pAbs).

**Methods:** Extracts from different tomato tissues, other vegetables seeds, nuts or Rosaceae members and purified nsLTPs–nSola l 3, rPru p 3, and rSin a 3-, were available; recombinant forms of tomato seed nsLTP, -rSola l 6 and rSola l 7-, have been produced in Pichia pastoris, purified and characterized. pAbs against rSola l 7 and rSola l 6 allow us to determine IgG recognition levels by immunoblotting and ELISA techniques and the possible cross-reactivity between them and with other nsLTPs.

**Results:** IgE recognition of recombinant rSola l 7 and rSola l 6 matched perfectly with the natural forms of these allergens. In vitro IgG recognition to other vegetables extract and purified proteins reveals a great cross-reactivity with Pru p 3, the major allergen from peach. By contrast, no cross-reactivity is observed with Sola l 3, tomato peel nsLTP, neither between Sola l 6 and Sola l 7 despite they belong to the same fruit.

**Conclusions:** The availability of a complete pattern of allergens either recombinant or natural, from the same source is an important approach in order to improve patient molecular diagnosis by in vitro techniques. The results of this study with the specific pAbs lead us to believe that the presence of different proteins of the same family located in different tissue of the same fruit with no IgG cross-reactivity between them deeply confirm previous studies where an independent patient sensitization to these allergens is described.

